# Sensing Underwater: Diversifying Selection, Convergent Evolution and Inactivation in Sensory Receptors’ Genes of Aquatic Mammals

**DOI:** 10.1007/s00239-026-10327-2

**Published:** 2026-07-01

**Authors:** Ana Luiza Lein-Borba, Giovanna Selleghin-Veiga, Beatriz Daros, Letícia Magpali, Mariana F. Nery

**Affiliations:** 1 Department of Genetics, Evolution, Microbiology and Immunology, University of Campinas, Campinas, SP Brazil; 2 Department of Biology, Dalhousie University, Halifax, NS Canada

**Keywords:** Evolutionary genetics, Bioinformatics, Aquatic mammals, Sensory ecology, Transient receptor potential channels

## Abstract

Cetaceans and sirenians independently transitioned from land to water, evolving unique and convergent sensory adaptations shaped by aquatic environments. Among sensory receptors, the Transient Receptor Potential (TRP) channel superfamily is central to thermo-, chemo-, and mechanosensation, but its evolutionary history in fully aquatic mammals remains poorly characterized. Here, we investigated the molecular evolution of TRP channels in these lineages. Orthology and phylogenetic relationships were inferred using Maximum Likelihood and Bayesian approaches. Signals of positive selection and molecular convergence were evaluated with codon and amino acid models. Amino acid substitutions, protein structure, and stability were assessed using 3D protein modeling. Our analyses reveal accelerated evolutionary rates in aquatic mammals, including multiple positively selected sites, lineage-specific amino acid substitutions, and convergent evolution across cetaceans and sirenians. Protein-level assessments identified substitutions with potential functional consequences, and evidence of pseudogenization was detected in cetacean PKD1L3, PKD2L1, TRPA1, and TRPM5, in contrast to intact copies in sirenians. These patterns suggest lineage-specific sensory trajectories, including reduced chemosensory repertoires in cetaceans, conservation of taste-related genes in sirenians, and adaptations in somatosensory associated genes that reflect both convergent requirements of fully underwater living and distinct aquatic environments. Overall, our findings advance understanding of the molecular mechanisms underlying sensory evolution during the land-to-water transition in mammals.

**Supplementary Information:**

The online version contains supplementary material available at 10.1007/s00239-026-10327-2.

## Introduction

Sensory systems are fundamental for survival, allowing organisms to perceive and respond to environmental stimuli through specialized organs, cells, and neural pathways. The primary sensory modalities in vertebrates include somatoreception (mechano and thermoreception), hearing, chemoreception (olfaction and gustation), and photoreception (vision) (Moller 2003). At the genetic level, most sensory receptors belong to large gene families shaped by duplication, diversification, pseudogenization, and lineage-specific expansions, processes driven by both adaptive and neutral evolutionary forces (Nery et al. 2026; Nei et al. 2008; Baldwin and Ko 2020).

**Fig. 1:**
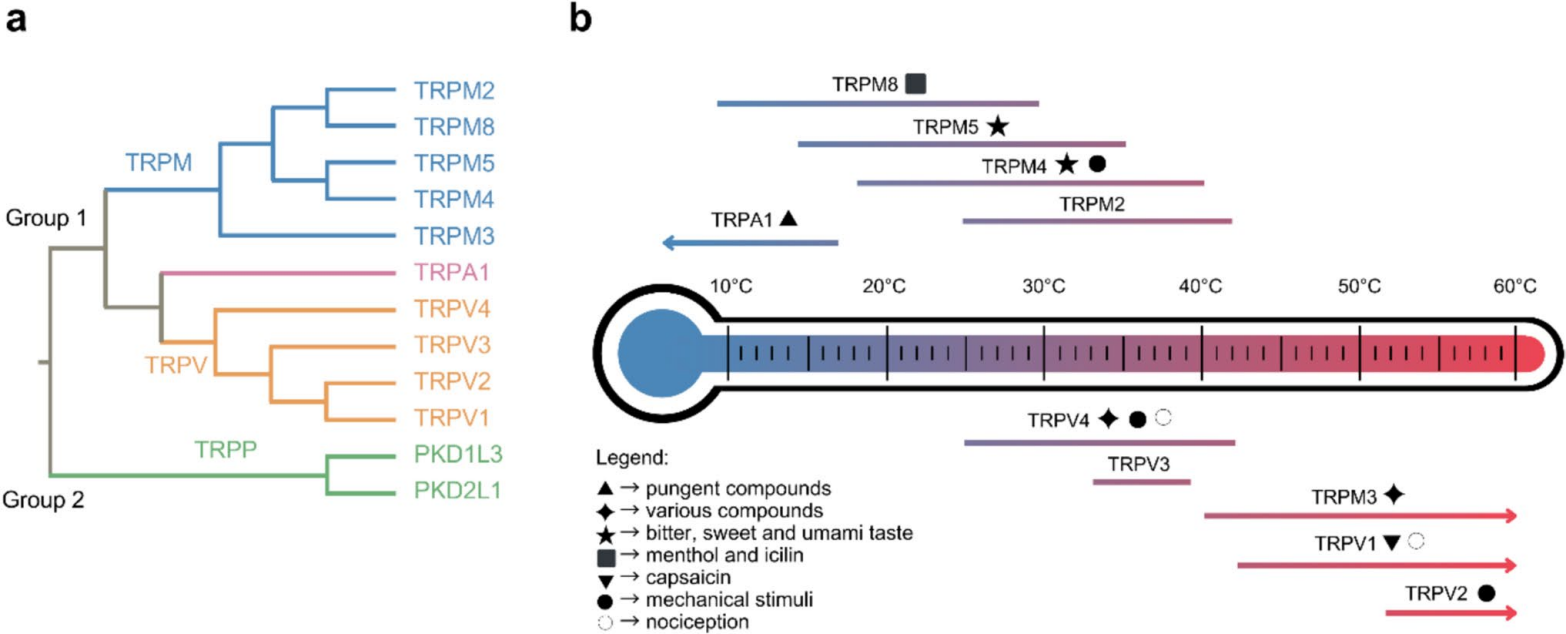
**a** Mammalian TRP superfamily tree showing relationships among channels. Branch lengths are not drawn to scale. Based on Hsiao et al. (2021) and Cabezas-Bratesco et al. (2022). **b** Sensory functions and temperature activation ranges of mammalian thermo-TRPs. Threshold temperatures for each channel are shown using a color gradient. Symbols beside each receptor’s name indicate chemo- and mechanosensory functions

Among the sensory receptors, Transient Receptor Potential (TRP) channels represent one of the best-studied receptor families in amniotes (Moller 2003; Kadowaki 2015; Hoffstaetter et al. 2018) (Fig. 1a). They are highly conserved ion channels broadly expressed across eukaryotes, characterized by six transmembrane domains and assembling as homo or hetero-tetramers to form cation-permeable channels (Na⁺, Ca^2^⁺). Functioning as multimodal receptors, TRPs participate in somatoreception and chemoreception, and also mediate diverse physiological processes (Hoffstaetter et al. 2018) (Fig. 1b).

Somatoreception, as stated before, comprises the detection of mechanical and thermal stimuli. ThermoTRPs (TRPA, TRPM, TRPV) are central to thermosensation. By detecting temperature changes and triggering thermoregulatory responses, they are essential for maintaining homeothermy. Each ThermoTRP is tuned to a specific temperature range (Fig. 1b), and interspecific variation in their sensitivities illustrates evolutionary divergence in thermal responsiveness (Laursen et al. 2016; Hoffstaetter et al. 2018; Himmel and Cox 2020).

Chemoreception involves the detection of chemical stimuli through gustation (taste) and olfaction (smell). Most vertebrates recognize five basic taste modalities: sweet, bitter, salty, umami, and sour. Sweet, bitter, and umami tastes are primarily mediated by T1R and T2R receptors, with TRPM4 and TRPM5 acting as essential downstream transducers (Dutta Banik et al. 2018; Ren and Liu 2020; Nishihara et al. 2024). Sour taste, in turn, relies on multiple mechanisms and receptors, including the TRP heteromer PKD1L3–PKD2L1 (Ishimaru et al. 2006).

A major driver of evolutionary change in sensory systems is environmental transition, as it exposes organisms to distinct biotic and abiotic factors, introducing novel stimuli and ecological interactions. In this context, sensory receptors often evolve under accelerated evolutionary dynamics, leading to species-specific structural and functional differences that reflect ecological and dietary adaptations (Jiang et al. 2012; Lynch et al. 2015; Tigano et al. 2020; Policarpo et al. 2024). A notable example is the repeated transitions to aquatic habitats, which exposed vertebrates to novel selective pressures (Nei et al. 2008; Yuan et al. 2021). As a result, their sensory systems have been reshaped to meet the unique challenges of aquatic environments, where the transmission of light, sound, and chemical cues differs markedly from air (Vermeij and Motani 2018; Davis 2019).

Two mammalian lineages that independently transitioned to aquatic environments are Cetacea and Sirenia, both of which originated during the Eocene (~ 53–57 million years ago). Cetaceans comprise two major clades, Mysticeti (baleen whales) and Odontoceti (toothed whales, including dolphins and porpoises), whereas sirenians include manatees and dugongs. Their independent transitions to aquatic life provide classic examples of convergent evolution, making them valuable models for comparative genomic investigations of sensory system evolution (Berta et al. 2007; McGowen et al. 2014; Yuan et al. 2021). Among the many adaptations linked to the transition to aquatic life, their sensory systems underwent extensive reshaping, including reduced reliance on vision (McGowen et al. 2020b; Yuan et al. 2021; Reidenberg and Hanke 2022), modifications in chemical stimulus detection (Liu et al. 2019; Policarpo et al. 2024), and enhanced mechanoreception (Thewissen and Nummela 2008; Bauer and Reep 2018; Vermeij and Motani 2018).

Taste and olfaction were also strongly modified. In cetaceans, dietary and ecological shifts likely drove a drastic reduction of gustatory and olfactory functions (Kishida 2021; Policarpo et al. 2024). They have fewer taste buds and extensive pseudogenization of T1R and T2R genes, resulting in the loss of sweet, umami, and bitter taste perception (Jiang et al. 2012; Li and Zhang 2014; Zhu et al. 2014)(Jiang et al. 2012; Kishida et al. 2015; Policarpo et al. 2024), while sour taste sensitivity may have been lost through inactivation of PKD2L1 (Feng et al. 2014; Zhu et al. 2014). These changes reflect reduced reliance on taste for food acquisition (Jiang et al. 2012; Feng et al. 2014). In contrast, sirenians retain numerous taste buds and rely on taste for their herbivorous diet (Berta et al. 2007; Li and Zhang 2014; Moore et al. 2022), with functional T1R genes but mostly pseudogenized T2R genes (Li and Zhang 2014; Policarpo et al. 2024).

Tactile perception is crucial for foraging, navigation, and social interactions. Although cetaceans have lost most body hair, mysticetes retain tactile vibrissae, and in both groups the skin supports tactile and proprioceptive functions (Mercado 2014; Bauer and Reep 2018; Springer et al. 2021; Reidenberg and Hanke 2022). Sirenians display highly developed tactile abilities, using their snout, skin, and body-wide vibrissae to interact with the environment. Thermoreception, though less studied, remains essential for thermoregulation and tissue protection (Berta et al. 2007; Bauer and Reep 2018; Moore et al. 2022).

Comparative genomic studies of representative aquatic species can provide key insights into how natural selection shapes sensory evolution. In particular, despite detailed knowledge in some species, the evolutionary history of TRP genes in aquatic mammals remains poorly understood, and chemoreception and somatoreception receive less attention than vision and hearing. To address this gap, we analyzed the molecular evolution of TRP genes in cetaceans and sirenians, aiming to assess the role of natural selection, reveal the molecular basis of convergent traits, and identify genes with evidence of inactivation in these lineages.

## Material and Methods

### Sampling and Genomic Data Collection

We analyzed 12 genes encoding TRP receptors: TRPA1, TRPM2, TRPM3, TRPM4, TRPM5, TRPM8, TRPV1, TRPV2, TRPV3, TRPV4, PKD1L3 and PKD2L1. We focused on fully aquatic lineages, sampling 23 cetacean species (16 odontocetes and 7 mysticetes) and all four extant sirenians, with other mammals from Laurasiatheria and Afrotheria included as outgroups (Table S1).

The coding sequences (CDS) for each gene were retrieved from annotated reference genomes available in the NCBI database (https://www.ncbi.nlm.nih.gov/) (Table S1) (https://www.ncbi.nlm.nih.gov/) and the Bowhead Whale Genome Resource (https://www.bowhead-whale.org/) (Keane et al. 2015). To ensure data quality, we selected the longest and most complete available fragments for analysis. The CDS of the humpback whale (*Megaptera novaeangliae*, GCA_041834305.1), Amazonian manatee (*Trichechus inunguis*, GCA_046562895.1), and dugong (*Dugong dugon*, GCA_030035585.1), whose genomes are not annotated, were retrieved using BLAST (Altschul et al. 1990) in NCBI (https://blast.ncbi.nlm.nih.gov/Blast.cgi). Genome assemblies were queried with sequences from closely related species as references, and the same strategy was applied to recover unannotated or missing sequences in other species. Hits were filtered using an e-value < 0.05 and identity > 90% as selection criteria. For the African manatee (*Trichechus senegalensis*) (Huang et al. 2024), custom Python and Bash scripts were developed to retrieve TRP gene CDSs through BLAST searches, applying the same thresholds. All the fragments retrieved by BLAST were subsequently mapped to reference sequences and assembled in Geneious Prime (https://www.geneious.com), where sequence quality and similarity to the queries were verified.

### Alignments and Identification of Inactivating Mutations

Nucleotide and amino acid sequences were aligned by codons using MACSE v2 (Ranwez et al. 2018). Only species with the most complete sequences were retained, while those with < 50% coverage or poor alignment quality were excluded. Additionally, alignment columns containing indels that introduced gaps in more than 50% of the species were removed, and only well-conserved blocks were retained for downstream analyses. After filtering, sequences were realigned. Due to the filtering steps during data collection and alignment, some genes yielded more species sequences than others; however, all final alignments included representatives of odontocetes, mysticetes, sirenians, and outgroups.

To avoid poorly aligned regions and preserve the reading frame, each alignment was manually inspected to detect errors, start/stop codon mutations, exon or gene deletions, premature stop codons, and frameshift indels in regions conserved across outgroups, as an indicator of pseudogenization. A second alignment was then generated in MACSE, excluding sequences with any inactivating mutations, as pseudogenes evolve neutrally through mutation accumulation (Li et al. 1981; Futuyma and Kirkpatrick 2017). This alignment was subsequently used for phylogenetic reconstruction and molecular evolution analyses.

To further assess pseudogenization, we used the NCBI Genome Data Viewer to compare cetacean sequences with inactivating mutations with *Bos taurus*, with intact genes. Conserved genes and their neighborhood genes were first identified in the non-pseudogenized species, and synteny was then examined in the pseudogenized taxa. When synteny was preserved, BLAST searches spanning the flanking genes were performed to confirm the genomic location of the annotated pseudogenized sequence.

### Phylogenetic Inference

We reconstructed the evolutionary history of the genes through phylogenetic trees based on nucleotide and amino acid alignments. Maximum likelihood analyses were performed in IQ-Tree 2 (Minh et al. 2020) with 1,000 bootstrap replicates, applying the best-fit substitution model for each gene identified by ModelFinder (Kalyaanamoorthy et al. 2017). Nodes with bootstrap ≥ 80 were considered robust. As a complementary approach to increase confidence in the inferred topologies, Bayesian phylogenetic inference with Markov Chain Monte Carlo (MCMC) methods was performed in MrBayes v3.2.7a (Ronquist et al. 2012), with partitioning schemes and substitution models selected by PartitionFinder v2.1.1 under the Bayesian Information Criterion (BIC) (Lanfear et al. 2016). Analyses ran for 10 million generations, sampling every 100.

### Natural Selection Analyses

To assess positive selection in aquatic mammal lineages, we estimated the ratio of non-synonymous to synonymous substitution rates (ω = dN/dS) under a maximum likelihood framework using the CODEML program in PAML 4.4 (Yang 2007) (Goldman and Yang 1994). Values of ω > 1 indicate positive selection, 0 < ω < 1 indicate purifying selection, and ω ≈ 1 indicates neutral evolution, where synonymous and non-synonymous substitutions are expected to fix at similar rates in the absence of selective pressure (Hughes and Nei 1988). For the analyses, we used edited codon alignments from MACSE and species trees from Upham et al. (2019) for Placentalia, McGowen et al. (2020a) for Cetacea, and Heritage and Seiffert (2022) for Sirenia. Both branch (BM) and branch-site (BSM) models were applied, as explained below.

#### CODEML Selection Analyses

We first applied CODEML branch models, to allow ω to vary across predetermined phylogenetic branches and test whether genes in aquatic lineages exhibit accelerated evolution compared to terrestrial lineages (Yang 1998; Yang and Nielsen 1998). The null model, one-ratio (1ω), estimates a single ω for all branches, while the subsequent alternative models assign distinct ω values to foreground (aquatic) and background (terrestrial) branches. Foreground branches were defined under four hypotheses: (2ω) Cetacea and Sirenia (to test for convergence); (3ω) Cetacea vs. Sirenia; (5ω) crown vs. stem Cetacea and Sirenia; and (7ω) further separating Mysticeti vs. Odontoceti, and the Sirenia families, Trichechidae vs. Dugongidae. To control for phylogenetic bias, we examined four analogous models grouping Cetacea and Hippopotamidae (Whippomorpha) and Sirenia and Proboscidea (Tethytheria): (2ω) Whippomorpha vs. Tethytheria (convergence), (3ω) separate ω for each group, (5ω) crown vs. stem within each group, and (7ω) further separating Cetacea and Hippopotamidae, as well as Sirenia and Proboscidea. ω values were estimated for the designated branches in each model based on the species' evolutionary history.

Finally, likelihood ratio tests (LRTs) were used to identify the best-fitting nested models by comparing the likelihoods of null (*l*_0_) and alternative (*l*_1_) models using X^2^ = 2(*l*_1_ − *l*_0_) (Yang and Nielsen 1998; Yang 1998). LRTs follow a χ^2^ distribution, with *p*-values < 0.05 considered significant, supporting the alternative hypotheses.

Next, we applied the branch-site model A (BSMA) to detect positive selection at specific sites within particular lineages (Zhang et al. 2005). In this model, ω varies across sites and branches, but positive selection is restricted to a subset of sites within the foreground branches (aquatic mammals), while in the background branches all sites are undergoing either purifying selection or neutral evolution. Analyses were conducted for five foreground branch sets based on initial hypotheses: (1) Cetacea and Sirenia (convergence), (2) Cetacea, (3) Mysticeti, (4) Odontoceti, and (5) Sirenia. For comparison, the model was applied to terrestrial counterparts: (1) Artiodactyla (excluding Cetacea), (2) Tethytheria (excluding Sirenia), and (3) terrestrial Artiodactyla and Tethytheria (convergence). This approach tested whether adaptive changes in sensory genes occurred during aquatic transitions (convergence) or independently within each lineage. Positively selected sites (PSS) were identified using the empirical Bayes method (BEB) (Yang et al. 2005) with posterior probabilities > 0.95. Branch-site model A was compared against a null model of neutral evolution, which is identical to model A except that episodic positive selection in the foreground branches is no longer allowed (Zhang et al. 2005). Similarly, these models were compared by a LRT with one degree of freedom. Alignments were manually inspected to rule out false positives by confirming that the sites were not located in poorly aligned or ambiguous regions and that they exhibited identical amino acid changes exclusive to the target lineages.

#### Hyphy Selection Analyses

We also applied aBSREL, RELAX, and Contrast-FEL (Wertheim et al. 2015; Smith et al. 2015; Kosakovsky Pond et al. 2021) in HyPhy using unrooted species trees. aBSREL estimates the proportion of sites under positive selection across branches while accounting for branch size. We first ran it without specifying foreground branches, then explicitly labeled Cetacea, Mysticeti, Odontoceti, Sirenia, and their terrestrial counterparts for comparison. Statistical significance was assessed by the corrected *p*-values implemented by HyPhy, and branches with values ≤ 0.05 were considered statistically significant. As RELAX does not directly detect positive selection, we applied it to test for shifts in selection intensity between aquatic and terrestrial lineages using the same foreground branches. Intensified selection is indicated by K > 1 and relaxed selection by 0 < K < 1 (*p* < 0.05), with significance assessed by LRT against the null model (K = 1). With the same sets of branches, we used Contrast-FEL to identify codon sites under differential selection between groups, providing complementary validation of sites detected by the BSMA. Multiple testing correction was performed using the false discovery rate (FDR) procedure implemented by HyPhy, and codon sites with *q*-values ≤ 0.2 and *p*-values ≤ 0.05 were considered statistically significant.

### Profile Change with One Change (PCOC) Analysis

To further explore molecular convergence in fully aquatic mammals, we applied the Profile Change with One Change (PCOC) method. Implemented within a maximum likelihood framework and using a rooted amino acid species tree, this approach identifies sites showing convergent shifts in amino acid profiles along branches linked to convergent phenotypes, without requiring identical substitutions (Rey et al. 2018). Results were filtered by applying a posterior probability threshold of > 0.8 for the PCOC, OC, and PC outputs.

### Protein Structure, Variations in Amino Acids and Protein Stability

To investigate protein structure, function, and evolutionary history, we retrieved functional and structural annotations from human canonical proteins in UniProt (https://www.uniprot.org/) and mapped them onto unannotated aquatic mammal sequences using Geneious. As no models were available for these taxa in the AlphaFold Database (Varadi et al. 2022), we predicted 3D structures of TRP receptor proteins from aquatic species and their terrestrial relatives (*Bos taurus* or *Bubalus bubalis* for Cetacea; *Elephas maximus* or *Loxodonta africana* for Sirenia) using the AlphaFold Server powered by AlphaFold 3 (Abramson et al. 2024). To enable structural comparisons, we also retrieved experimentally resolved human protein structures obtained by Cryo-electron microscopy (Cryo-EM), preferentially in apo and/or closed conformational states, from the Protein Data Bank (PDB) (Table S2) as experimental structural references, when available. Structural alignments between the predicted models and their corresponding human reference structures were performed in UCSF ChimeraX 1.9 (Meng et al. 2023). Structural similarity was assessed using the Root Mean Square Deviation (RMSD) and pruned atom pairs between aligned protein structures. Next, we annotated domains and binding sites with UniProt, and mapped PSS onto the models to evaluate the possible structural and functional impacts of PSS mutations.

The potential impact of amino acid substitutions at positively selected sites was assessed with PolyPhen-2 (Adzhubei et al. 2010), via its online interface (http://genetics.bwh.harvard.edu/pph2/), which predicts the effects of nonsynonymous variants on protein structure and function based on sequence, structural, and comparative features, by calculating the naive Bayes posterior probability that a given substitution is damaging. PolyPhen-2 applies two predictive models: HumDiv, optimized for rare high-impact mutations, and HumVar, for common variants with mild effects. It classifies substitutions as benign, possibly damaging, or probably damaging, with scores from 0.0 (benign) to 1.0 (damaging). Substitutions with scores > 0.85 were considered likely to impair function.

Finally, AlphaFold-predicted structures were pre-processed in FoldX 5.0 (Delgado et al. 2019) using the RepairPDB function to correct structural artifacts, after which folding free energy (ΔΔG) was estimated to compare protein thermodynamic stability between aquatic mammals and their terrestrial counterparts.

## Results

### Dataset, Alignments, and Gene Trees

**Fig. 2:**
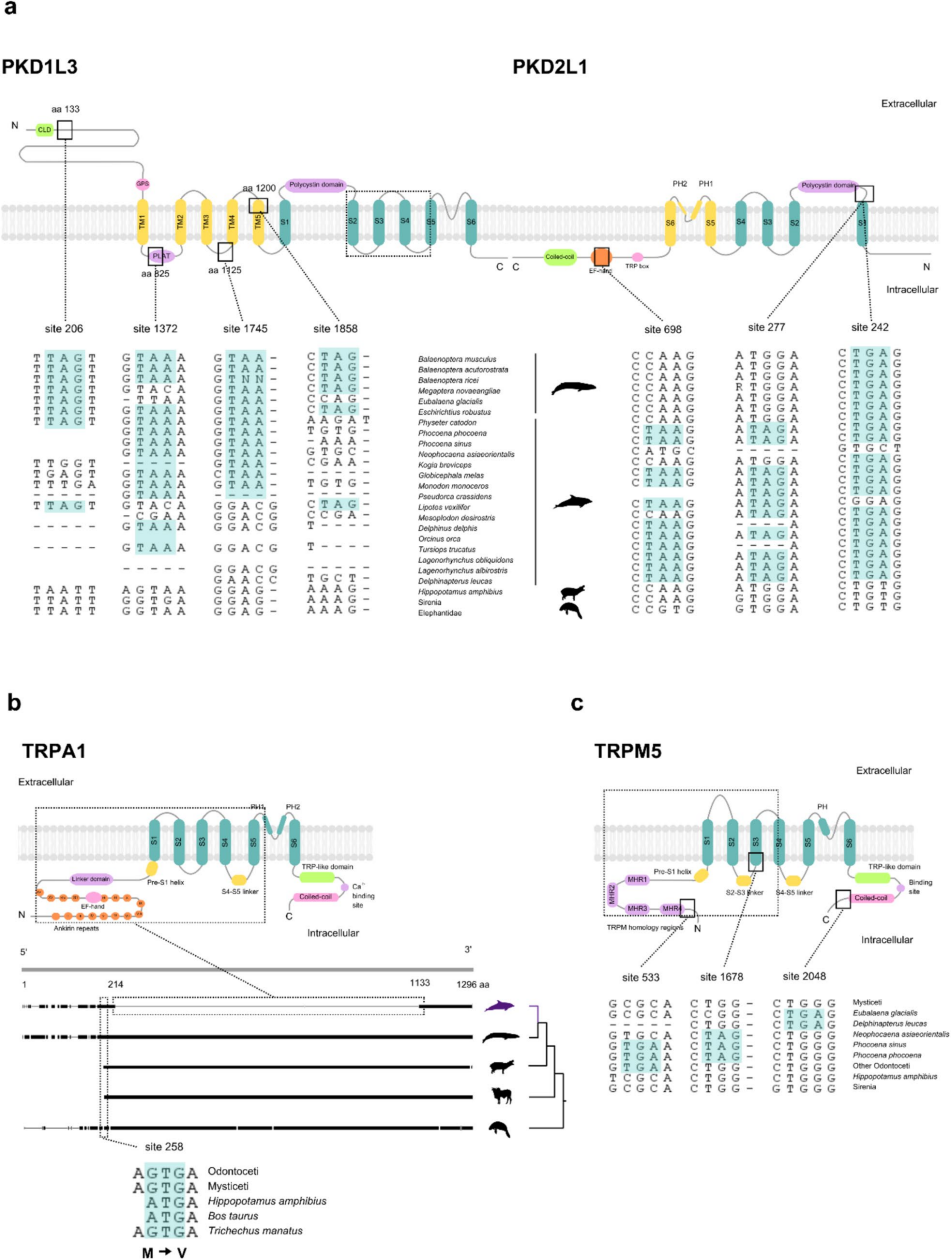
Topological representations of domain architectures in mammalian TRP channels, highlighting regions with inactivating mutations in aquatic mammals. Not to scale. Premature stop codons shared among aquatic species are shown as small squares in the protein structure and highlighted in blue in the alignments below. Large dotted rectangles indicate insertions or deletions. Unique, non-shared stop codons are not shown. **a** Four premature stop codons are shown for PKD1L3 and three for PKD2L1. In cetaceans, nearly the entire region corresponding to transmembrane domains S2–S5 of PKD1L3 is missing, highlighted by the large dotted rectangle. Domain classification and nomenclature follow Ishimaru (2015), Delmas et al. (2004), Su et al. (2018), and Li et al. (2003). **b** Partial loss of TRPA1 in all odontocetes (purple). The gray bar represents the full CDS of *B. musculus* for comparison. A region upstream of the canonical start codon in other mammals is exclusive to Cetacea and Sirenia. In Odontoceti, the absence of nucleotides 842–3613 is highlighted (dotted rectangle), with the corresponding missing region indicated in the topology. Site 258 shows a convergent substitution (methionine, ATG to valine, GTG) exclusive to Cetacea and Sirenia. Domain annotations follow Laursen et al. (2015), Meents et al. (2019), and Talavera et al. (2020). **c** TRPM5 shows three premature stop codons and poorly aligned regions with multiple indels in odontocetes, particularly in the N-terminal cytoplasmic domain (dotted rectangle)

We retrieved the longest coding sequence of each gene from 51 species, encompassing 8 terrestrial mammalian orders, 10 cetacean families, and at least one sirenian representative, using both annotated and unannotated genomes. Species representation varied from 31 (TRPA1) to 51 (PKD2L1, TRPM8, TRPV4) per gene (Table S1). For the selection analysis of putative pseudogenes in cetaceans, we excluded sequences showing evidence of pseudogenization from the alignments, resulting in final datasets of 24 species for PKD1L3, 28 for PKD2L1, and 26 for TRPM5 (Table S1). Using BLAST, we recovered CDSs from unannotated genomes, including all three sirenian species and *Megaptera novaeangliae*, except for TRPA1 and TRPM2, which showed incomplete sequences or contained premature stop codons and frameshifts (Table S4). Most alignments displayed conserved regions within functional domains, with PKD2L1, TRPM3, TRPM8, and TRPV4 being the most conserved. PKD1L3 sequences were incomplete or partially missing in several odontocete species. TRPA1 showed long insertions at the 5’ end in all mysticetes, odontocetes, and *Trichechus manatus* (Fig. 2b), with only fragments persisting at both ends in Odontoceti. This insertion observed in mysticetes and sirenians was removed from the alignments used for selection analyses.

Gene trees reconstructed by IQ-Tree 2 and MrBayes largely recovered the most accepted evolutionary history of mammalian groups with high statistical support (bootstrap ≥ 90; posterior probabilities > 0.9) (Figs. S1–S42). All major orders and superorders matched reference phylogenies, with Cetacea and Sirenia correctly grouped in most trees. Exceptions included unusual placements in TRPV1 (*Physeter catodon* and *Kogia breviceps* as sister group to other cetaceans), TRPM2 (*K. breviceps* also as sister group to other cetaceans), TRPM3 (*D. leucas* outside Cetacea), and TRPV2 (*P. catodon* outside Artiodactyla). Long branch lengths in TRPM3, TRPM8, and TRPV3 for *Lagenorhynchus vexillifer* and deep-diving species (*M. densirostris*, *K. breviceps*, *P. catodon*) suggested mutation accumulation. Sirenians also displayed elongated branches across several gene trees.

### Sensory Genes Inactivated in Cetacea

Manual examination revealed four putative pseudogenes in cetaceans: PKD1L3, PKD2L1, TRPA1, and TRPM5, characterized by multiple premature stop codons and extensive indels causing frameshifts and suggesting gene inactivation and potential loss of function (Fig. 2). These genes remained intact in sirenians and terrestrial mammals.

PKD1L3 and PKD2L1 showed evidence of pseudogenization across all cetaceans. PKD1L3 contained several premature stop codons (TGA and TAA) in all mysticetes, and 12 out of 16 odontocetes analyzed, with four of them shared across both lineages. Notably, a stop codon at site 206 was present in all mysticetes, *L. vexillifer*, and *P. catodon*, while another at site 1858 occurred in all mysticetes except *E. glacialis*. A substitution at site 2225 changed a highly conserved arginine to glycine in all cetaceans. Large deletions affected the N-terminal extracellular domain in most odontocetes and nearly the entire S2–S5 transmembrane region in all cetaceans (Fig. 2a). PKD2L1 was more conserved but contained five shared premature stop codons: four in the extracellular domain between the S1 and S2 (positions 242, 277, 281, 310), and one in the C-terminal EF-hand domain (position 698).

TRPM5 showed inactivating mutations in all odontocetes and three mysticetes (*E. glacialis, M. novaeangliae,* and *E. robustus*). Nine odontocetes and all three mysticetes contained premature stop codons, with multiple ones in *K. breviceps* (2), *M. densirostris* (4), *D. leucas* (2), *N. asiaeorientalis* (2), and *P. sinus* (2). Shared stop codons occurred at position 533 (three phocoenids), 1678 (*N. asiaeorientalis*, *P. sinus*, *M. densirostris*), and 2048 (*E. glacialis* and *D. leucas*). Odontocetes also displayed poorly aligned regions and a shared deletion in S6 present in four mysticetes (Fig. 2c).

The TRPA1 gene was absent from odontocete reference genomes. BLAST searches revealed a large missing region (74.2% of exon 4, all exons 5–26, and 30.7% of exon 27), with intact fragments only at both ends (Fig. 2b), suggesting loss of protein coding function in odontocetes. Insertions upstream of the coding region were found in all cetaceans and sirenians.

Synteny was conserved for all four genes, with minor exceptions: in TRPM5, a pseudogene was annotated between TSPAN32 and ASCL2 in the blue whale; in TRPA1, EPM2, and an intergenic ncRNA present in cattle were absent in cetaceans, where the two upstream elements were instead annotated as pseudogenes. (Fig. S43). RELAX analysis showed relaxed selection in odontocetes for TRPA1 (K = 0.79; *p* = 0.00), while TRPM5 exhibited intensified selection across cetaceans (K = 19.14; *p* = 0.00). No relaxation was detected for PKD2L1 or PKD1L3 (Table 3).

### Lineage-Specific Rates of Evolution in Fully Aquatic Mammals

**Fig. 3:**
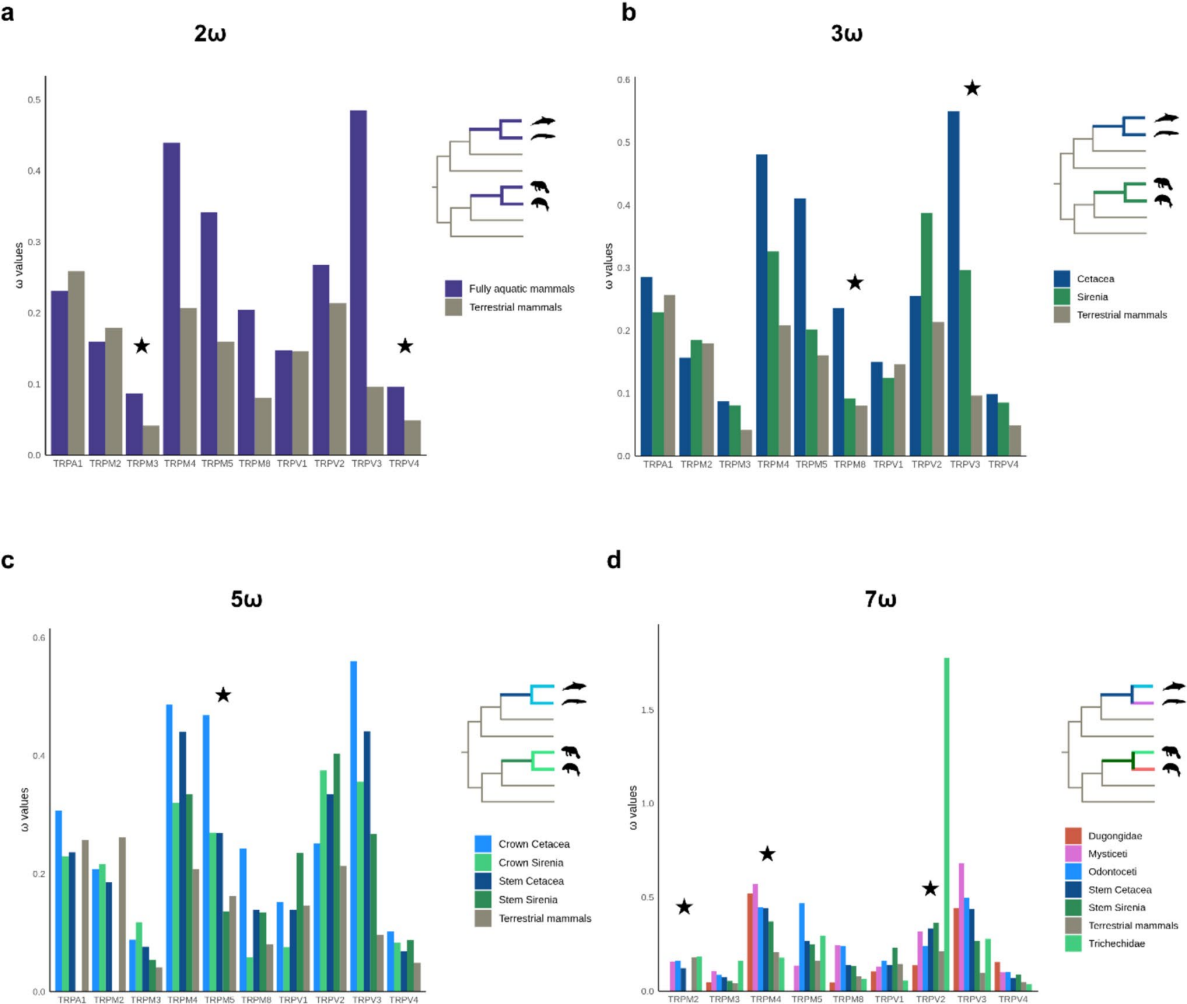
Natural selection patterns and ω values estimated using the branch model in CODEML for TRP genes in aquatic mammal lineages compared to terrestrial mammals. Evolutionary models tested: **a** (2ω) a single ω for Cetacea and Sirenia (convergence). **b** (3ω) separate ω values for Cetacea and Sirenia. **c** (5ω) crown vs. stem lineages of Cetacea and Sirenia. **d** (7ω) separate ω values for stem Cetacea, crown Mysticeti and Odontoceti, stem Sirenia, and sirenian families Trichechidae and Dugongidae. The stars indicate statistically significant models for the tested lineages

Sequences with inactivating mutations were excluded from selection analysis. CODEML branch-model analyses revealed distinct evolutionary patterns among TRP genes, with aquatic mammals (foreground lineages) consistently showing higher ω values than terrestrial mammals (Fig. 3). The 2ω model was the best fit for TRPM3 and TRPV4, with elevated ω values in aquatic lineages (0.087 and 0.096). The 3ω model provided the best fit for TRPM8 and TRPV3, showing increased ω in cetaceans (0.236 and 0.550). The 5ω model was favored for TRPM5, with elevated rates in crown mysticeti (0.469). Finally, the 7ω model was best supported for TRPM2, TRPM4, and TRPV2, with elevated ω in crown Sirenia for TRPM2 (0.185), in Trichechidae for TRPV2 (1.776), and in Mysticeti for TRPM4 (0.571) (Table 1; Fig. 3). TRPA1 and TRPV1 were best explained by the one-ratio model (1ω), indicating a uniform selective regime across lineages (ω = 0.146). Models addressing phylogenetic bias showed similar patterns, with cetaceans displaying the highest overall ω values (Table S7).

**Table 1:** Results of branch model analyses from CODEML for aquatic mammal groups

Gene	Model	*lnL*	LRT	*p *values	ω values
TRPA1	1ω	−22185.64385			0.292
	2ω	−22185.41131	0.47	0.495	0.259 0.231
	3ω	−22185.26086	0.30	0.583	0.257 0.285 0.229
	5ω	−22185.01438	0.79	0.672	0.257 0.236 0.307 0.229
TRPM2	1ω	−35359.0800			0.176
	2ω	−35357.9200	2.3100	0.1300	0.179 0.160
	3ω	−35357.6400	0.5700	0.4500	0.179 0.157 0.185
	5ω	−36882.9970	−3050.7200	−	0.262 0.186 0.208 0.216
	7ω	−35357.1800	3051.6400	**0.0000**	0.179 0.123 0.161 0.158 0.185
TRPM3	1ω	−22665.1100			0.054
	2ω	−22641.3500	47.5100	**0.0000**	0.041 0.087
	3ω	−22641.3100	0.1000	0.7500	0.041 0.087 0.081
	5ω	−22639.9500	2.7000	0.2600	0.041 0.076 0.088 0.054 0.118
	7ω	−22638.4700	2.9700	0.2300	0.041 0.076 0.086 0.108 0.054 0.161 0.047
TRPM4	1ω	−26455.5900			0.243
	2ω	−26412.8100	85.5600	**0.0000**	0.207 0.439
	3ω	−26410.2100	5.2100	**0.0200**	0.208 0.481 0.326
	5ω	−26410.1200	0.1700	0.9200	0.208 0.441 0.486 0.335 0.320
	7ω	−26406.4000	7.4500	**0.0200**	0.208 0.441 0.447 0.571 0.371 0.178 0.521
TRPM5	1ω	−23527.6700			0.182
	2ω	−23495.1900	64.9500	**0.0000**	0.159 0.342
	3ω	−23488.5600	13.2500	**0.0000**	0.161 0.410 0.202
	5ω	−23483.6700	9.7900	**0.0100**	0.162 0.269 0.469 0.136 0.269
	7ω	−23483.6000	0.1300	0.7100	0.162 0.269 0.469 0.136 0.248 0.296
TRPM8	1ω	−22202.4700			0.099
	2ω	−22155.6300	93.6900	**0.0000**	0.080 0.204
	3ω	−22147.2000	16.8500	**0.0000**	0.081 0.236 0.092
	5ω	−22144.8400	4.7300	0.0900	0.081 0.139 0.243 0.134 0.058
	7ω	−22144.7600	0.1500	0.9300	0.081 0.139 0.241 0.246 0.134 0.064 0.046
TRPV1	1ω	−15280.7500			0.146
	2ω	−15280.7500	0.0100	0.9400	0.146 0.147
	3ω	−15280.6300	0.2500	0.6200	0.146 0.150 0.124
	5ω	−15279.5800	2.0900	0.3500	0.146 0.139 0.152 0.236 0.075
	7ω	−15279.1800	0.8000	0.6700	0.146 0.139 0.161 0.131 0.232 0.057 0.105
TRPV2	1ω	−20886.2600			0.226
	2ω	−20882.6200	7.2800	**0.0100**	0.214 0.267
	3ω	−20880.9200	3.4000	0.0700	0.213 0.255 0.388
	5ω	−20880.5900	0.6600	0.7200	0.213 0.335 0.251 0.403 0.375
	7ω	−20873.5200	14.1300	**0.0000**	0.213 0.334 0.241 0.318 0.365 1.776 0.138
TRPV3	1ω	−17349.8500			0.161
	2ω	−17200.9600	297.7800	**0.0000**	0.096 0.485
	3ω	−17195.8400	10.2300	**0.0000**	0.096 0.550 0.296
	5ω	−17195.2600	1.1600	0.5600	0.096 0.441 0.561 0.267 0.356
	7ω	−17193.7000	3.1200	0.2100	0.096 0.439 0.498 0.680 0.268 0.279 0.443
TRPV4	1ω	−15304.8600			0.057
	2ω	−15290.0100	29.7100	**0.0000**	0.049 0.096
	3ω	−15289.8500	0.3200	0.5700	0.049 0.099 0.085
	5ω	−15289.3800	0.9400	0.6200	0.049 0.069 0.102 0.088 0.083
	7ω	−15287.9300	2.9000	0.2300	0.049 0.069 0.103 0.100 0.087 0.037 0.155

LRT: likelihood ratio test. The first value in the “ω values” column always corresponds to the background branches. Subsequent values represent the foreground branches for each model: 1ω, null model (one ratio); 2ω, fully aquatic mammals; 3ω, separate ω values for Cetacea and Sirenia; 5ω, crown and stem lineages of Cetacea and Sirenia; and 7ω, separate ω values for stem Cetacea, crown Mysticeti and Odontoceti, stem Sirenia, and sirenian families Trichechidae and Dugongidae. Bolded *p*-values indicate statistically significant models for the tested lineages

### Diversifying Selection in Aquatic Mammals

**Fig. 4:**
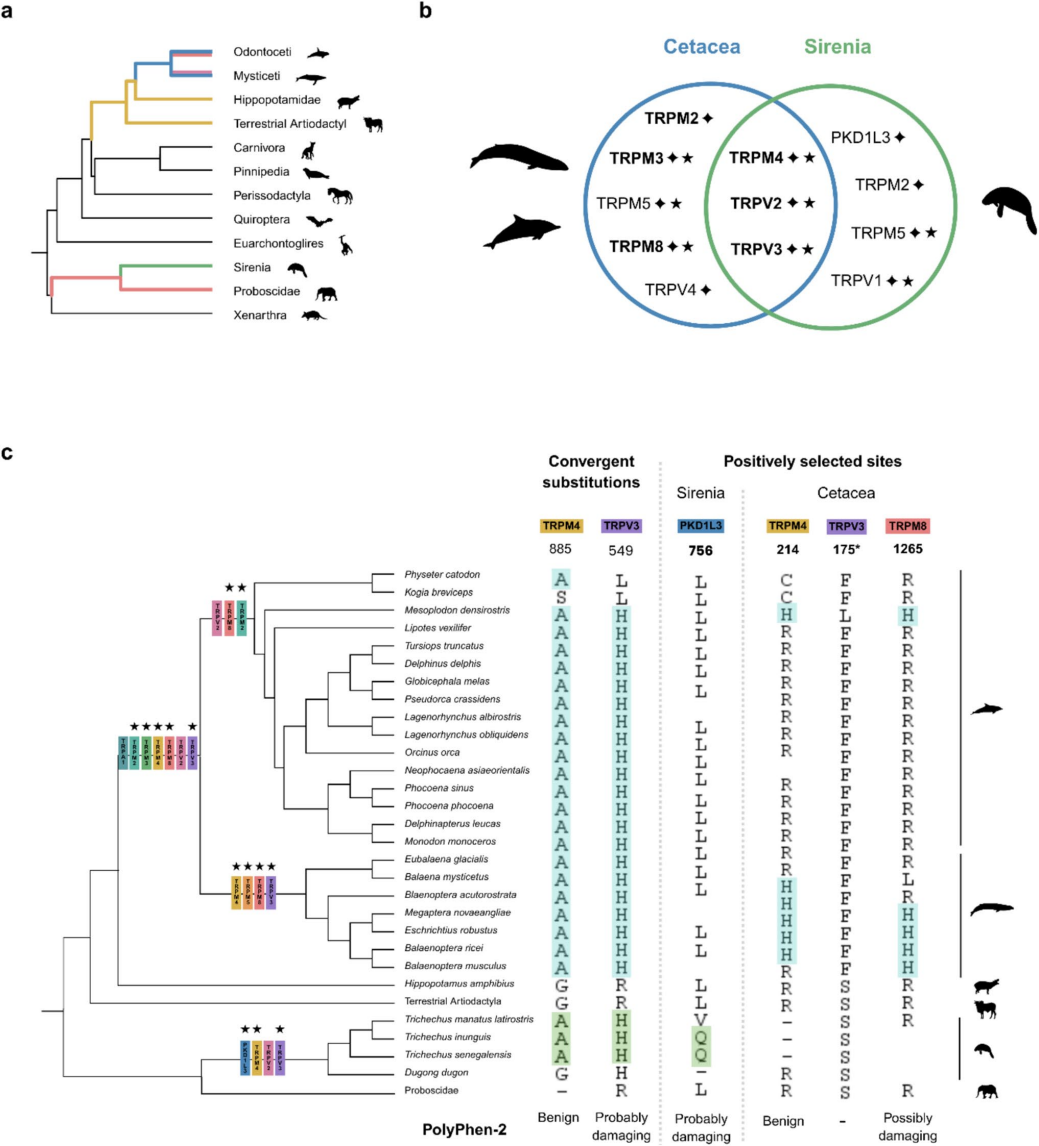
Genes and lineages showing signatures of episodic diversifying selection based on CODEML and HyPhy analyses. **a** Schematic representation of foreground branch sets used in selection tests, based on evolutionary hypotheses: Cetacea and Sirenia (convergent selection); Cetacea (blue); Mysticeti (purple); Odontoceti (red); and Sirenia (green). Terrestrial counterparts comparison: Artiodactyla (yellow); Tethytheria (pink); and terrestrial Artiodactyla + Tethytheria (convergent selection). **b** Venn diagram showing genes under positive selection in cetaceans (blue) and sirenians (green), with shared genes in the intersection. Genes in **bold:** supported by at least three methods (CODEML branch-site model, RELAX, and aBSREL). A five-pointed star (★): convergent amino acid shifts detected by PCOC. A four-pointed star (✦): branches with PSSs identified by Contrast-FEL. All genes shown in the intersection were also identified as positively selected when cetaceans and sirenians were tested separately, and were supported under the convergence model in CODEML and by PCOC, with the exception of TRPA1. **c.** Results from the CODEML branch-site model and aBSREL (*p* < 0.05) for aquatic mammal lineages. Colored boxes mark genes under positive selection. A five-pointed star (★): genes with at least one branch under selection in aBSREL. On the right, amino acid alignments for each gene display substitutions across species. Residues in blue correspond to cetaceans, and in green to sirenians. Blank positions indicate missing data. Sites in **bold** were also identified as positively selected by Contrast-FEL. Asterisks (*): sites with signals of molecular convergence detected by PCOC. Below each alignment, PolyPhen-2 predictions (“Benign,” “Possibly damaging,” or “Probably damaging”) are shown for each substitution

Branch-site model analyses in CODEML detected positive selection (ω > 1; *p* < 0.05) across multiple TRP genes and lineages (Table 2; Fig. 4). Significant signals were found for: (1) both cetaceans and sirenians (convergent evolution): TRPM4, TRPV2, TRPV3; (2) all cetaceans: TRPM2, TRPM3, TRPM4, TRPM8, TRPV2, TRPV3; (3) mysticetes: TRPM4, TRPM5, TRPM8, TRPV3; (4) odontocetes: TRPM2, TRPM8, TRPV2; and (5) sirenians: PKD1L3, TRPM4, TRPV2, TRPV3. No positive selection was detected for TRPA1, PKD2L1, TRPV1, and TRPV4. The TRPV3 gene showed the highest number of PSS in both Cetacea and Sirenia (58 sites), while TRPM2 showed none. All PSS were manually validated. When terrestrial lineages were tested, the branch-site model identified selection in PKD1L3 and TRPA1 within Tethytheria, but the PKD1L3 PSS did not overlap with those in Sirenia, indicating site-specific differences even among related groups (Table S8).

**Table 2:** Results of branch-site model analyses from CODEML for aquatic mammal groups and a test of convergence (Cetacea and Sirenia)

Gene/foreground branch	LRT	*p* values (< 0.05)	Positively selected sites (BEB > 0.95)
Cetacea			
TRPM2	3.89	0.0484	–
TRPM3	118.00	0.0000	1513, 1644
TRPM4	5.12	0.0236	148, 214, 246, 458, 556, 827, 991, 1004, 1204
TRPM8	5.10	0.0239	69, 407, 500, 557, 576, 1138, 1265
TRPV2	12.50	0.0004	208, 305, 491, 581, 712, 719, 753, 757, 809
TRPV3	18.15	0.0000	34, 37, 52, 130, **175**, 177, 178, 188, 193, 197, 201, 228, 247, 275, 370, 414, 427, 549, 550, 551, 557, 558, 559, 560, 561, 562, 564, 566, 567, 574, 636, 691, 711, 716, 720, 735, 746, 792, 801, 805, 866, 882, 944, 958
Mysticeti			
TRPM4	5.30	0.0000	458
TRPM5	14.00	0.0002	187, 683, 716, 745
TRPM8	14.68	0.0001	568, 407, 1265
TRPV3	90.37	0.0000	28, 29, 178, 805, 849, 850, 851, 853, 866, 873, 875, 876, 877, 878, 879, 880, 882, 883, 884, 888, 964, 966, 967
Odontoceti			
TRPM2	15.97	0.0001	–
TRPM8	6.20	0.0128	70, 137, 576, 726, 848, 1138
TRPV2	4.89	0.027	305, 719
Sirenia			
PKD1L3	53.06	0.0000	756, 1320
TRPM4	13.00	0.0004	–
TRPV2	24.29	0.0000	837, 841
TRPV3	30.92	0.0000	40, **393**, **436**, **438**, 537, 959
Cetacea and Sirenia (convergence)			
TRPM4	4.91	0.0000	885
TRPV2	10.07	0.0015	–
TRPV3	4.34	0.0372	48, 169, **175**, 227,247, 294, 378, 380, 381, **393**, 395, 414, 415, **436**, 437, 549, 551, 559, 560, 561, 711

LRT: likelihood ratio test. Positively selected sites (BEB > 0.95): codons identified by the Bayes Empirical Bayes (BEB) method with posterior probabilities greater than 0.95. Only statistically significant results (*p* < 0.05) are shown. Bolded sites indicate convergent substitutions detected by both the branch-site model in CODEML and PCOC

Contrast-FEL detected divergent selective pressures in 10 TRP genes (except PKD2L1 and TRPA1), with TRPV3 exhibiting the highest number of PSSs, followed by TRPM5 (Table 3). Several sites were supported by both the branch-site model in CODEML and Contrast-FEL, including PKD1L3 (2 sites), TRPM4 (8), TRPM5 (4), TRPM8 (9), TRPV2 (11), and TRPV3 (78) (Tables 2–3; Fig. 4).

**Table 3:** Results of Hyphy evolutionary analyses (RELAX, aBSREL, and Contrast-FEL) for aquatic mammal groups

	RELAX				aBSREL		Contrast-FEL
Branch/gene	Selection strength	K	*p* value	LR	Branches under episodic diversifying selection	Adjusted *p* value	Sites (*q* ≤ 0.2; *p* ≤ 0.05)
Cetacea							
TRPM2	Intensified	1.80	0.000	23.74	*P. sinus*	0.00	
TRPM3	Intensified	5.07	0.000	34.84	*T. truncatus*	0.00	453, 477
					*E. glacialis*	0.021	
TRPM4	Relaxed	0.39	0.000	56.03	Node 41	0.048	**214, 246, 556, 1004**, 320, 913, **1204**, 1155
TRPM5*	Intensified	19.14	0.00	143.33			
TRPM8	Relaxed	0.57	0.00	59.18	*B. mysticetus*	0.00	64, 83, **576**, 681,
					Node 10	0.009	830, **1138, 1265**, 1190, 61
TRPV1	Relaxed	0.43	0.00	12.60			833
TRPV2	Relaxed	0.66	0.00	13.76			**208**, **491**, **581**, **809**, **305, 712, 719, 753, 757**
TRPV3	Relaxed	0.35	0.00	164.15	*B. mysticetus*	0.00	**34, 130, 178, 550, 557, 558, 562**, 808, **866**, 874, **879**, **881**, **882**, **884**, **958**, 964, 850, **877**, **888**, **275**, **966**, **37, 52, 188, 197, 228, 285, 559, 561, 564, 566, 567, 574, 716, 735, 746, 792, 801**, **849**, **851**, 871, **878**, **883**, **944, 175, 177, 560, 805**, **853**, **873**, 957, **370, 636, 691**, 544, 229, 525, **201**, 212
					*B. acutorostrata *	0.00	
					*M. novaeangliae*	0.029	
TRPV4					*L. vexillifer*	0.00	649
					*B. mysticetus*	0.004	
Mysticeti							
TRPM2					*B. mysticetus*	0.046	
TRPM3					*E. glacialis*	0.007	
TRPM4	Relaxed	0.27	0.000	17.18	Node 41	0.015	**458**
TRPM5					*B. ricei*	0.00	177, **187**, 217, 720, 742, 743, 807, 1005, 1108, 1120, 1122, 1123, 1155, 1201, 68, 100, 394, **683**, 715, **716**, 746, 830, 854, 885, 887, 931, 956, 1009, 1061, 1062, 1111, 1121, 1156, 924, 718, 1110, 148, 253, 488, 740, **745**
					Node 9	0.00	
					*B. acutorostrata *	0.00	
					*B. mysticetus*	0.00	
					*B. musculus*	0.002	
					Node 7	0.003	
TRPM8	Intensified	1.87	0.001	11.25	*B. mysticetus*	0.00	**407**, **1265**, 1269, 1261, 1272, 1281, 1282
TRPV2							21, 32
TRPV3	Intensified	3.04	0.00	89.01	*B. mysticetus*	0.00	**805, 849, 850, 851, 853, 866**, 871, **873**, 874, **875, 876, 877, 878, 879, 880, 881, 882, 883, 884**, 885, **888**, 957, **964, 966, 967**, **958**, 865, **178**, 887, 37
					*B. acutorostrata *	0.00	
					*M. novaeangliae*	0.007	
					Node 40	0.027	
TRPV4					*B. mysticetus*	0.002	650
Odontoceti							
TRPM2	Intensified	1.98	0.000	26.86	*P. sinus*	0.00	1506, 1516
TRPM3	Intensified	4.77	0.000	30.77	*T. truncatus*	0.00	453, 477, 762, 419, 452, 454, 755, 760, 762, 1622
TRPM4	Relaxed	0.39	0.000	17.44			**827**, 913, 1155,** 991**
TRPM8	Relaxed	0.53	0.00	57.98	Node 10	0.006	28, 64, 83, **576, 1138,** 70, **137**, **848**
					*P. catodon*	0.01	
TRPV1	Relaxed	0.60	0.006	7.43			16, 833
TRPV3	Relaxed	0.28	0.00	129.66			34, 130, **557**, **558**, **561**, **562**, **228**, 229, **559**, **560**, **566**, **567**, **735**, **746**, 808, 525
TRPV4					*L. vexillifer*	0.00	506, 510, 513
Sirenia							
PKD1L3					*T. manatus *	0.00	754, **756**, 775, 782, 783, 785, 786, 787, 791, 797, 799, 1168, *1319*, **1320**, 1697, 1742, 2186
					Node 37	0.002	
PKD2L1	Relaxed	0.66	0.036	4.38			
TRPM2							818, 1302
TRPM3	Relaxed	0.72	0.040	4.23			
TRPM4					*D. dugon*	0.00	284, 392, 398, 400, 401, 405, 447, 401, 550, 619
					*T. manatus*	0.00	
TRPM5	Relaxed	0.79	0.015	5.96			221, 231, 236
TRPV1	Intensified	3.98	0.00	42.61			
TRPV2							**837**, **841**
TRPV3					Node 86	0.00	**294**, **378**, **380**, **381**, **393**, 395, **436**, *437*, **438**, **537**, 415, **959**, **40**
TRPV4	Relaxed	0.39	0.004	8.37			

Under RELAX, selection strength indicates whether selection is relaxed or intensified; K: selection intensity parameter; LR: likelihood ratio; *q*-value: FDR-corrected *p*-value reported by Contrast-FEL. Under aBSREL, each branch under episodic diversifying selection is associated with a corresponding *p*-value. For Contrast-FEL, positively selected sites are shown for each branch. Only statistically significant results (*q* ≤ 0.05 and *p* ≤ 0.05) are reported for each analysis. Bolded sites were identified by both Contrast-FEL and CODEML. Italicized sites are shared between aquatic mammals and their terrestrial counterparts. An asterisk indicates the TRPM5 alignment containing cetacean sequences with inactivating mutations

RELAX analyses revealed both relaxed and intensified selection across TRP genes in aquatic mammals (Table 3). Cetaceans showed relaxed selection in TRPM4, TRPM8, TRPV1, TRPV2, and TRPV3, but intensified selection in TRPM2 and TRPM3. Mysticetes exhibited relaxed selection in TRPM4 and intensified selection in TRPM8 and TRPV3. In odontocetes, TRPM4, TRPM8, TRPV1, and TRPV3 were under relaxed selection, whereas TRPM2 and TRPM3 were under intensified pressure. Sirenians showed relaxed selection in PKD2L1, TRPM3, TRPM5, and TRPV4, and intensified selection in TRPV1. Overall, cetaceans and sirenians displayed more genes under relaxed selective pressure than terrestrial mammals (K < 1; *p* < 0.05). Episodic diversifying selection (aBSREL) was detected in 8 genes in cetaceans (except TRPV1 and TRPV2) and in PKD1L3, TRPM4, and TRPV3 in sirenians, with signals largely restricted to aquatic lineages (Table 3; Fig. 4).

### Site-Level Convergent Evolution Between Cetacea and Sirenia

Both branch-site models and PCOC detected positively selected sites between cetaceans and sirenians. The BSMA identified significant selection in TRPM4, TRPV2, and TRPV3, while PCOC recovered high-confidence convergent sites (posterior probability > 0.8) in seven genes: TRPM3 (1), TRPM5 (2), TRPM8 (2), TRPV1 (2), TRPV2 (1), TRPM4 (6), and TRPV3 (6) (Fig. 4; Tables 2, S9). After manual curation, the BSM retained one site in TRPM4, and 22 in TRPV3. Notably, four TRPV3 sites (175, 393, 436, and 438) were consistently supported by both methods.

### Variations in positively Selected Sites Potentially Affect Protein Structure and Stability

PolyPhen-2 analyses indicated that 47 of the 68 PSS in PKD1L3, TRPM4, TRPM8, TRPV2, and TRPV3 were “benign,” while 29 sites were “probably damaging”, and 10 “possibly damaging.” Comparison with the UniProt database revealed that 48 substitutions had no known human variants, and 19 were not associated with human diseases. However, 16 overlapped with somatic mutations or variants linked to inherited disorders. In TRPM4, substitutions at sites 214 (Arg → His), 246 (Gly → Ser), and 1004 (Pro → Leu) were associated with Progressive familial heart block type IB, with site 214 also linked to hypertrophic cardiomyopathy. In TRPV3, substitutions at sites 294 (Glu → Lys), present in both cetaceans and sirenians, and 720 (Ala → Thr), in cetaceans, were associated with palmoplantar keratoderma (Table 4).

**Table 4:** Amino acid substitutions at PSSs in aquatic mammals and their potential impact in human protein as assessed by PolyPhen-2

Gene	Uniprot ID	PSS	Human protein position	Human → aquatic mammal amino acid	PolyPhen-2 HumDiv score	PolyPhen-2 classification	Uniprot category
PKD1L3	Q7Z443 PK1L3_HUMAN	756	469	L → Q	0.973	Probably damaging	No disease associated
TRPM4	Q8TD43 TRPM4_HUMAN	148	148	S → N	0.999	Probably damaging	Variant assessed as somatic; high impact
		214	214	R → H	0.032	Benign	Progressive familial heart block type IB
							Hypertrophic cardiomyopathy
							Likely benign
		246	241	G → S	0.999	Probably damaging	Variant assessed as somatic; moderate impact
							Progressive familial heart block type IB
		458	453	H → R	0.042	Benign	Variation not present in humans
				H → C	0.996	Probably damaging	Variation not present in humans
		556	540	L → P	0.999	Probably damaging	Variation not present in humans
		991	947	A → T	0.966	Probably damaging	Variation not present in humans
		1004	960	P → S	0.022	Benign	Variation not present in humans
				P → A	0.022	Benign	Variation not present in humans
				P → L	0.002	Benign	Progressive familial heart block type IB
		1204	1160	K → Q	0.999	Probably damaging	Variation not present in humans
		885	844	G → A	0.002	Benign	Variation not present in humans
TRPM5	Q9NZQ8 TRPM5_HUMAN	187	187	E → D	0.002	Benign	Variation not present in humans
TRPM8	Q7Z2W7 TRPM8_HUMAN	69	14	R → G	0.900	Possibly damaging	Variation not present in humans
				R → Q	0.973	Probably damaging	Variation not present in humans
		407	224	D → E	0.005	Benign	Variation not present in humans
		500	317	N → S	0.084	Benign	No disease associated
		557	374	T → I	0.000	Benign	Variant of uncertain significance
		576	393	V → I	0.994	Probably damaging	Variation not present in humans
		1138	955	I → V	0.000	Benign	Variation not present in humans
		1265	1081	R → H	0.787	Possibly damaging	Variant assessed as somatic; high impact
		568	385	L → H	0.998	Probably damaging	Variant assessed as Somatic; moderate impact
		137	25	Y → C	0.000	Benign	Variation not present in humans
				Y → H	0.145	Benign	Variation not present in humans
TRPV2	Q9Y5S1 TRPV2_HUMAN	208	162	R → Q	0.940	Possibly damaging	Variant assessed as Somatic; moderate impact
		305	259	N → D	0.010	Benign	Variation not present in humans
		491	441	L → V	0.035	Benign	Variation not present in humans
		581	508	L → V	0.003	Benign	No disease associated
		712	605	I → V	0.945	Possibly damaging	Variation not present in humans
		719	612	I → M	1.000	Probably damaging	Variation not present in humans
		753	646	F → V	0.991	Probably damaging	Variation not present in humans
		757	650	S → N	0.000	Benign	Variation not present in humans
		809	697	N → S	0.019	Benign	Variation not present in humans
		841	711	S → R	0.034	Benign	Variation not present in humans
		837	707	S → L	0.016	Benign	Variation not present in humans
				W → R	1.000	Probably damaging	Variation not present in humans
				E → S	0.577	Possibly damaging	Variation not present in humans
TRPV3	Q8NET8 TRPV3_HUMAN	34	5	P → L	0.023	Benign	Variation not present in humans
		37	8	M → T	0.000	Benign	No disease associated
				M → I	0.000	Benign	No disease associated
		52	23	P → V	0.191	Benign	Variation not present in humans
				P → A	0.0100	Benign	Variation not present in humans
		130	54	P → S	0.043	Benign	No disease associated
				P → T	0.180	Benign	Variation not present in humans
		178	101	N → S	0.001	Benign	Variant assessed as somatic; moderate impact
		188	109	K → Q	0.000	Benign	Variation not present in humans
		197	118	R → W	0.930	Possibly damaging	Variant of uncertain significance
		201	122	R → H	0.346	Benign	No disease associated
		228	149	R → C	0.597	Possibly damaging	No disease associated
		275	188	R → Q	0.896	Possibly damaging	Variant assessed as somatic; moderate impact
		370	283	E → G	0.977	Probably damaging	Variation not present in humans
				E → D	0.001	Benign	Variation not present in humans
		427	340	D → N	0.000	Benign	No disease associated
		550	463	P → S	1.000	Probably damaging	Variation not present in humans
				P → T	0.998	Probably damaging	Variation not present in humans
				P → A	0.998	Probably damaging	No disease associated
		557	470	P → K	1.000	Probably damaging	Variation not present in humans
				P → S	1.000	Probably damaging	Variation not present in humans
				P → T	0.996	Probably damaging	Variation not present in humans
		564	477	H → Y	0.001	Benign	No disease associated
				H → N	0.76	Possibly damaging	Variation not present in humans
		567	480	G → W	0.975	Probably damaging	No disease associated
		636	544	Y → C	1.000	Probably damaging	No disease associated
		691	599	L → F	0.998	Probably damaging	Variation not present in humans
		720	628	A → T	0.001	Benign	TRPV3-related disorder
							Isolated focal non-epidermolytic palmoplantar keratoderma
							Benign
		735	643	N → K	0.000	Benign	Variation not present in humans
		746	654	F → S	0.815	Possibly damaging	Variation not present in humans
		866	759	T → S	0.006	Benign	Variation not present in humans
		944	781	E → D	0.996	Probably damaging	Variation not present in humans
		958	785	F → V	0.000	Benign	Variation not present in humans
		48	19	P → S	0.000	Benign	No disease associated
		227	148	R → Q	0.999	Probably damaging	No disease associated
		247	160	H → R	0.046	Benign	Variation not present in humans
		294	207	E → K	0.002	Benign	Variation not present in humans
							Variant assessed as somatic; moderate impact
							Isolated focal non-epidermolytic palmoplantar keratoderma
							Variant of uncertain significance
		414	327	R → K	0.000	Benign	Variation not present in humans
		549	462	R → H	1.000	Probably damaging	Variant assessed as somatic; moderate impact
		561	474	A → S	0.001	Benign	No disease associated
		711	619	C → S	0.965	Probably damaging	Variation not present in humans
		178	101	N → S	0.001	Benign	Variant assessed as somatic; moderate impact
		866	759	T → S	0.006	Benign	Variation not present in humans
		40	11	L → A	0.995	Probably damaging	Variation not present in humans
		537	450	F → I	0.003	Benign	Variation not present in humans
		959	786	P → I	0.974	Probably damaging	Variation not present in humans

PSSs identified in TRP channel genes of aquatic mammals, with corresponding positions in the human protein. The table shows the amino acid substitutions, the PolyPhen-2 HumDiv scores and predicted impact (“Benign,” “Probably damaging,” or “Possibly damaging”), and UniProt annotations regarding potential clinical relevance based on known human variants

Structural modeling revealed conservation of the overall protein fold across terrestrial and aquatic mammals despite sequence divergence (Table S10; Fig. S44–S52). Furthermore, structural alignments between AlphaFold3-predicted TRP proteins and the corresponding human Cryo-EM structures showed overall agreement, particularly in the transmembrane and coiled-coil regions, supporting the consistency of AlphaFold predictions with experimentally resolved protein structures (Table S10; Fig. S53–S59). RMSD values indicate structural similarity despite methodological differences between predicted and experimentally determined models.

**Fig. 5:**
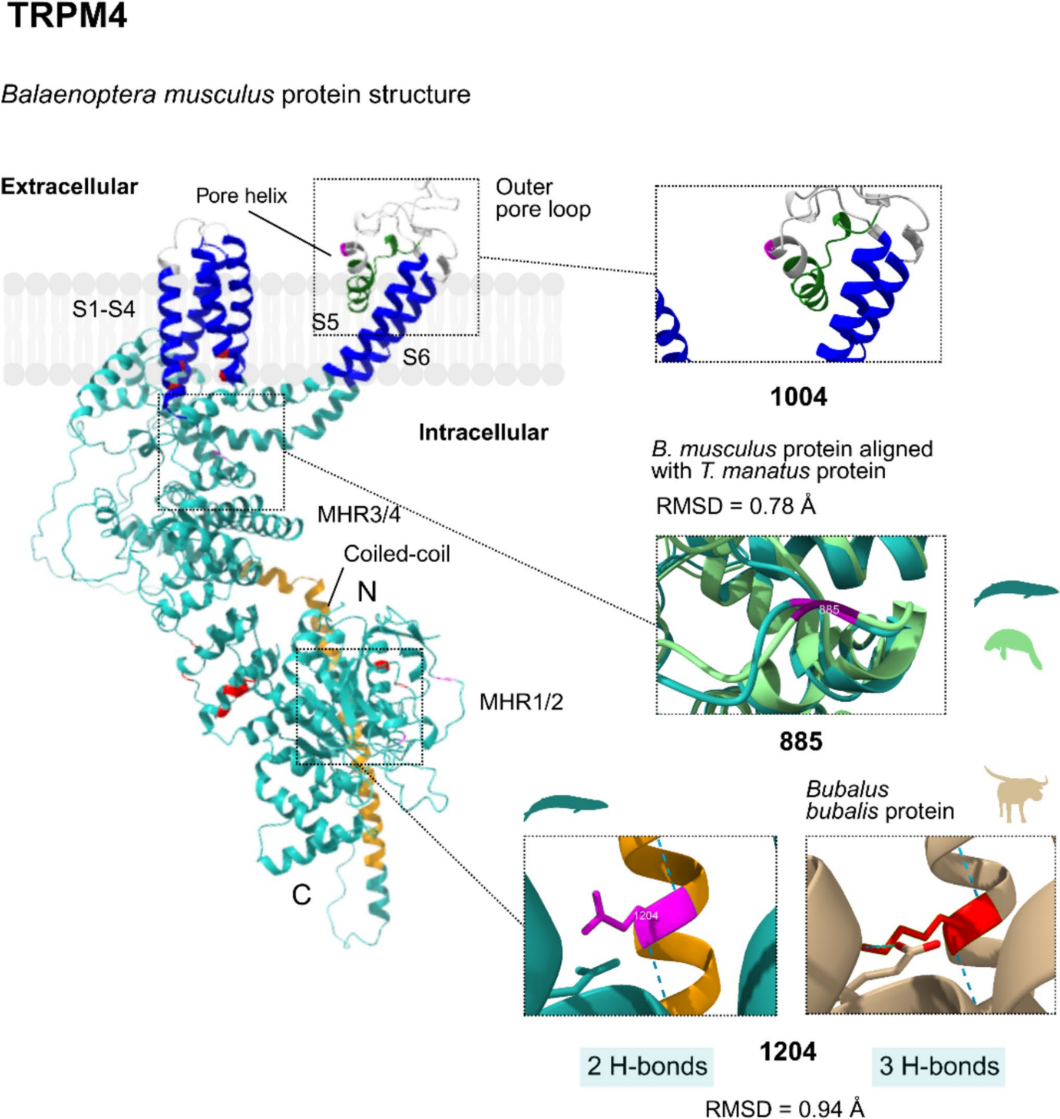
Ribbon diagram of a single subunit of the *B. musculus* TRPM4 protein structure predicted by AlphaFold3. Domains, binding sites, and PSS are color-coded: light blue, cytoplasmic domains (N- and C-termini); dark blue, transmembrane domains (S1–S6); gray, extracellular loops; dark green, pore helix; orange, coiled-coil domain; red, active binding sites; purple, PSS in Cetacea; light green, PSS in Sirenia; yellow, corresponding site in *Bos taurus*. Light blue dashed lines indicate hydrogen bonds. Numbers in bold correspond to PSSs. Sites are highlighted and shown in close-up views for better visualization and comparison. Annotations were retrieved from Uniprot and based on Huang et al. (2020)

**Fig. 6:**
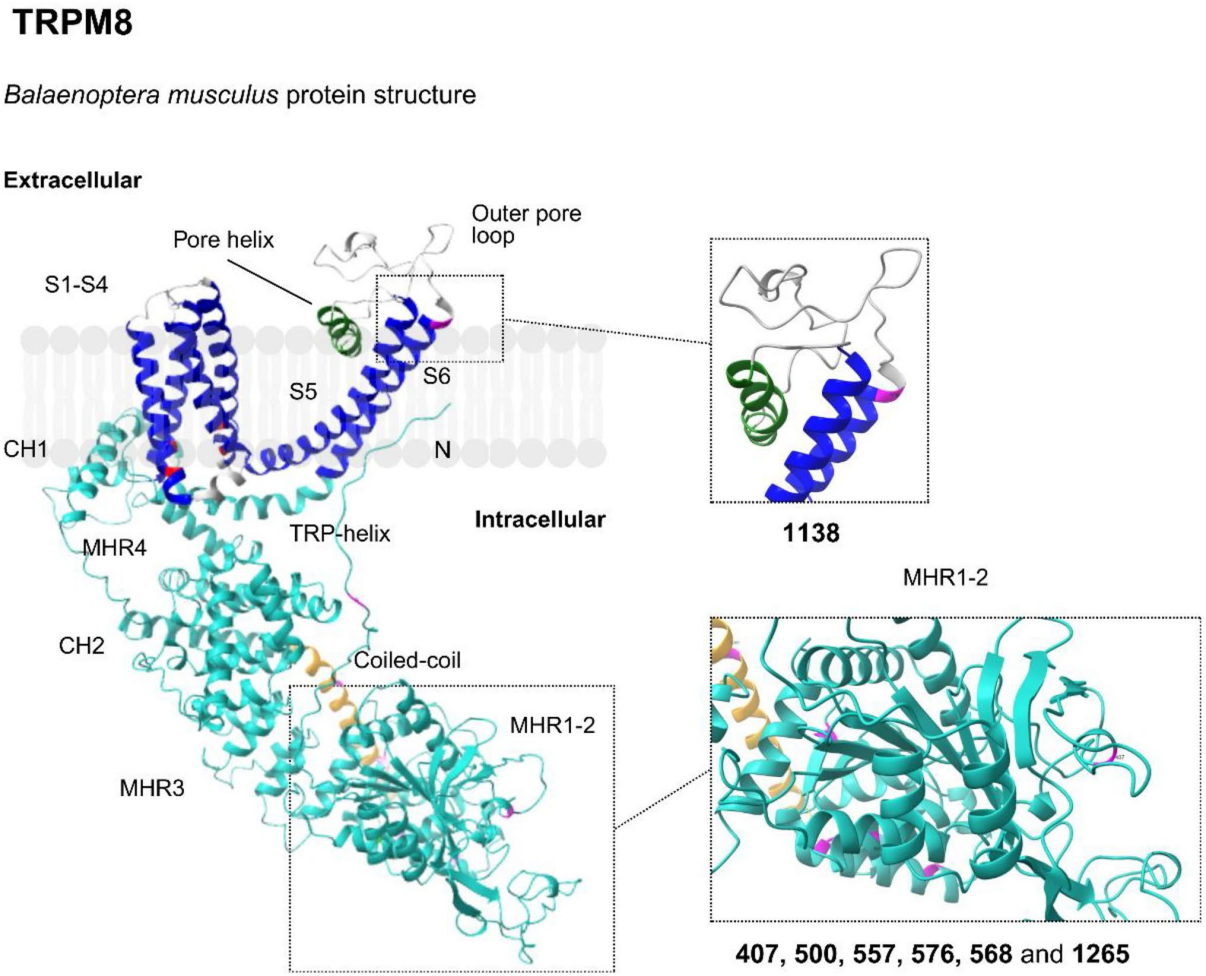
Ribbon diagrams of a single subunit of the *T. truncatus* TRPV2 protein structure predicted by AlphaFold3. Positively selected site 305 (purple) is highlighted and shown in a close-up view on the right. Electrostatic surface potential is mapped onto the protein surface: red, negative; blue, positive; white, neutral

Most PSS did not induce major conformational changes in aquatic mammals’ proteins compared to terrestrial homologs. In TRPM4, eight sites were mapped to functionally relevant regions, including site 1004 in the extracellular domain (slight conformational shift), site 214 in an N-terminal binding site (minimal displacement), site 1204 in the coiled-coil domain (loss of one hydrogen bond), and convergent site 885 in the S2-S3 cytoplasmic loop (structurally conserved) (Fig. 5). TRPM8 showed overall conservation except for N-terminal and C-terminal domain translocations in dolphins (Fig. S49). In TRPV2, a non-conservative substitution (N → D) at site 305 introduced a negative charge, potentially disrupting local interactions (Fig. 6).

Protein stability analysis revealed a general trend of reduced stability (increased ΔΔG) in aquatic mammals relative to terrestrial species, particularly in *Trichechus manatus* (PKD1L3, TRPV2, TRPV3), *Balaenoptera musculus* (TRPM4, TRPM8), and *Orcinus orca* (TRPV2). Increased stability was predicted for TRPM2, TRPM3, and TRPM8 in odontocetes, and TRPV3 in *B. musculus* (Table S11).

## Discussion

Cetaceans and sirenians represent two distinct mammalian lineages that have fully transitioned to an aquatic lifestyle, resulting in sensory systems extensively remodeled by the challenges of this environment (Nery et al. 2014; McGowen et al. 2014). TRP receptors, which are central to chemical and somatosensory signal transduction, provide a compelling framework for investigating the molecular basis of these adaptations (Hoffstaetter et al. 2018). Our results reveal evidence of molecular adaptation in TRP genes across these lineages, characterized by accelerated evolutionary rates and lineage-specific molecular signatures. We identified multiple lineage-specific positively selected sites, along with convergent amino acid substitutions in functionally important protein domains, highlighting both shared and independent evolutionary trajectories. Furthermore, the detection of potential pseudogenization in certain cetacean TRP genes suggests a secondary loss of ancestral sensory pathways during the transition to water. Together, these findings highlight how the interplay between aquatic colonization, specialized diets, and diverse habitats has driven the molecular diversification of sensory pathways in fully aquatic mammals.

### Convergent Evolution of Sensory Genes Underlies Adaptation to Fully Aquatic Life

Cetaceans and sirenians exhibit distinctive and convergent traits shaped by similar selective pressures associated with life in water. Consistent with broader patterns of convergence among aquatic vertebrates (Berta et al. 2007; McGowen et al. 2014; Yuan et al. 2021), we identified site-level convergence in the heat and mechanosensory receptors TRPM4, TRPV2, and TRPV3 (Fig. 4c). These shared amino acid changes suggest repeated adaptive modifications that may change sensory perception in aquatic environments, underscoring the central role of mechano- and thermoreception in sustaining life underwater.

ThermoTRP channels are key regulators of mammalian homeothermy and, although many of them are highly conserved, others have undergone adaptive shifts in lineages exposed to novel environmental conditions (Lynch et al. 2015; Laursen et al. 2016; Yarmolinsky et al. 2016; Saito and Saito 2025). The transition from land to water exposed aquatic mammals to distinct thermal regimes (Yang et al. 2020; Lu et al. 2023), likely imposing new selective pressures on somatosensory pathways. Within this context, the convergent changes observed in cetaceans and sirenians may reflect independent optimization of TRP channels under combined demands of thermal and mechanical sensing in aquatic habitats (Thewissen and Nummela 2008; Bauer and Reep 2018; Vermeij and Motani 2018).

One such example is TRPM4, which encodes a Ca^2^⁺-activated monovalent cation channel involved in thermosensation, mechanosensation, and taste transduction (Saito and Tominaga, 2015; Dutta Banik et al. 2018; Dutta Banik and Medler, 2023; Nilius and Flockerzi, 2014) (Fig. 1b). At site 885, glycine was independently replaced by alanine in both cetaceans and sirenians (Fig. 4c). Although chemically conservative, this substitution occurs at a highly conserved vertebrate position (Tsuruda et al. 2006) (Fig. 5), suggesting adaptive fine-tuning of channel properties under selective constraints.

A similar evolutionary trajectory is observed in TRPV3. Although Wu et al. (2022) reported TRPV3 inactivation in cetaceans and linked this loss to changes in epidermal development associated with the transition to aquatic life, we did not detect the same loss-of-function mutations. Instead, we identified extensive signatures of positive selection in mysticete and sirenian lineages, together with convergent evolution, indicating that TRPV3 has evolved under selective pressures in aquatic environments rather than being functionally lost. The use of genomes released after 2022 with higher levels of completeness may partly explain these differences (Table S1).

Overall, the evolution of TRP genes in aquatic mammals reflects both shared selective pressures associated with the transition to aquatic life and lineage-specific adaptations. While cetaceans and sirenians converged on similar sensory solutions for life fully underwater, each lineage also exhibits unique modifications, highlighting the combined influence of shared and independent adaptive trajectories.

### Lineage-Specific Gene Loss and Diversification of Sensory Genes Underpin Adaptation to Fully Aquatic Life and Distinct Diets and Environments

The higher accumulation of non-synonymous mutations in aquatic mammals may result from positive selection or a relaxation of purifying selection (Wertheim et al. 2015; Zhao et al. 2022), consistent with the rapid evolutionary dynamics of sensory receptors, which often lead to species-specific differences in their structural and functional properties (Cordero-Morales et al. 2012). The increase in ω values varies among aquatic lineages, with most genes exhibiting elevated values in Cetacea and particularly in Mysticeti. This pattern may reflect differences in life history traits among lineages.

#### Thermal and Chemical Adaptations in the Cetacea Clade

Cetaceans have a broad distribution: from freshwater to deep pelagic marine environments, across all oceans. Their heat-sensitive TRPs were mostly conserved and shaped by purifying selection. In contrast, we detected positive selection in cold receptor TRPM8, suggesting lineage-specific modifications associated with thermal adaptation (Saito and Saito 2025). This pattern differs from those observed in sirenians and may reflect the stronger selective pressures imposed by cold aquatic environments on cetaceans. Similar patterns of adaptive change have been reported in other cold-adapted mammals; for example, Lynch et al. (2015) identified amino acid substitutions in TRPM8 and TRPA1 of woolly mammoths that likely altered thermal sensitivity.

Pseudogenization events and gene loss can also be adaptive (Olson, 1999), especially in the context of environmental transition. In cetaceans, chemoreception is reduced (Kishida et al. 2015; Policarpo et al. 2024), and the possible inactivation of chemoreceptors we found further supports their diminished reliance on this sensory modality, consistent with the limited propagation of chemical signals in water (Kishida 2021), their reduced number of taste buds (Berta et al. 2007), and their carnivorous diet (Jiang et al. 2012; Policarpo et al. 2024).

We found that the sour taste receptors PKD1L3 and PKD2L1 are potentially pseudogenized in all cetaceans analyzed, with both genes carrying lineage-specific mutations in key functional domains. These parallel disruptions may have contributed to the reduction or loss of sour taste perception in cetaceans. In PKD2L1, mutations in the EF-hand domain, critical for calcium binding and receptor activation (Koulen et al. 2002), reinforce previous reports of gene inactivation (Zhu et al. 2014; Feng et al. 2014), and functional studies show that loss of PKD2L1 impairs sour taste signaling (Frings et al. 2010; Horio et al. 2011). Similarly, PKD1L3 shows substitutions in the Polycystin-1, Lipoxygenase, Alpha-toxin (PLAT); extracellular; and transmembrane domains that may disrupt channel assembly and membrane interactions, essential for receptor function (Li et al. 2003; Ishimaru et al. 2010). Another cetacean-specific change includes a switch from a conserved arginine to glycine at site 2225 within the ion-conducting pathway, which may further reduce ion permeability (Su et al. 2018). Finally, conserved synteny around both loci indicates that their inactivation most likely reflects local sequence decay rather than large-scale chromosomal rearrangements (Feng et al. 2014; Ghiurcuta and Moret, 2014; Zhu et al. 2014) (Fig. S43a). These findings may reflect a reduced ecological relevance of sour taste for cetaceans in fully aquatic environments, where dietary acidity and toxin exposure are low.

#### Chemo and Somatosensory Adaptations in Odontoceti

Toothed whales (odontocetes) are carnivorous predators that typically swallow large prey whole, reflecting a reduced functional role of chemoreception in foraging and feeding (Jiang et al. 2012; Kishida et al. 2015; Cabrera et al. 2021). Consistent with this pattern, we detected signs of inactivation not only in sour taste receptor genes, but also in the chemoreceptor TRPA1 and the taste receptor TRPM5, and an overall relaxation of chemoreceptors (TRPM4, TRPV1, and TRPV3). These findings align with broader patterns of relaxed selection in chemosensory genes of aquatic mammals, often associated with pseudogenization or neutral evolution (Chikina et al. 2016).

Although the thermo and chemoreceptor TRPA1 is highly conserved across metazoans, particularly in mammals (Hoffstaetter et al. 2018; Talavera et al. 2020; Himmel and Cox 2020), we found that all analyzed odontocetes have lost about 70% of their coding sequence, likely after diverging from mysticetes, similarly to findings of Wu et al. (2026). The missing region encompasses key domains and functional sites of the protein, possibly indicating the loss of core receptor functions related to channel gating (Cordero-Morales et al. 2012; Laursen et al. 2015; Moparthi et al. 2022) (Fig. 2b). Additionally, it was under relaxed purifying selection in toothed whales, probably associated with the pseudogenization process (Wertheim et al. 2015; Cole et al. 2022; Zhao et al. 2022; Wu et al. 2026). Moreover, the syntenic context revealed that the loss of the upstream genes, combined with the pseudogenization of adjacent loci, suggests a broader degradation of the genomic neighborhood surrounding this locus (Fig. S43c).

The TRPM5 channel is expressed in taste receptor cells, where, together with TRPM4, it mediates sweet, bitter, and umami signal transduction and contributes to thermosensation (Pérez et al. 2002; Hofmann et al. 2003; Damak et al. 2006). We identified inactivating mutations in key domains of TRPM5 across all odontocetes (Fig. 2c), along with evidence of relaxed selective constraints on TRPM4. Functional studies in TRPM5 knockout mice revealed loss of TRPM5 expression and reduced taste perception (Zhang et al. 2003; Damak et al. 2006), supporting that disruptions in critical regions of this gene contribute to taste loss. Despite this inactivation, overall synteny was maintained, indicating that TRPM5 loss likely represents a lineage-specific pseudogenization event (Fig. S43d). Together, these findings reinforce the loss of sweet, bitter, and umami taste perception in odontocetes, previously attributed to the pseudogenization of their upstream taste receptors T1R and T2R (Jiang et al. 2012; Policarpo et al. 2024), as both TRPM4 and TRPM5 are essential for the transduction of these taste modalities (Dutta Banik et al. 2018).

Odontocetes inhabit a wide range of environments, from tropical coastal zones to polar seas and even freshwater systems, exhibiting ecological adaptations to diverse thermal and environmental conditions (Berta et al. 2007). In this context, mechano- and thermoreceptors might play an important role in survival. The positive selection in genes that are exclusively somatoreceptors (TRPM2, TRPM8, and TRPV2) and do not have a role in chemoreception may be associated with adaptations in navigation and survival in different thermal environments.

We suggest that signatures of positive selection in odontocete TRPM8 reflect adaptive modifications to the aquatic thermal environment, possibly compensating for the loss of TRPA1. Experimental evidence supports this compensatory role: in TRPA1-knockout mice, TRPM8 can be activated through alternative transduction pathways, indicating that cold perception is maintained even in the absence of TRPA1 (Jordt et al. 2004; Bautista et al. 2007; Knowlton et al. 2010; Zhang et al. 2022). Moreover, in the noxious heat and mechanoreceptor TRPV2, a non-conservative substitution at site 305 (Fig. 6) introduces a negative charge into a domain involved in protein–protein interactions and channel regulation. This alteration likely affects conformational dynamics and structural integrity, potentially altering channel function (Cordero-Morales et al. 2012; Zhang et al. 2023), as reflected by the increased protein instability observed in odontocete TRPV2 sequences. This suggests structural divergence in odontocete TRPV2 relative to terrestrial counterparts, which could implicate adaptive modification of receptor function in these species.

#### Chemo and Somatosensory Adaptations in Mysticeti

Most baleen whales exhibit pronounced seasonal migrations driven largely by ocean temperature gradients and prey availability. During summer, they inhabit cold, food-rich high-latitude waters to feed, whereas in winter they migrate to warmer tropical or subtropical regions to reproduce (Berta et al. 2007). Possibly reflecting adaptations to these diverse thermal environments, we found positive selection in cold receptor TRPM8 in mysticetes.

**Fig. 7:**
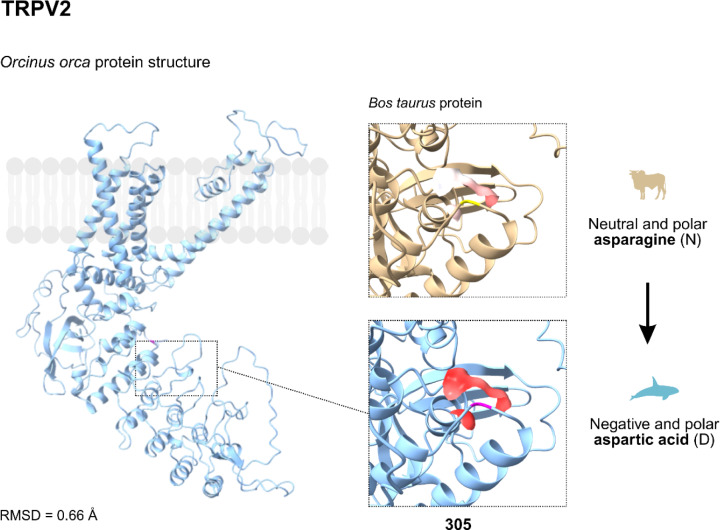
Ribbon diagrams of a single subunit of the *B. musculus* TRPM8 protein structures predicted by AlphaFold3. Domains, binding sites, and PSS are color-coded: light blue, cytoplasmic domains (N- and C-termini); dark blue, transmembrane domains (S1–S6); gray, extracellular loops; dark green, pore helix; orange, coiled-coil domain; red, active binding sites; purple, PSS in Cetacea. Numbers in bold correspond to PSSs. Sites are highlighted and shown in close-up views for improved visualization and comparison. Annotations were retrieved from Uniprot and based on Zhao et al. (2022)

Most positively selected sites were located within the N-terminal melastatin homology regions (MHR)1–2 (Fig. 7), which are essential for temperature sensitivity (Laursen et al. 2015, 2016; Lu et al. 2022). Additionally, a conservative isoleucine-to-valine substitution at site 1138 (Fig. 7) occurs within the pore region, where residue hydrophobicity influences cold activation efficiency (Yang et al. 2020; Lu et al. 2023). Because hydrophobicity is conserved, thermosensitivity is likely maintained. Moreover, no mutations were detected at site 928, whose alteration abolishes cold-evoked channel activity (Palchevskyi et al. 2023), further suggesting preserved TRPM8 thermosensitivity in these species. Mutations in the MHR1-3 and pore region may enhance TRPM8 cold activation and contribute to species-specific cold sensitivity of animals, thereby supporting adaptive responses to thermal fluctuations (Lynch et al. 2015; Lu et al. 2022, 2023).

Positive selection in TRPM8 has been linked to temperature adaptation in wild vertebrates, as its cold activation tuning influences both thermal tolerance and habitat selection (Yang et al. 2020; Lu et al. 2023). For example, penguins and squirrels have adapted to extreme cold by reducing TRPM8 cold sensitivity (Matos-Cruz et al. 2017; Yang et al. 2020). Similarly, our findings suggest that positive selection may have contributed to the thermal adaptations of toothed and baleen whales to their aquatic environments (Lu et al. 2023).

Heat receptors and mechanoreceptors were overall under purifying selection in mysticetes, probably related to the maintenance of stimuli perception and survival. However, the heat-sensitive chemoreceptors TRPM4 and TRPV3, as well as the chemoreceptor TRPM5, showed evidence of positive selection in mysticetes. Selection analysis indicated adaptive changes in TRPV3, suggesting an evolutionary response to cold environments (Lynch et al. 2015). Although most chemosensory genes evolve under relaxed selective constraints in aquatic mammals (Chikina et al. 2016), the selective pressures acting on these genes in mysticetes may represent adaptive adjustments to the ecological and feeding demands associated with filter feeding.

At site 1004 of TRPM4, near the pore helix, mysticetes exhibit multiple derived and non-conservative substitutions. These changes, involving both transitions and transversions, introduce residues with distinct side-chain properties, suggesting independent mutation events. Because the pore region is highly conserved and essential for ion selectivity, even small alterations at this position could meaningfully affect channel permeability (Nilius et al. 2005). Another substitution at site 1204, within the conserved coiled-coil domain (Tsuruda et al. 2006), replaces a lysine, forming three hydrogen bonds with neighboring residues in terrestrial mammals, with glutamic acid in mysticetes, disrupting one of these interactions (Fig. 5). Together, these modifications may underlie the increased instability of mysticete TRPM4 protein relative to terrestrial artiodactyls, reflecting lineage-specific structural and possible functional adaptations.

#### Chemo and Somatosensory Adaptations in Sirenia

Unlike cetaceans, sirenians occupy restricted, shallow coastal, riverine, and estuarine habitats (Berta et al. 2007) and rely heavily on touch and taste for foraging and survival (Bauer and Reep 2018; Moore et al. 2022). Consistent with this sensory reliance, most TRP chemoreceptors in Sirenia are under purifying selection, with only three showing evidence of positive selection: TRPM4, TRPV2 (also heat and mechanoreceptors), and TRPV3 (also a heat receptor). These genes also exhibit increased protein instability, suggesting structural shifts. Such changes may reflect adaptation to the warm tropical and equatorial waters inhabited by sirenians, where higher water temperatures impose distinct thermal challenges. Indeed, manatees are sensitive to heat stress and often migrate to cooler waters to avoid overheating (Davis 2019; Hardy et al. 2019). Additionally, mechanoreception is crucial for sirenians inhabiting shallow and riverine environments, where dense vegetation and frequent physical obstacles demand precise tactile navigation (Moore et al. 2022), a reliance that may have contributed to the evolutionary pressures acting on mechanosensitive TRP receptors.

In sirenians, fully aquatic herbivores that feed primarily on seagrasses and algae (Berta et al. 2007), taste receptors likely play a key role in dietary selection and toxin avoidance, reflecting long-term ecological pressures on their sensory systems (Moore et al. 2022; Policarpo et al. 2024). Comparative studies show that taste receptor repertoires evolve in close association with diet; herbivorous species often retain or diversify bitter and related receptors to cope with plant-derived toxins (Li and Zhang 2014; Itoigawa et al. 2024).

While TRPM5 appears to be lost in odontocetes, sirenians retain an intact copy. The preservation of this gene, together with signatures of positive selection in TRPM4, may reflect adaptation to an herbivorous diet, consistent with the maintenance of upstream T1R receptors in this lineage (Moore et al. 2022; Policarpo et al. 2024). Likewise, although PKD1L3 and PKD2L1 are potentially lost in cetaceans, both genes remain intact in sirenians.

Notably, site 756 of PKD1L3 in trichechids lies within the highly variable N-terminal extracellular domain, which is critical for ligand binding; substitutions in this region may influence receptor–ligand interactions (Chen et al. 2011) and are consistent with potential shifts in sour taste sensitivity associated with the pH variability and acidic metabolites of seagrasses and freshwater plants. Taken together, these patterns indicate that dietary pressures, particularly the shift to herbivory, rather than aquatic life alone, may have shaped the retention and diversification of specific taste-related genes in sirenians.

Future genomic analyses of non-coding regions and functional studies are needed to experimentally assess the impact of positively selected and inactivating mutations on protein function, sensory physiology, and ecological adaptation of aquatic mammals, offering broader insights into vertebrate sensory evolution.

## Conclusion

In conclusion, our results indicate that the transition from terrestrial to fully aquatic life may have shaped the evolution of TRP genes in both Cetacea and Sirenia. However, because these lineages underwent this transition independently, their evolutionary trajectories diverged, as also observed in other sensory systems such as vision and hearing, where distinct adaptations have arisen. Still, in line with broader patterns of convergent evolution among fully aquatic vertebrates, we detected cases of convergence between the two groups. Environmental transition alone, however, was likely not the only driver; differences in diet and habitat also contributed to further divergence. Specifically, the evolution of taste, thermo-, and mechanoreceptors may reflect the interplay between dietary specialization and ecological demands: taste receptor loss is associated with carnivory in cetaceans, receptor maintenance with herbivory in sirenians, and thermo- and mechanoreceptor adaptations with thermoregulation and survival in distinct habitats. Convergently, both lineages show signs of adaptation on mechano- and thermoreceptors as key components for survival in aquatic environments.

## Supplementary Information

Below is the link to the electronic supplementary material.

Supplementary file1 (PDF 1121 KB)

Supplementary file2 (DOCX 17021 KB)

Supplementary file3 (DOCX 61 KB)
