# Supplementary material for: Sensing Underwater: Diversifying Selection, Convergent Evolution and Inactivation in Sensory Receptors’ Genes of Aquatic Mammals
Source: J Mol Evol. 2026 Jul 1;94(4):629–54. doi: 10.1007/s00239-026-10327-2 (PMC13433407; doi:10.1007/s00239-026-10327-2)
Supplement: Supplementary file 1 [file 239_2026_10327_MOESM1_ESM.pdf]

Sensing underwater: diversifying selection, convergent evolution and inactivation in sensory receptors' genes of aquatic mammals for life underwater

Journal of Molecular Evolution

Ana Luiza Lein-Borba[1*], Giovanna Selleghin-Veiga[1], Beatriz Daros[1], Letícia Magpali[2], Mariana F. Nery[1*]

[1]University of Campinas (Department of Genetics, Evolution, Microbiology and Immunology), Campinas, SP, Brazil

[2]Dalhousie University (Department of Biology), Halifax, Nova Scotia, Canada

[*]Corresponding author

E-mail addresses: analuizaclein@gmail.com (ALLB), mariananery@gmail.com (MFN)

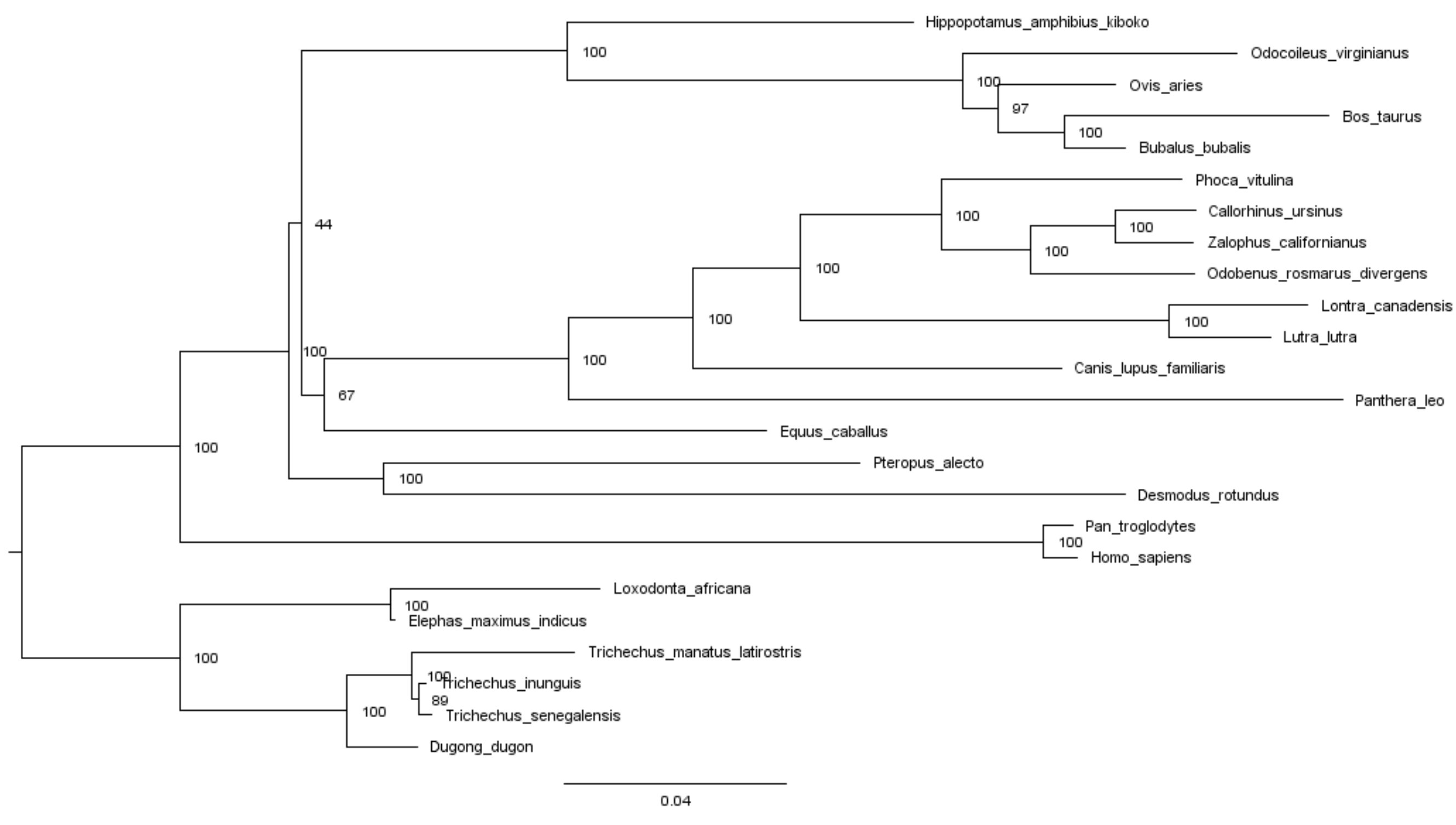

Figure S1. Maximum likelihood phylogenetic tree reconstructed with IQ-TREE 2 using the nucleotide alignment of PKD1L3 gene without sequences with evidence of inactivation. Branch confidence is determined by bootstrap values in the nodes and branch lengths are in scale.

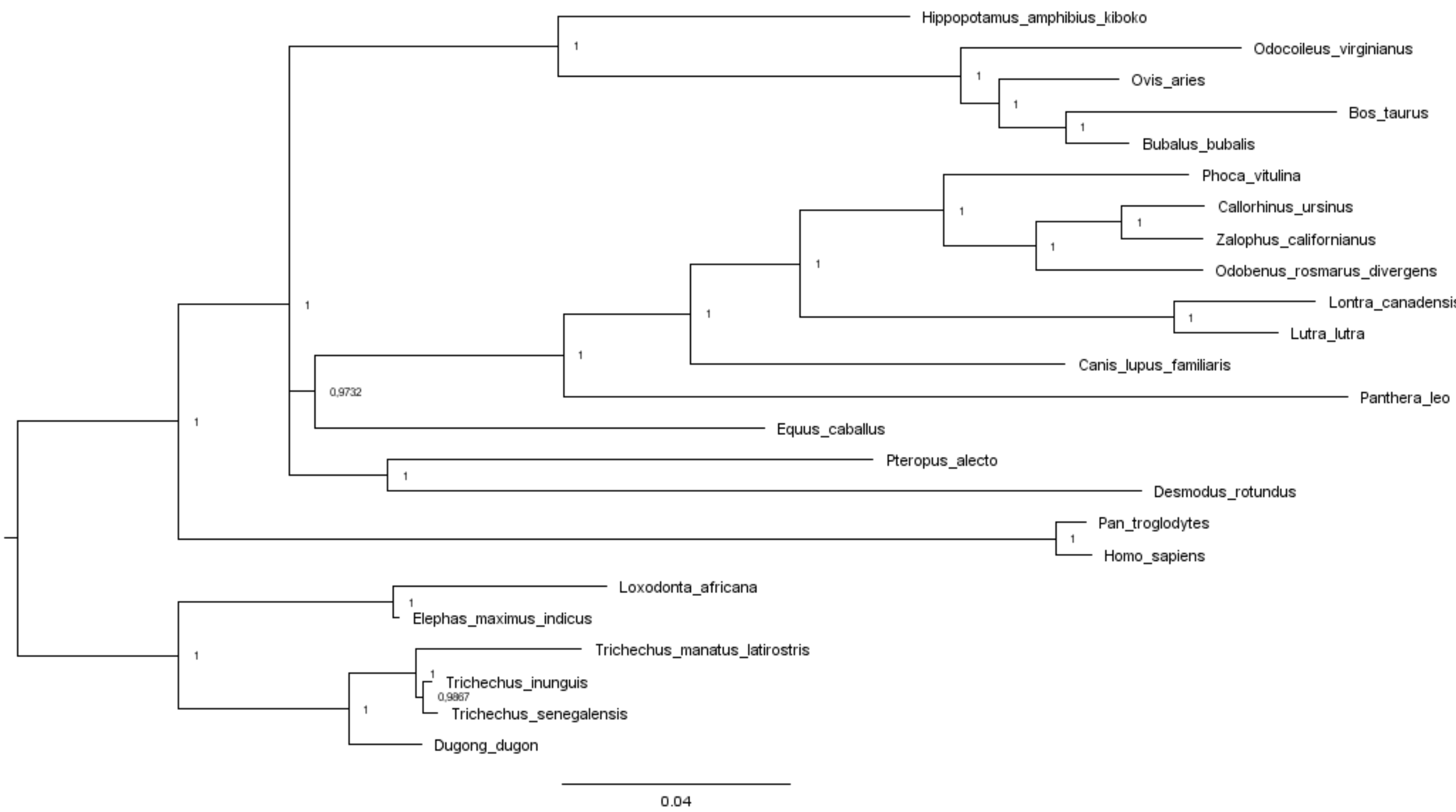

Figure S2. Bayesian phylogenetic tree reconstructed with MrBayes v. 3.2.7a using PartitionFinder v. 2.1.1 evolutionary models of nucleotide alignment of PKD1L3 gene without sequences with evidence of inactivation. Support values for the branches are given by posterior probabilities in the nodes. Branch lengths are in scale.

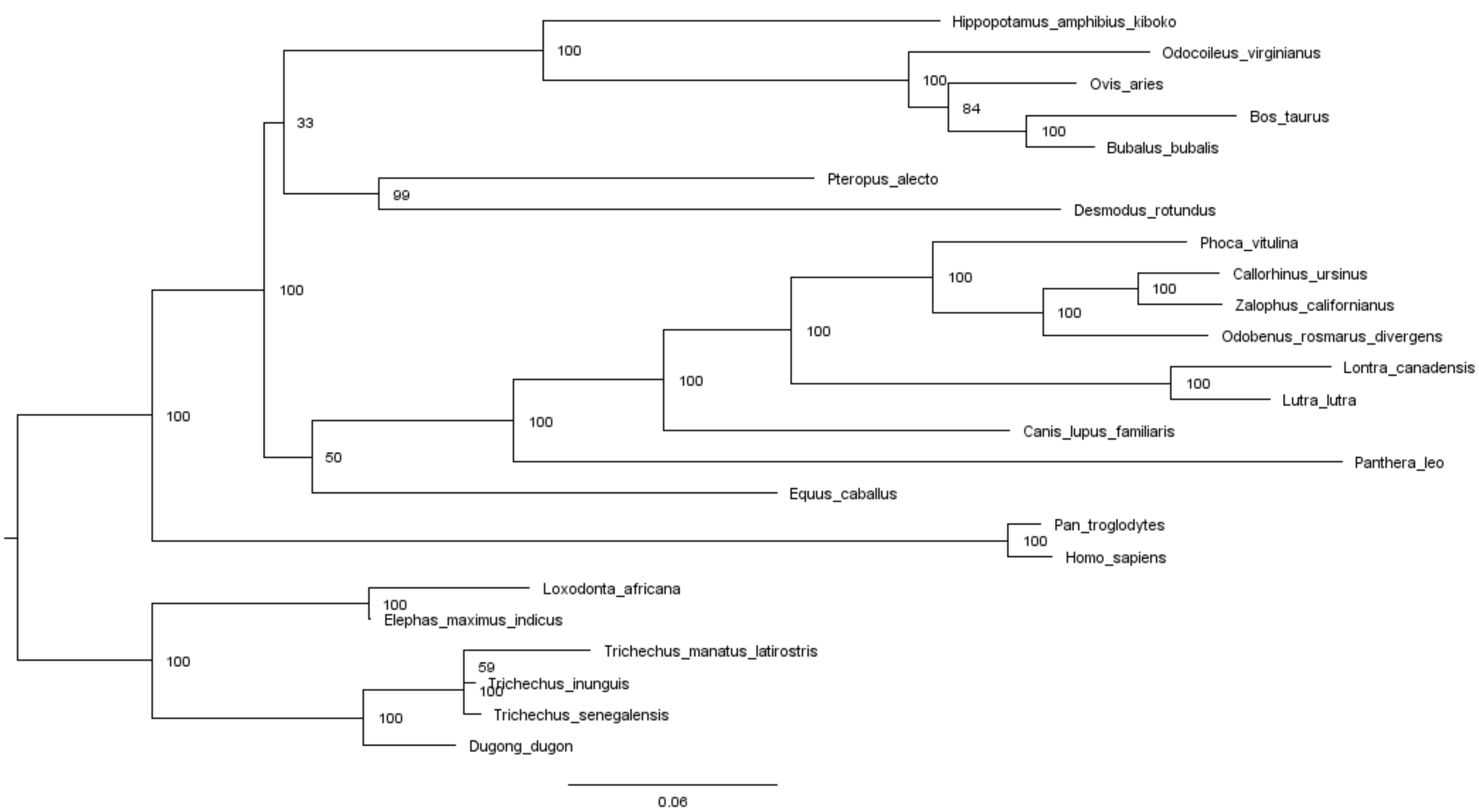

Figure S3. Maximum likelihood phylogenetic tree reconstructed with IQ-TREE 2 using the amino acid alignment of PKD1L3 gene without sequences with evidence of inactivation. Branch confidence is determined by bootstrap values in the nodes and branch lengths are in scale.

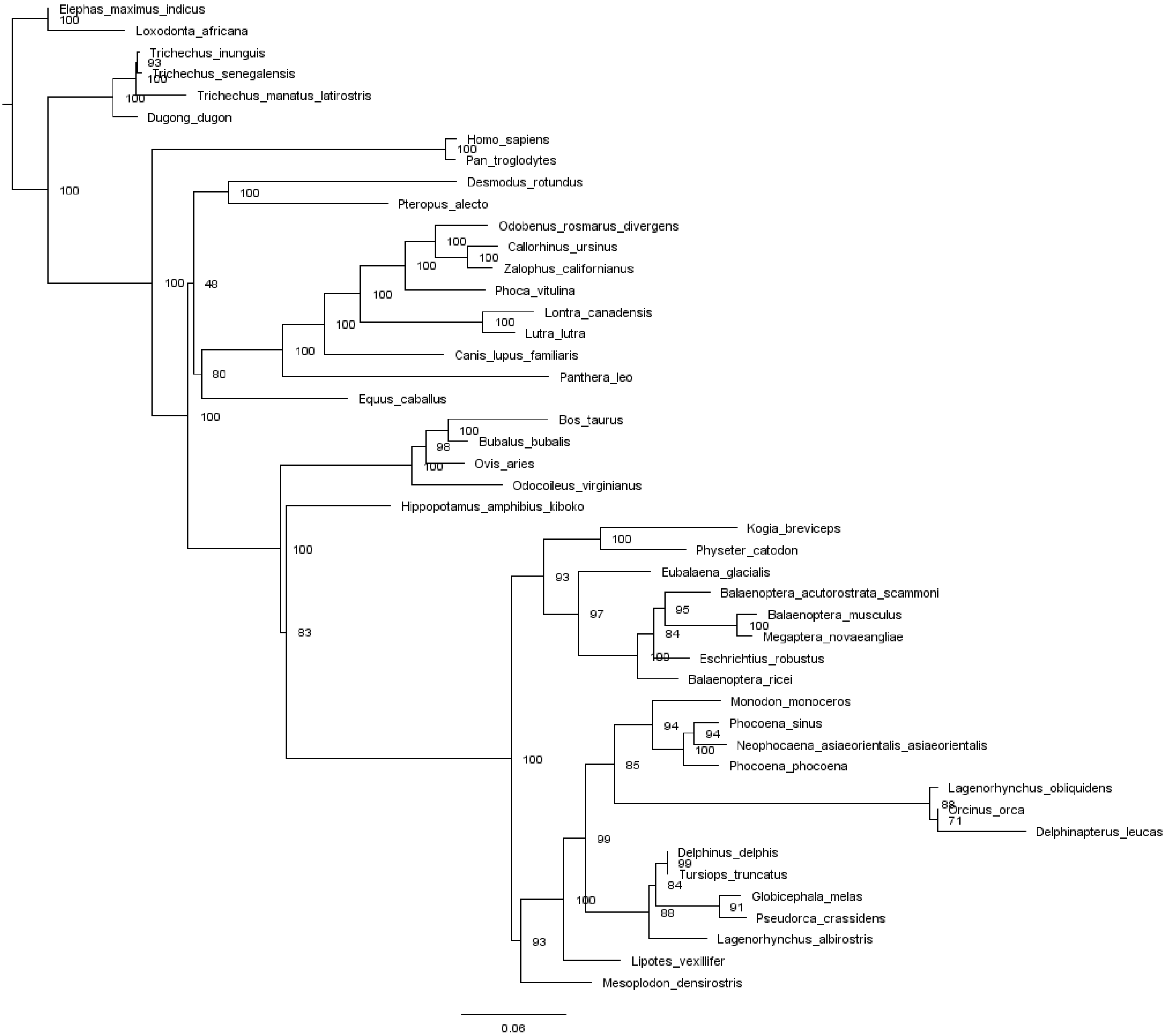

Figure S4. Maximum likelihood phylogenetic tree reconstructed with IQ-TREE 2 using the nucleotide alignment of PKD1L3 gene with the full dataset. Branch confidence is determined by bootstrap values in the nodes and branch lengths are in scale.

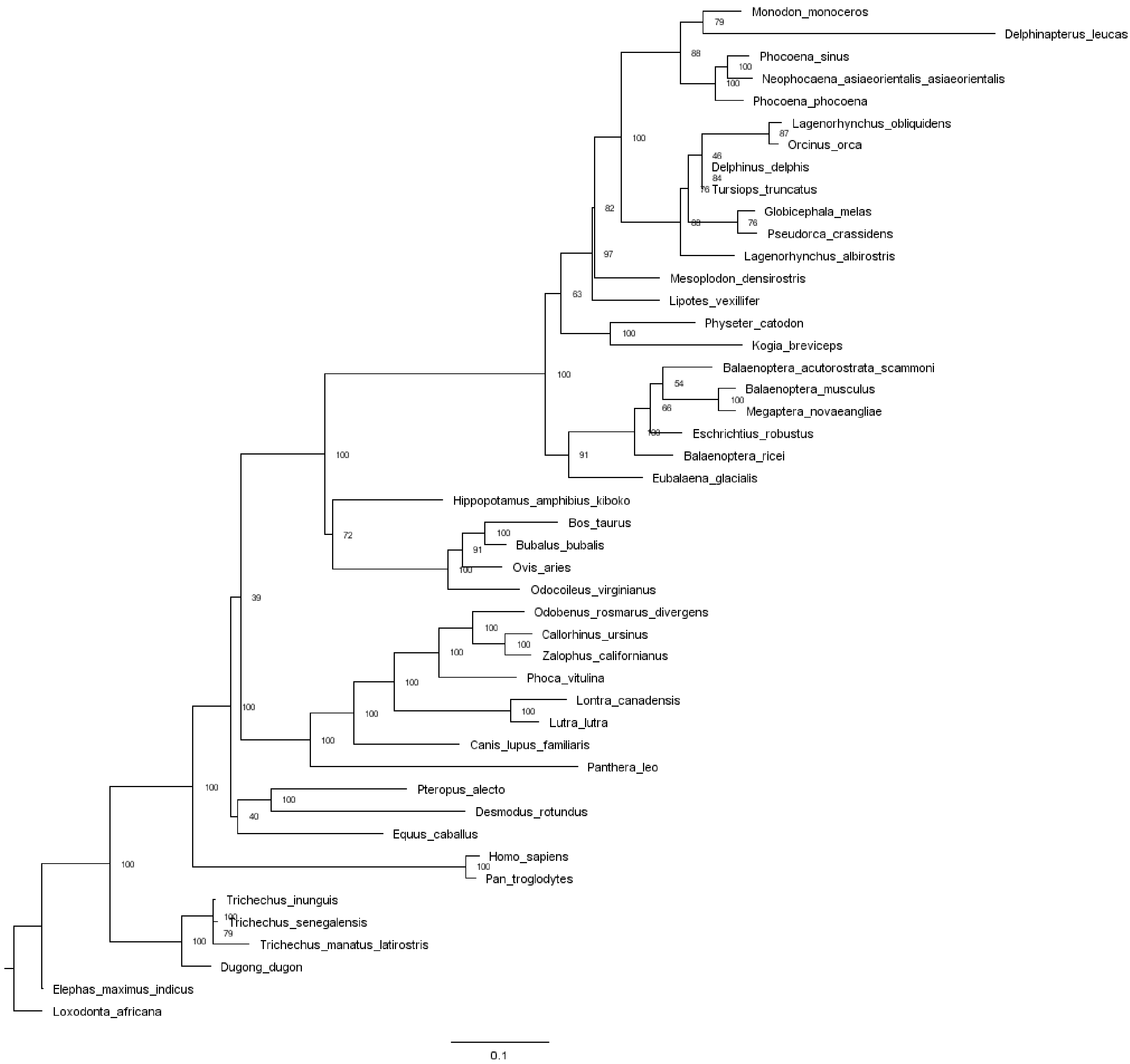

Figure S5. Maximum likelihood phylogenetic tree reconstructed with IQ-TREE 2 using the amino acid alignment of PKD1L3 gene with the full dataset. Branch confidence is determined by bootstrap values in the nodes and branch lengths are in scale.

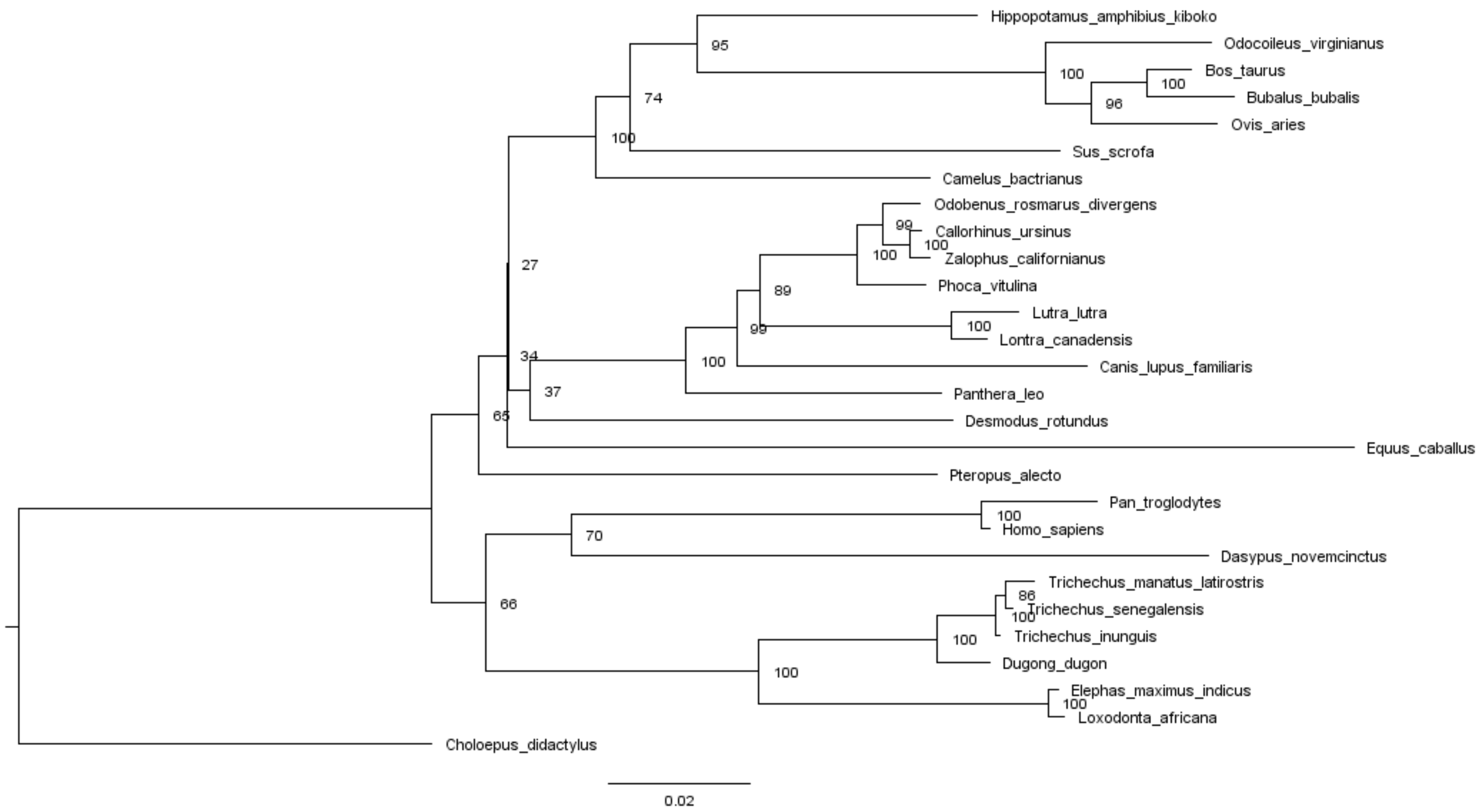

Figure S6. Maximum likelihood phylogenetic tree reconstructed with IQ-TREE 2 using the nucleotide alignment of PKD2L1 gene without sequences with evidence of inactivation. Branch confidence is determined by bootstrap values in the nodes and branch lengths are in scale.

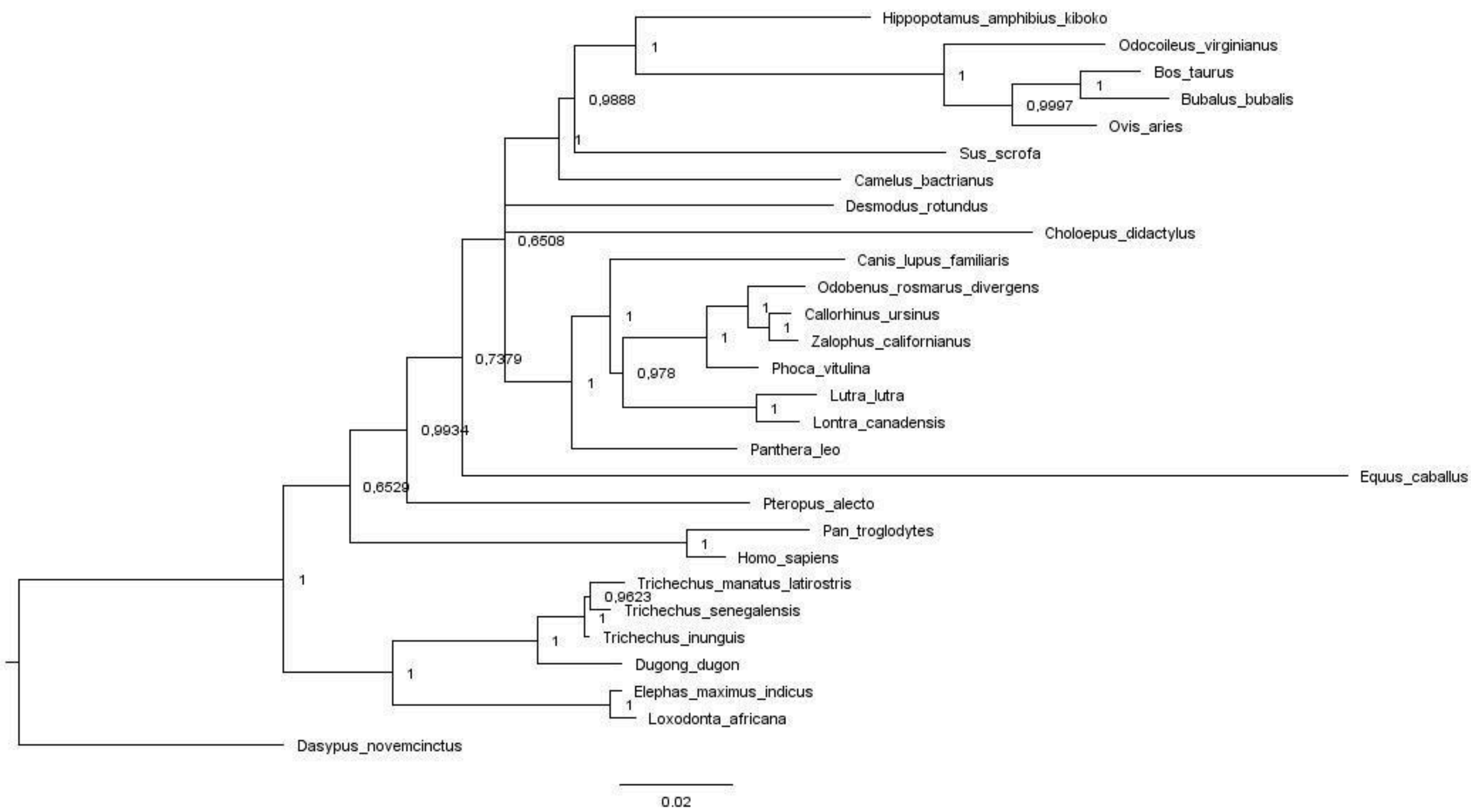

Figure S7. Bayesian phylogenetic tree reconstructed with MrBayes v. 3.2.7a using PartitionFinder v. 2.1.1 evolutionary models of nucleotide alignment of PKD2L1 gene without sequences with evidence of inactivation. Support values for the branches are given by posterior probabilities in the nodes. Branch lengths are in scale.

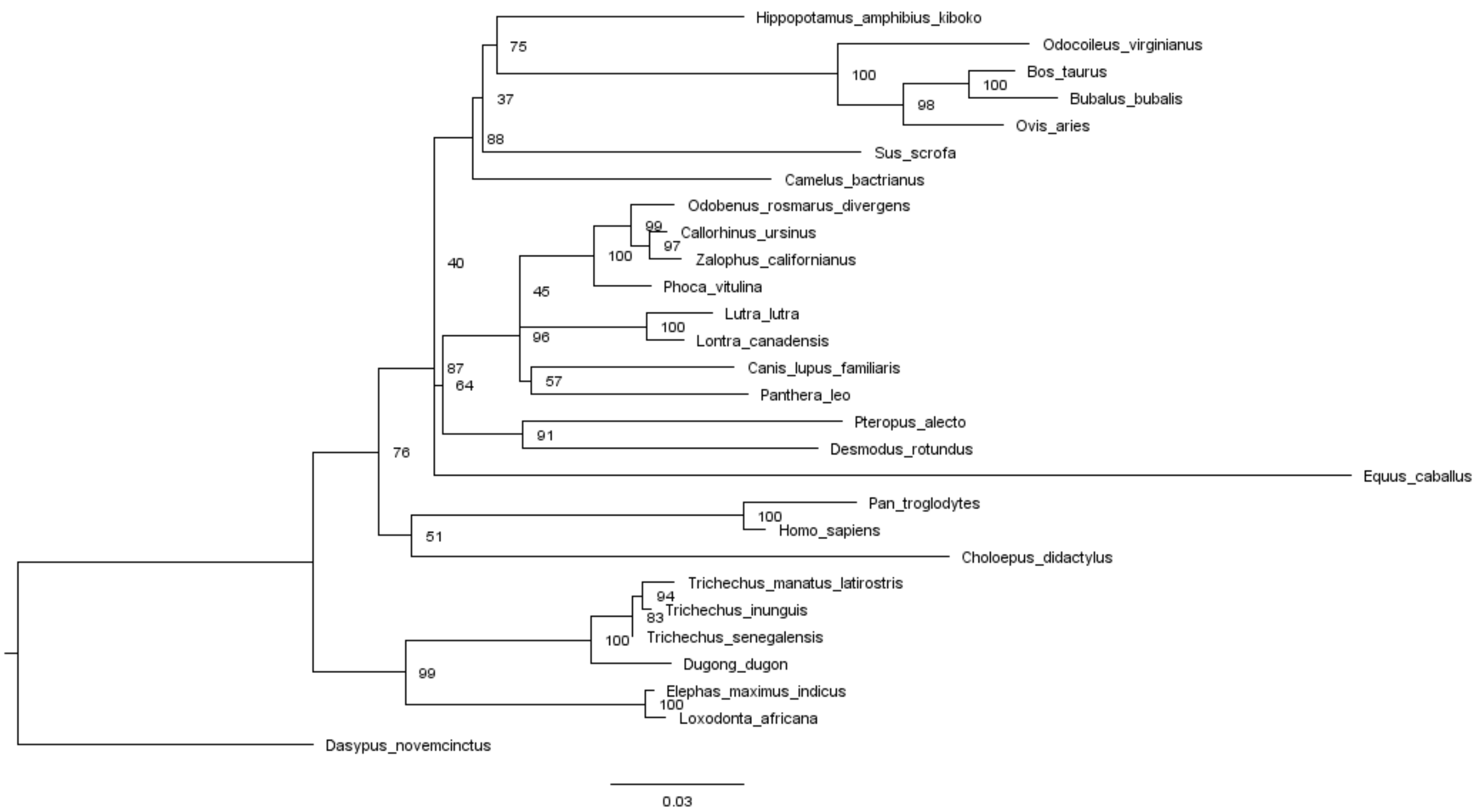

Figure S8. Maximum likelihood phylogenetic tree reconstructed with IQ-TREE 2 using the amino acid alignment of PKD2L1 gene without sequences with evidence of inactivation. Branch confidence is determined by bootstrap values in the nodes and branch lengths are in scale.

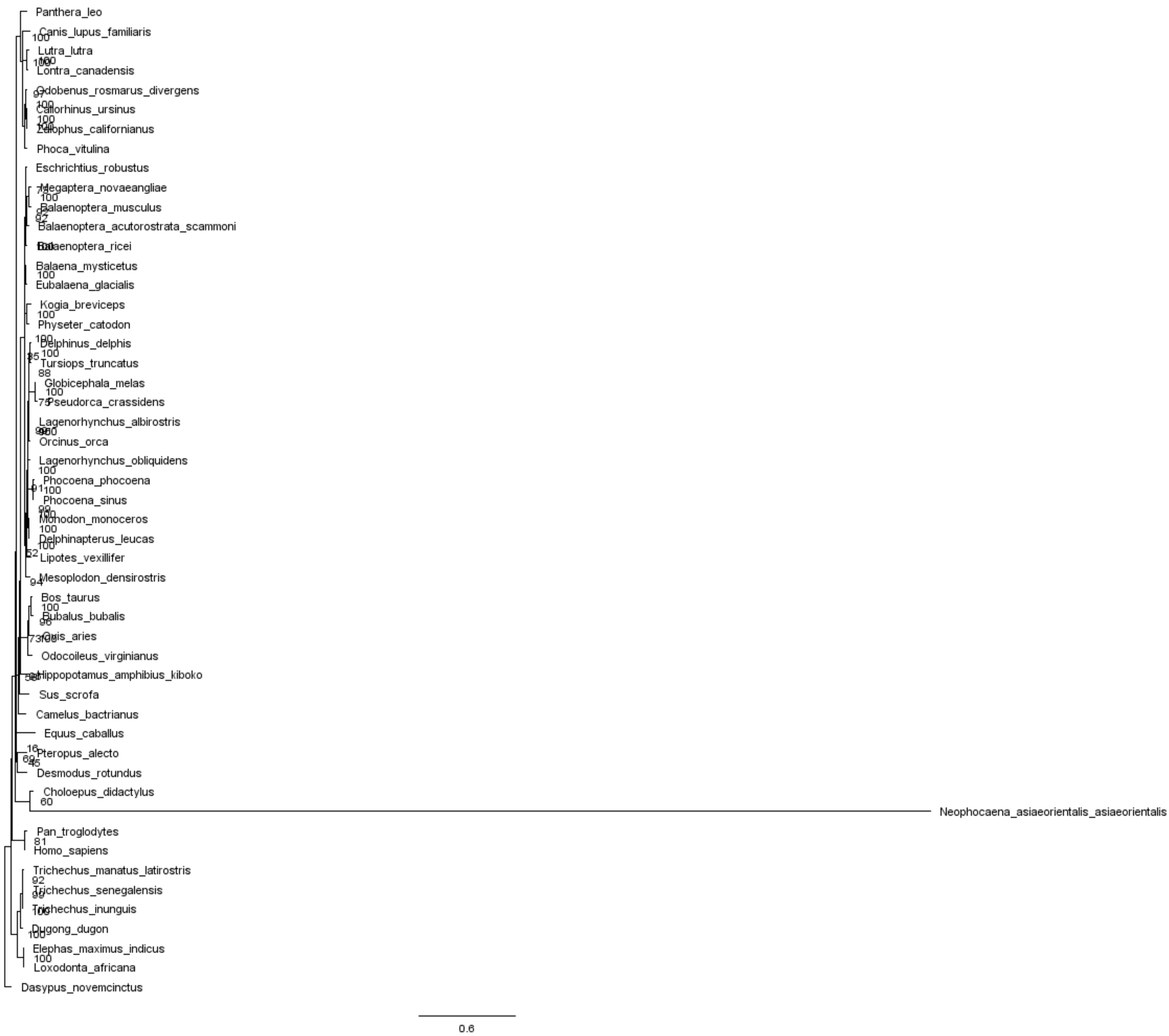

Figure S9. Maximum likelihood phylogenetic tree reconstructed with IQ-TREE 2 using the nucleotide alignment of PKD2L1 gene with the full dataset. Branch confidence is determined by bootstrap values in the nodes and branch lengths are in scale.

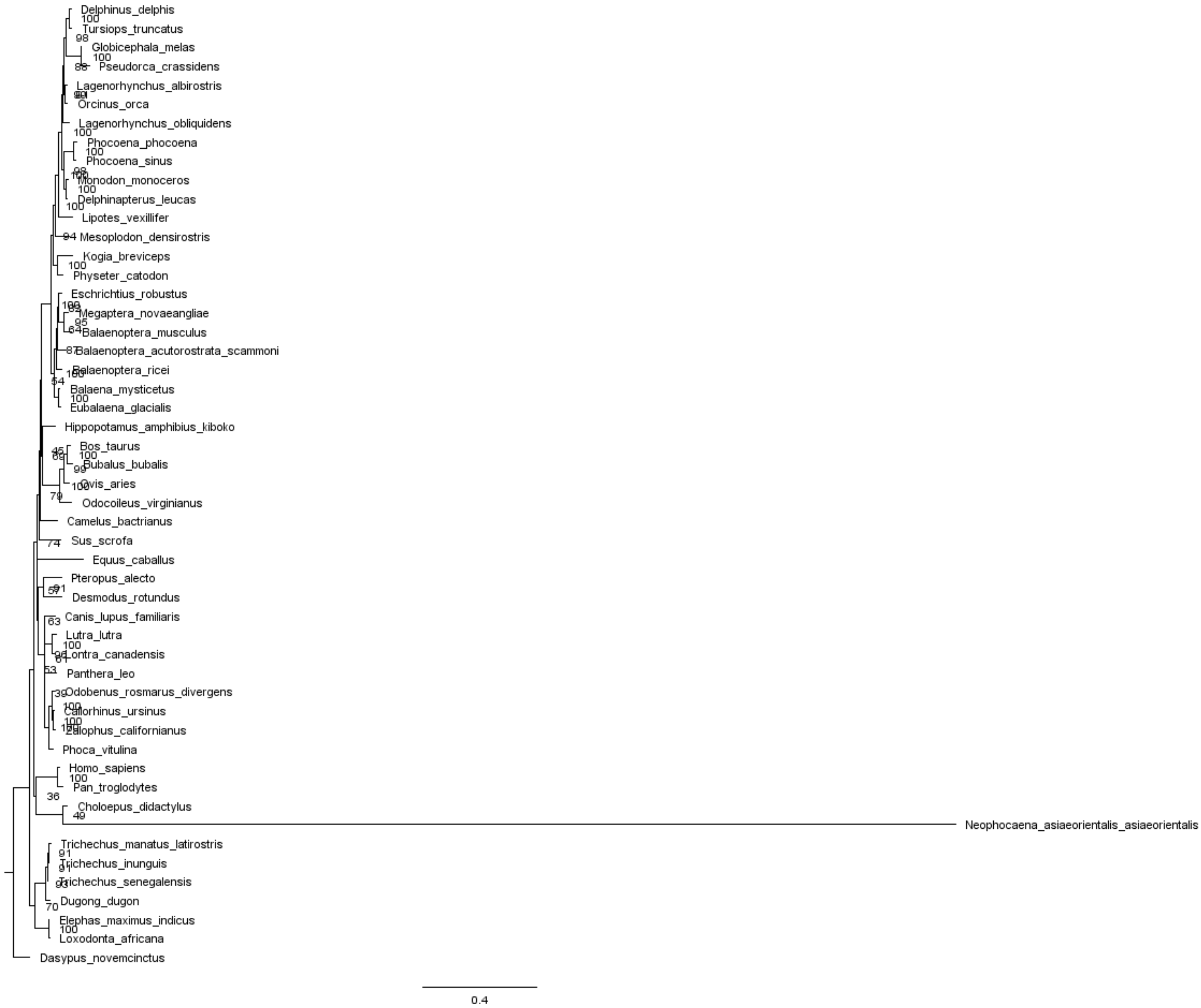

Figure S10. Maximum likelihood phylogenetic tree reconstructed with IQ-TREE 2 using the amino acid alignment of PKD2L1 gene with the full dataset. Branch confidence is determined by bootstrap values in the nodes and branch lengths are in scale.

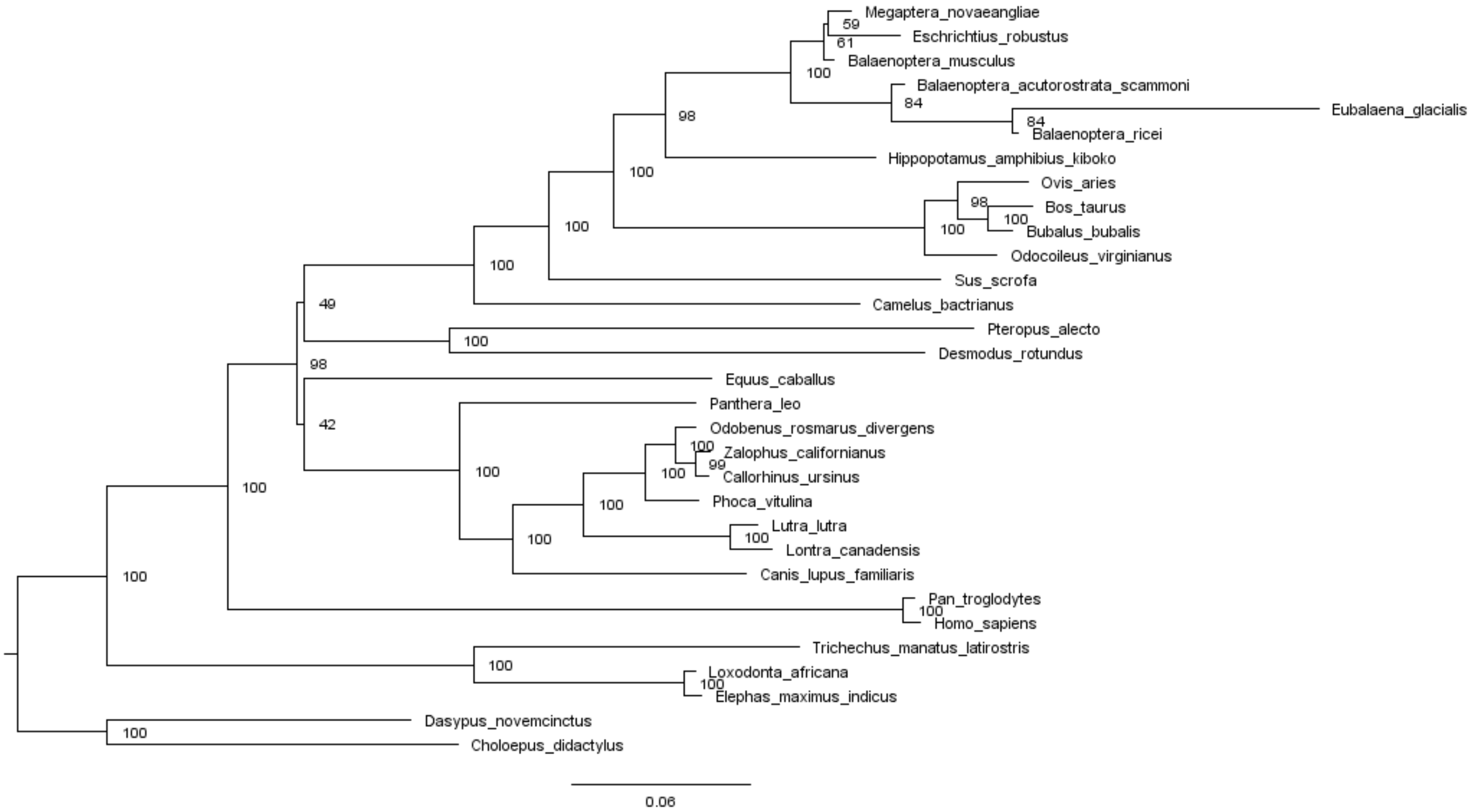

Figure S11. Maximum likelihood phylogenetic tree reconstructed with IQ-TREE 2 using the nucleotide alignment of TRPA1 gene without sequences with evidence of inactivation. Branch confidence is determined by bootstrap values in the nodes and branch lengths are in scale.

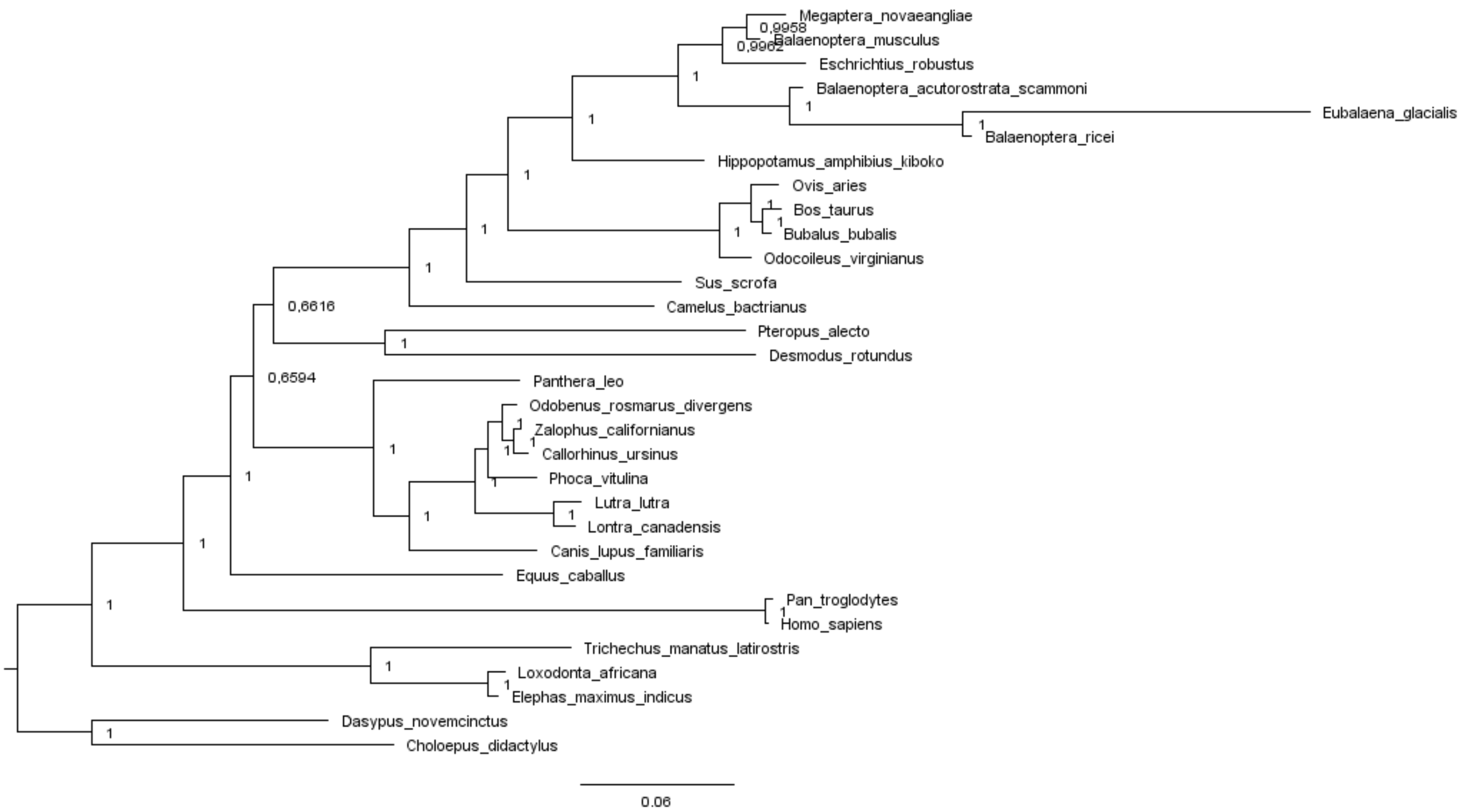

Figure S12. Bayesian phylogenetic tree reconstructed with MrBayes v. 3.2.7a using PartitionFinder v. 2.1.1 evolutionary models of nucleotide alignment of TRPA1 gene without sequences with evidence of inactivation. Support values for the branches are given by posterior probabilities in the nodes. Branch lengths are in scale.

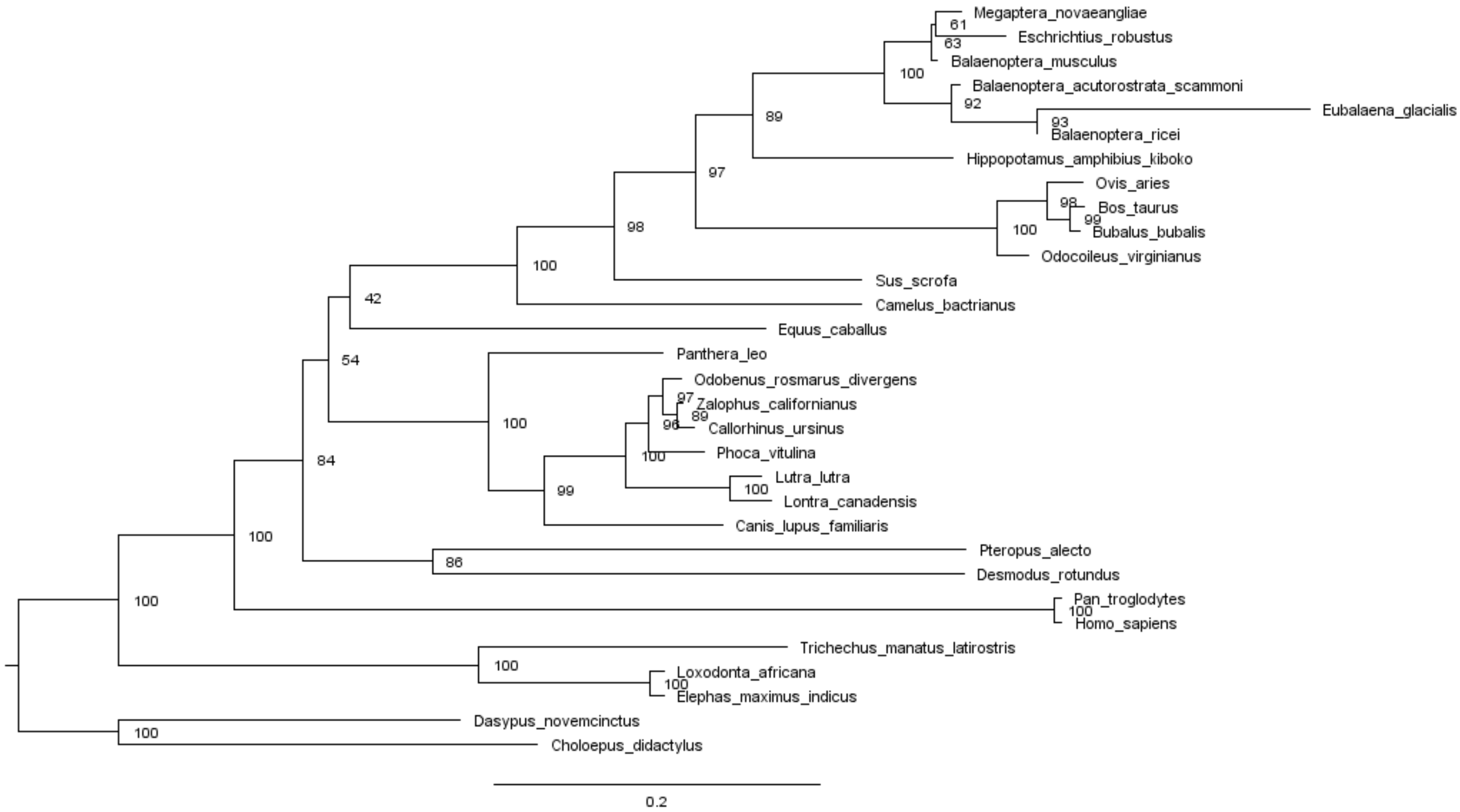

Figure S13. Maximum likelihood phylogenetic tree reconstructed with IQ-TREE 2 using the amino acid alignment of TRPA1 gene without sequences with evidence of inactivation. Branch confidence is determined by bootstrap values in the nodes and branch lengths are in scale.

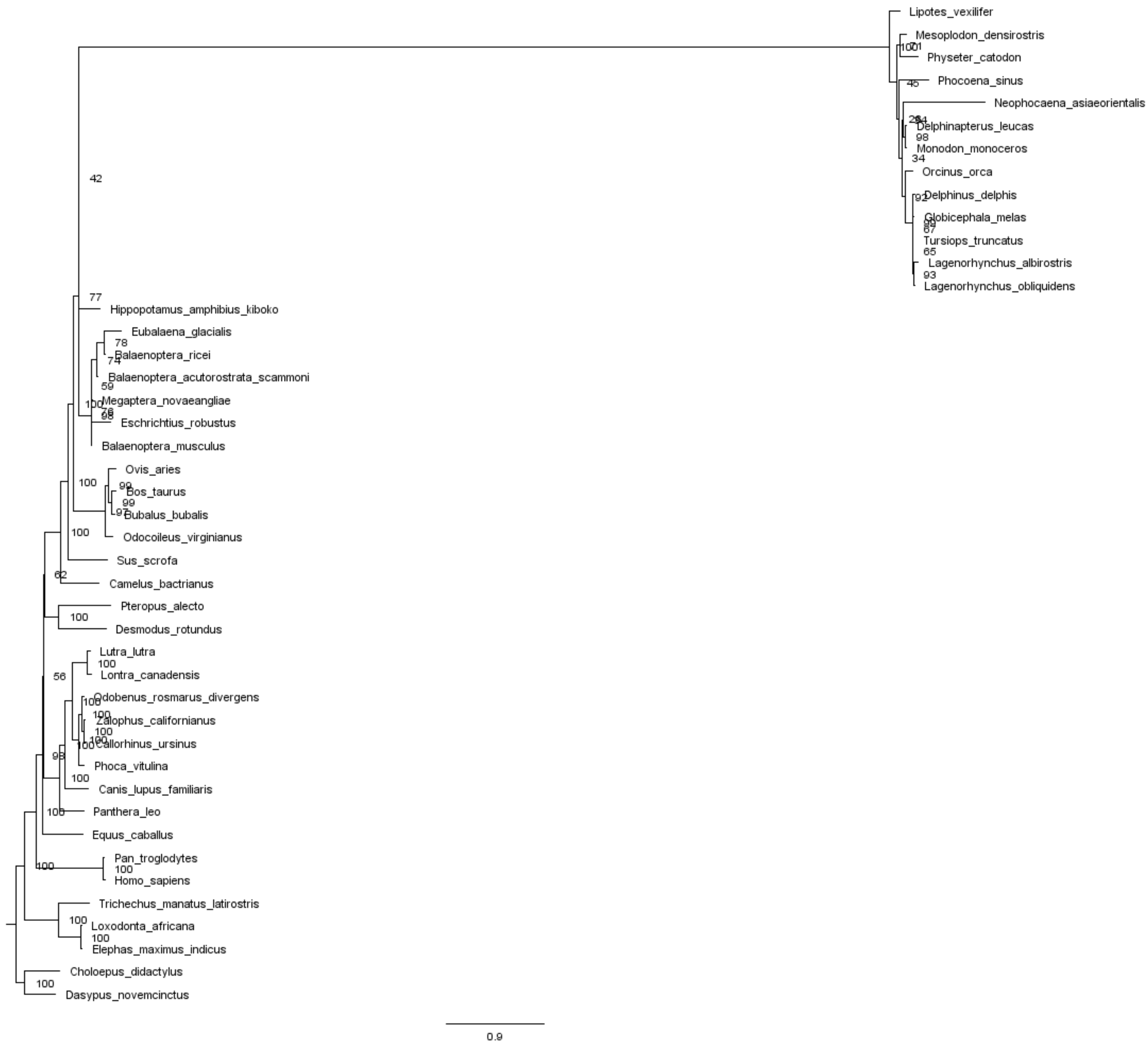

Figure S14. Maximum likelihood phylogenetic tree reconstructed with IQ-TREE 2 using the nucleotide alignment of TRPA1 gene with the full dataset. Branch confidence is determined by bootstrap values in the nodes and branch lengths are in scale.

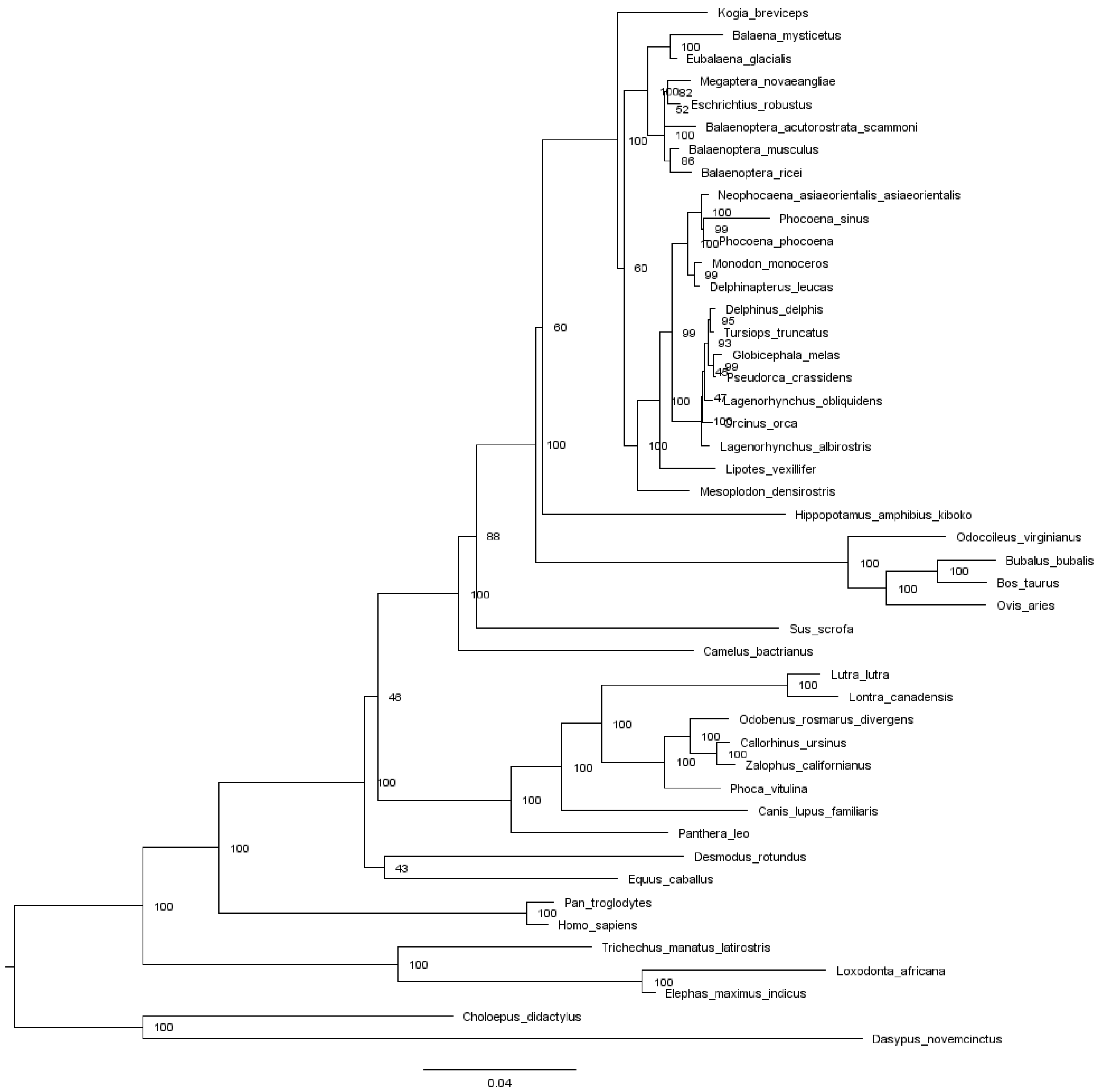

Figure S15. Maximum likelihood phylogenetic tree reconstructed with IQ-TREE 2 using the nucleotide alignment of TRPM2 gene. Branch confidence is determined by bootstrap values in the nodes and branch lengths are in scale.

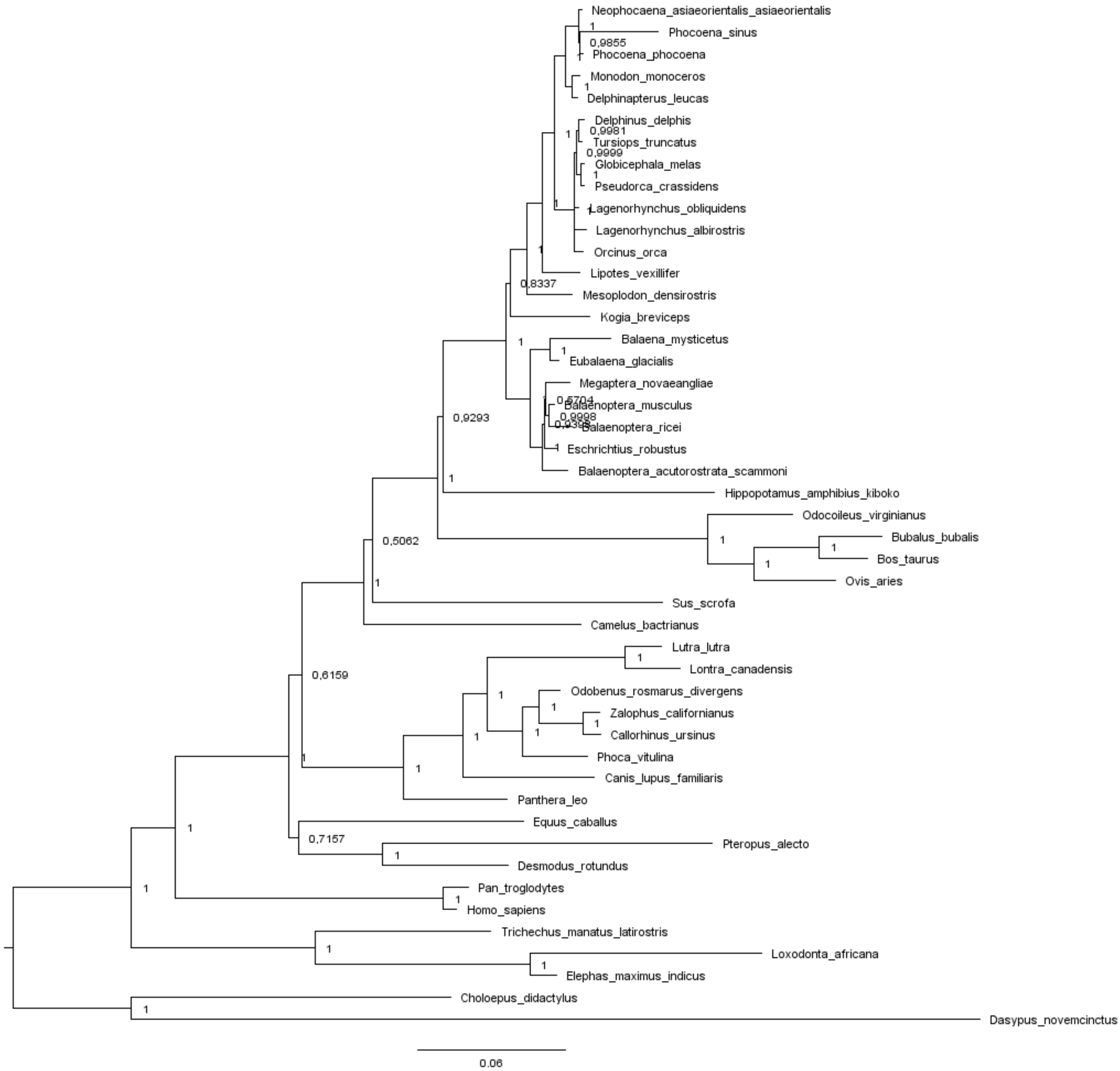

Figure S16. Bayesian phylogenetic tree reconstructed with MrBayes v. 3.2.7a using PartitionFinder v. 2.1.1 evolutionary models of nucleotide alignment of TRPM2 gene. Support values for the branches are given by posterior probabilities in the nodes. Branch lengths are in scale.

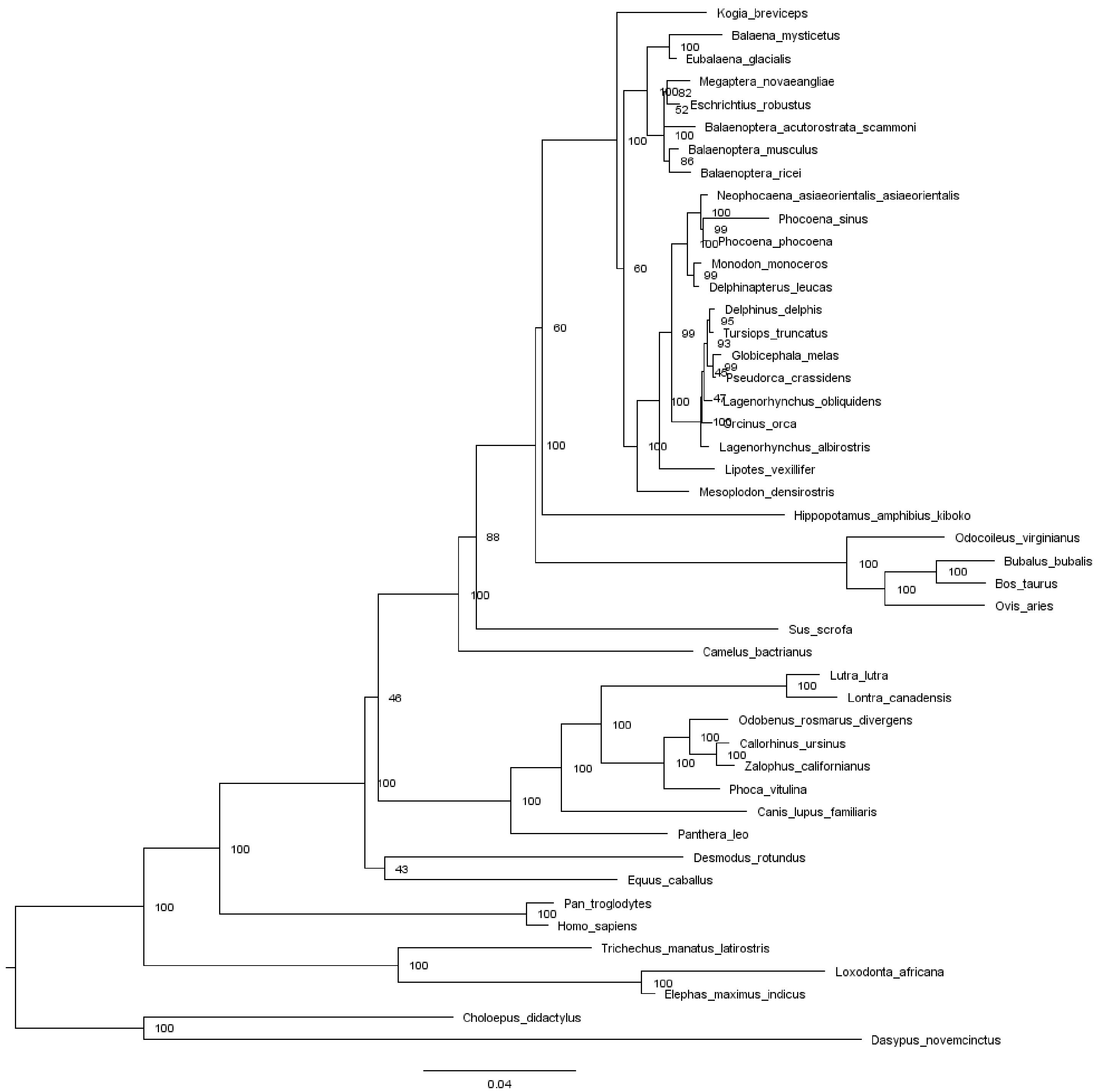

Figure S17. Maximum likelihood phylogenetic tree reconstructed with IQ-TREE 2 using the amino acid alignment of TRPM2 gene. Branch confidence is determined by bootstrap values in the nodes and branch lengths are in scale.

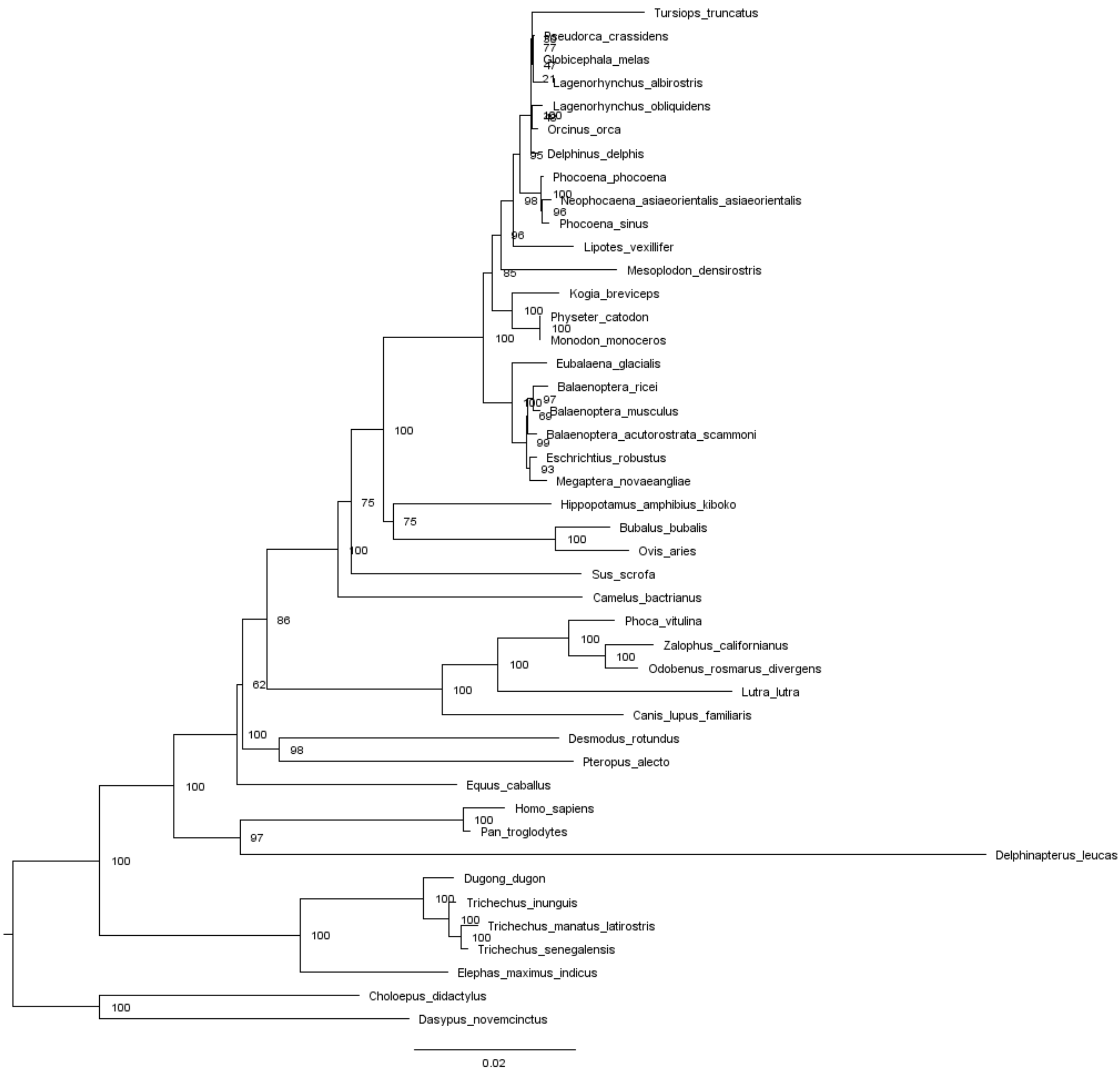

Figure S18. Maximum likelihood phylogenetic tree reconstructed with IQ-TREE 2 using the nucleotide alignment of TRPM3 gene. Branch confidence is determined by bootstrap values in the nodes and branch lengths are in scale.

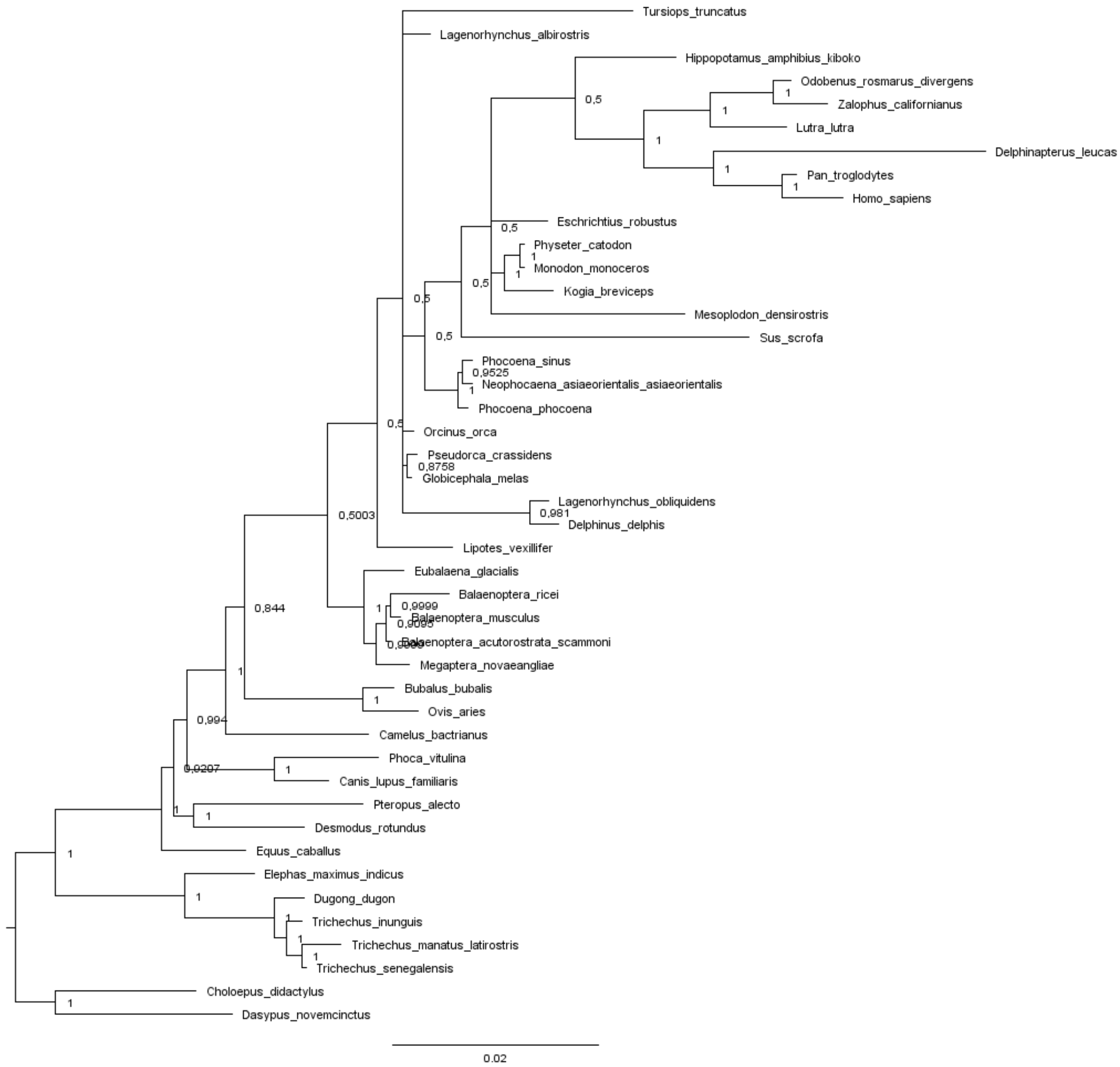

Figure S19. Bayesian phylogenetic tree reconstructed with MrBayes v. 3.2.7a using PartitionFinder v. 2.1.1 evolutionary models of nucleotide alignment of TRPM3 gene. Support values for the branches are given by posterior probabilities in the nodes. Branch lengths are in scale.

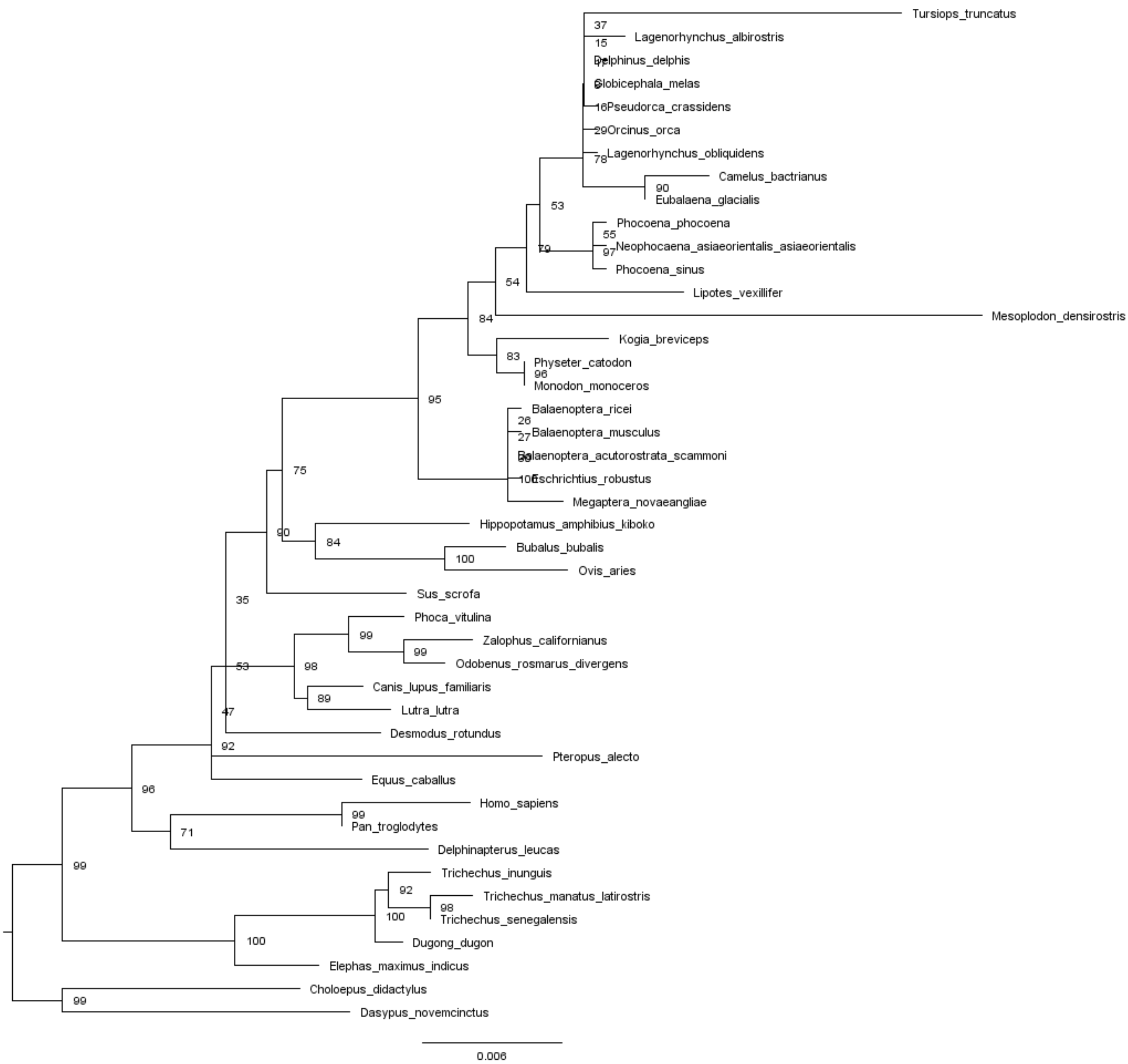

Figure S20. Maximum likelihood phylogenetic tree reconstructed with IQ-TREE 2 using the amino acid alignment of TRPM3 gene. Branch confidence is determined by bootstrap values in the nodes and branch lengths are in scale.

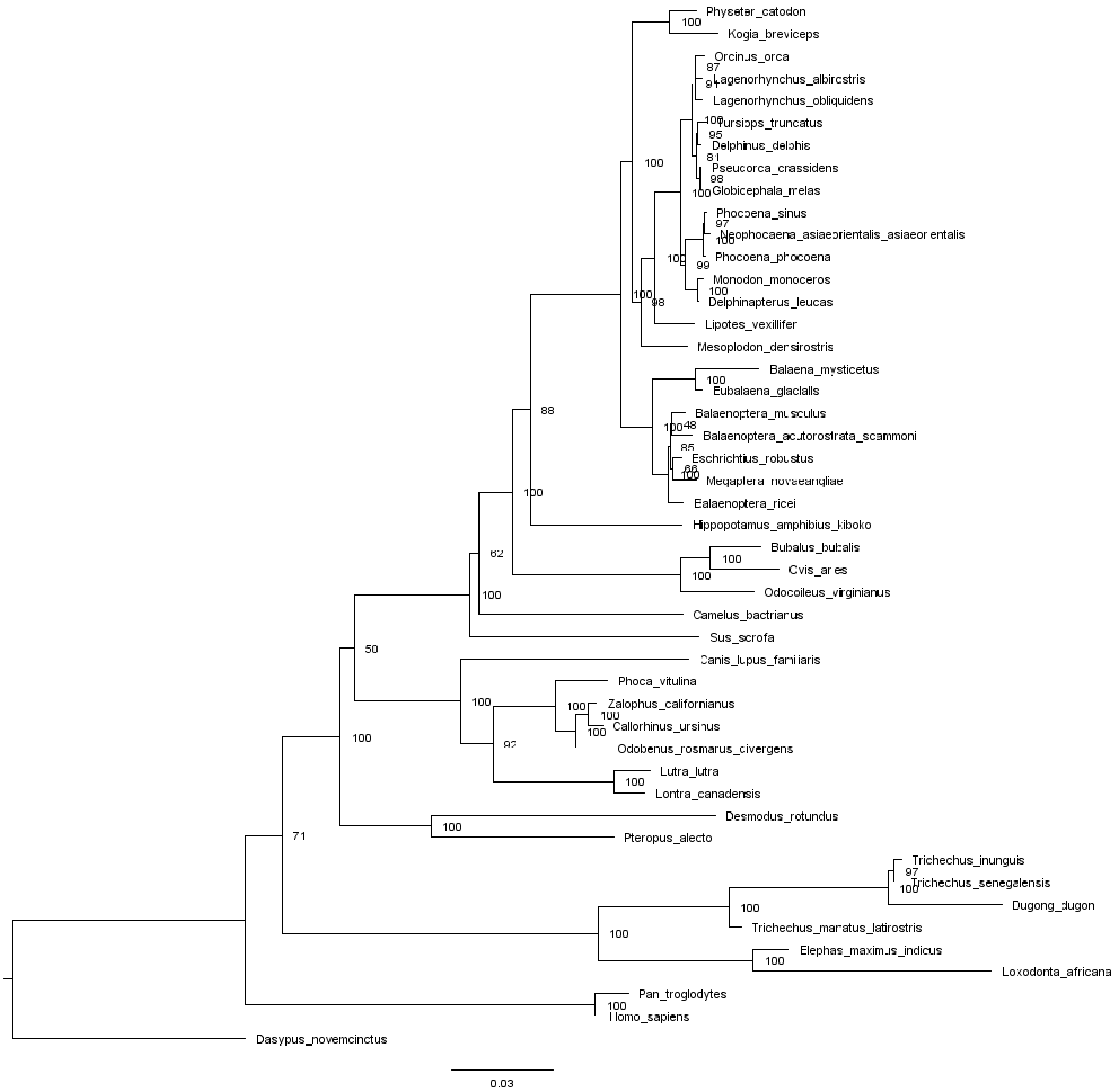

Figure S21. Maximum likelihood phylogenetic tree reconstructed with IQ-TREE 2 using the nucleotide alignment of TRPM4 gene. Branch confidence is determined by bootstrap values in the nodes and branch lengths are in scale.

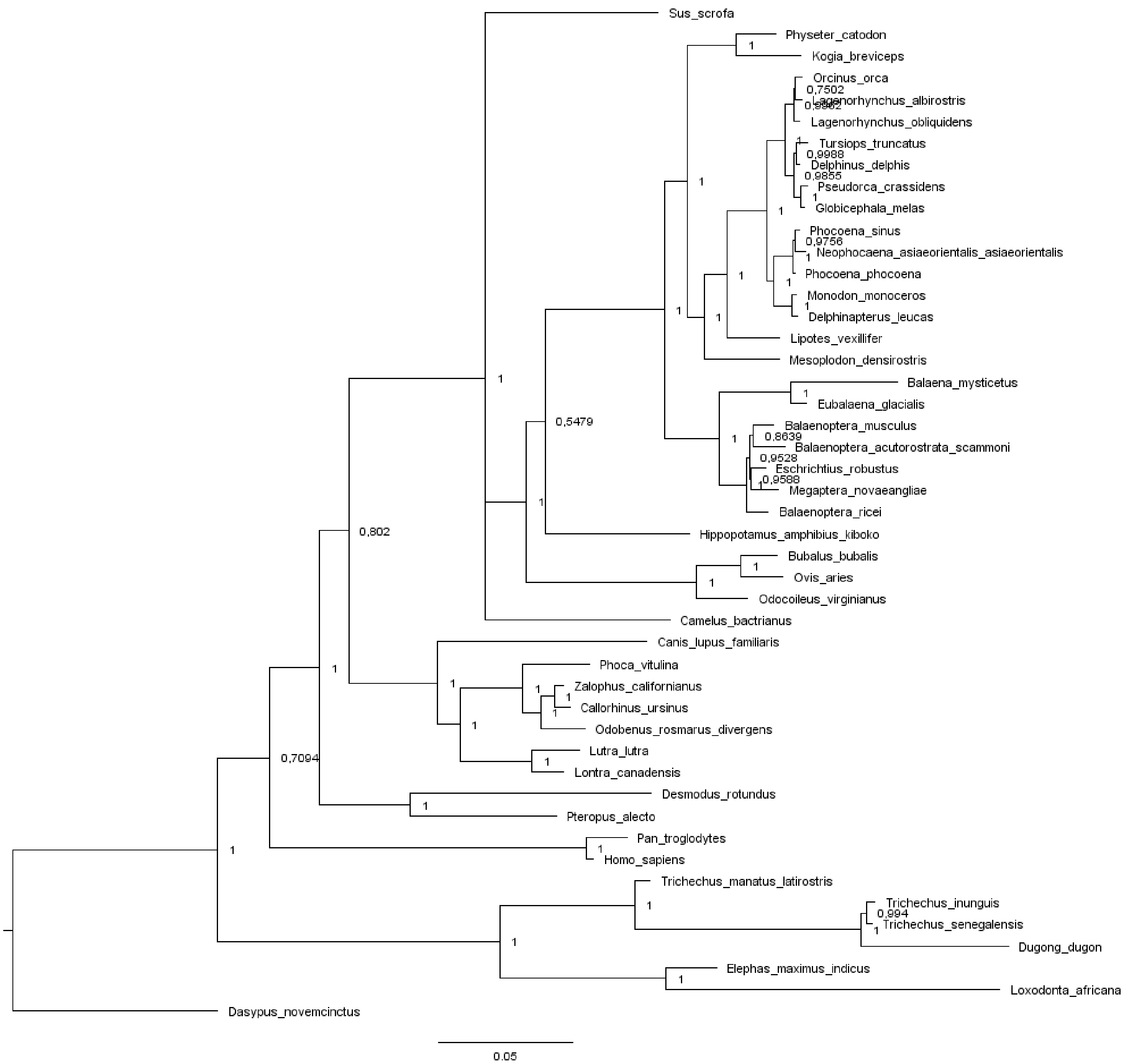

Figure S22. Bayesian phylogenetic tree reconstructed with MrBayes v. 3.2.7a using PartitionFinder v. 2.1.1 evolutionary models of nucleotide alignment of TRPM4 gene. Support values for the branches are given by posterior probabilities in the nodes. Branch lengths are in scale.

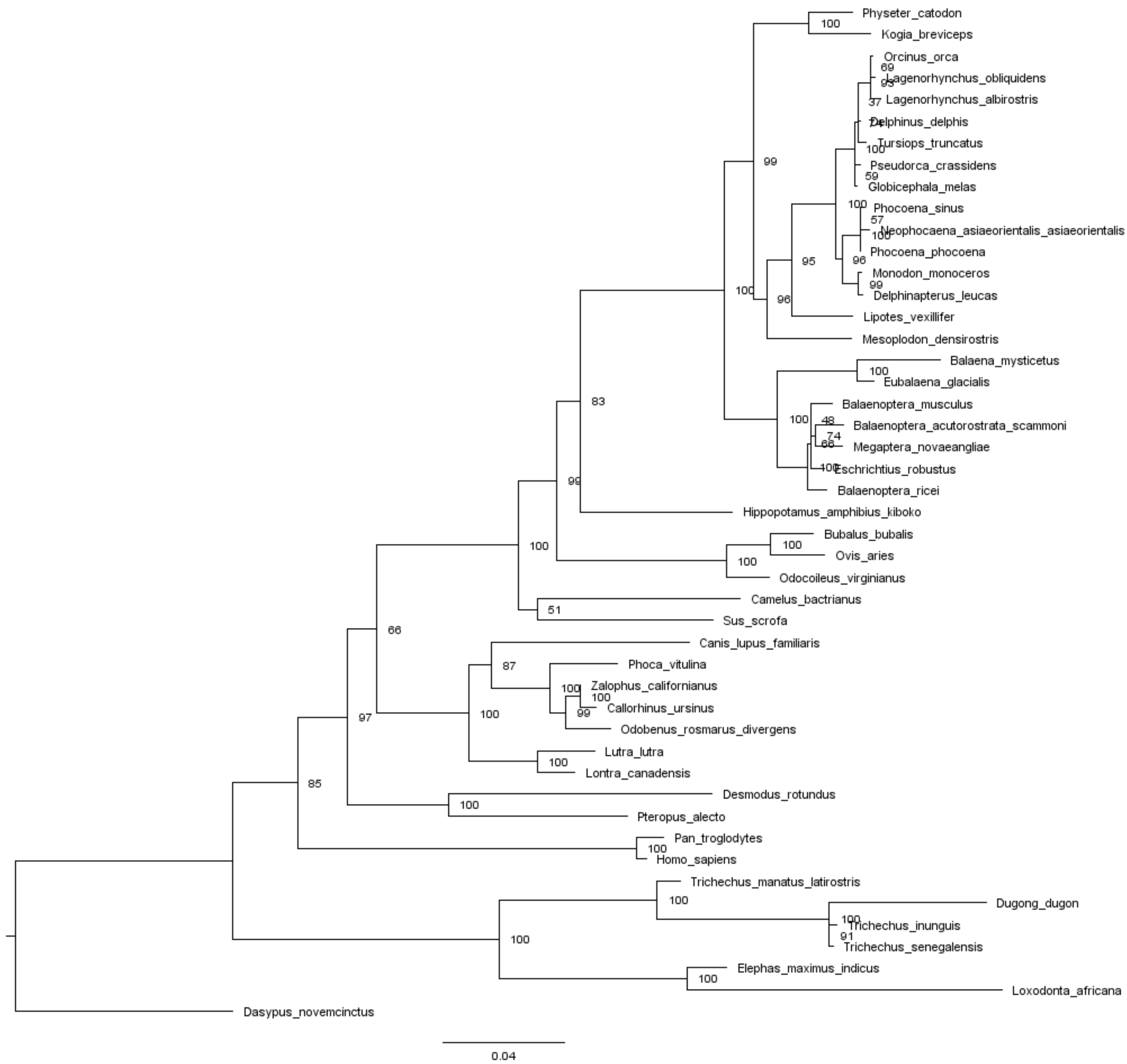

Figure S23. Maximum likelihood phylogenetic tree reconstructed with IQ-TREE 2 using the amino acid alignment of TRPM4 gene. Branch confidence is determined by bootstrap values in the nodes and branch lengths are in scale.

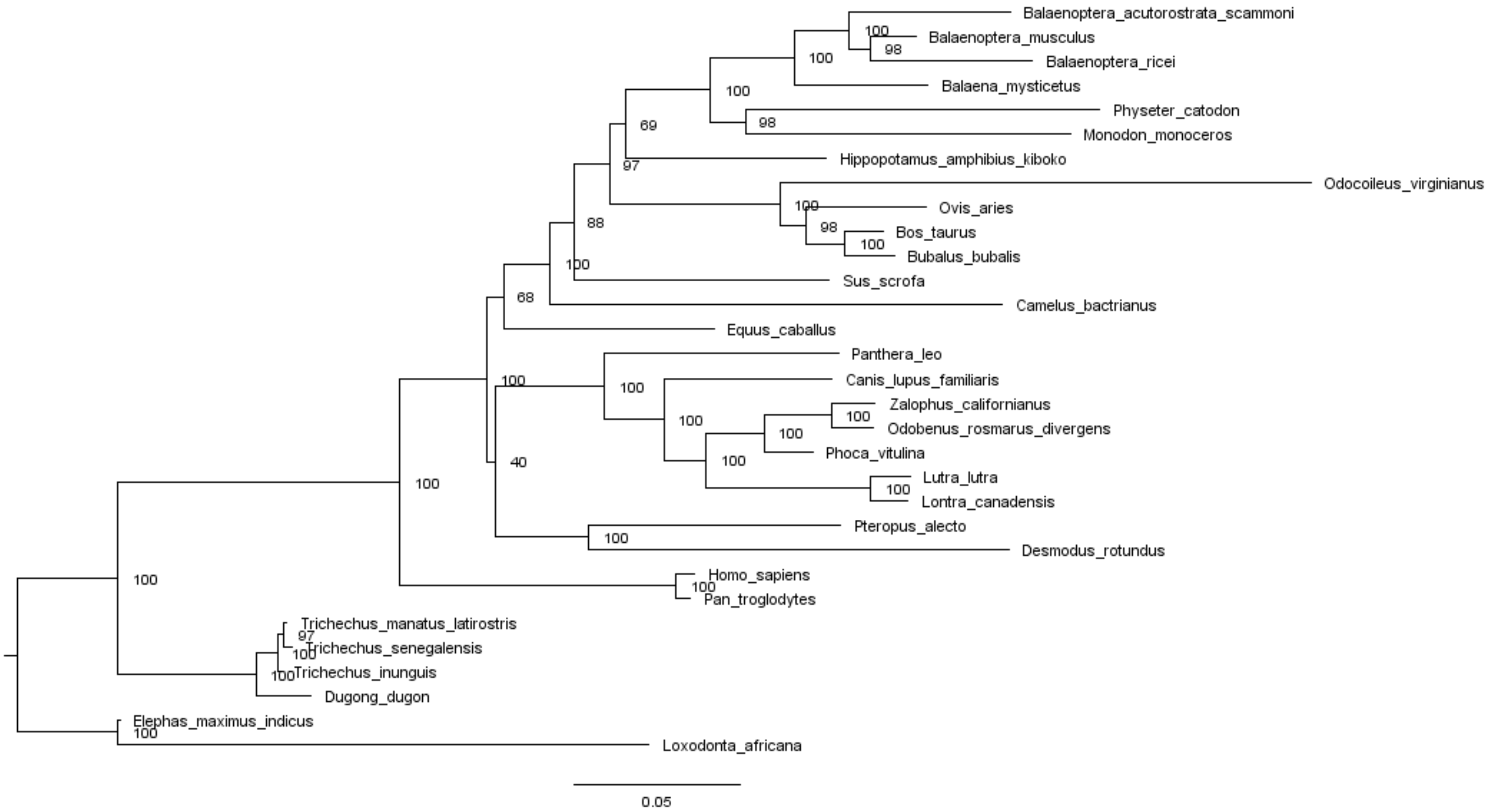

Figure S24. Maximum likelihood phylogenetic tree reconstructed with IQ-TREE 2 using the nucleotide alignment of TRPM5 gene without sequences with evidence of inactivation. Branch confidence is determined by bootstrap values in the nodes and branch lengths are in scale.

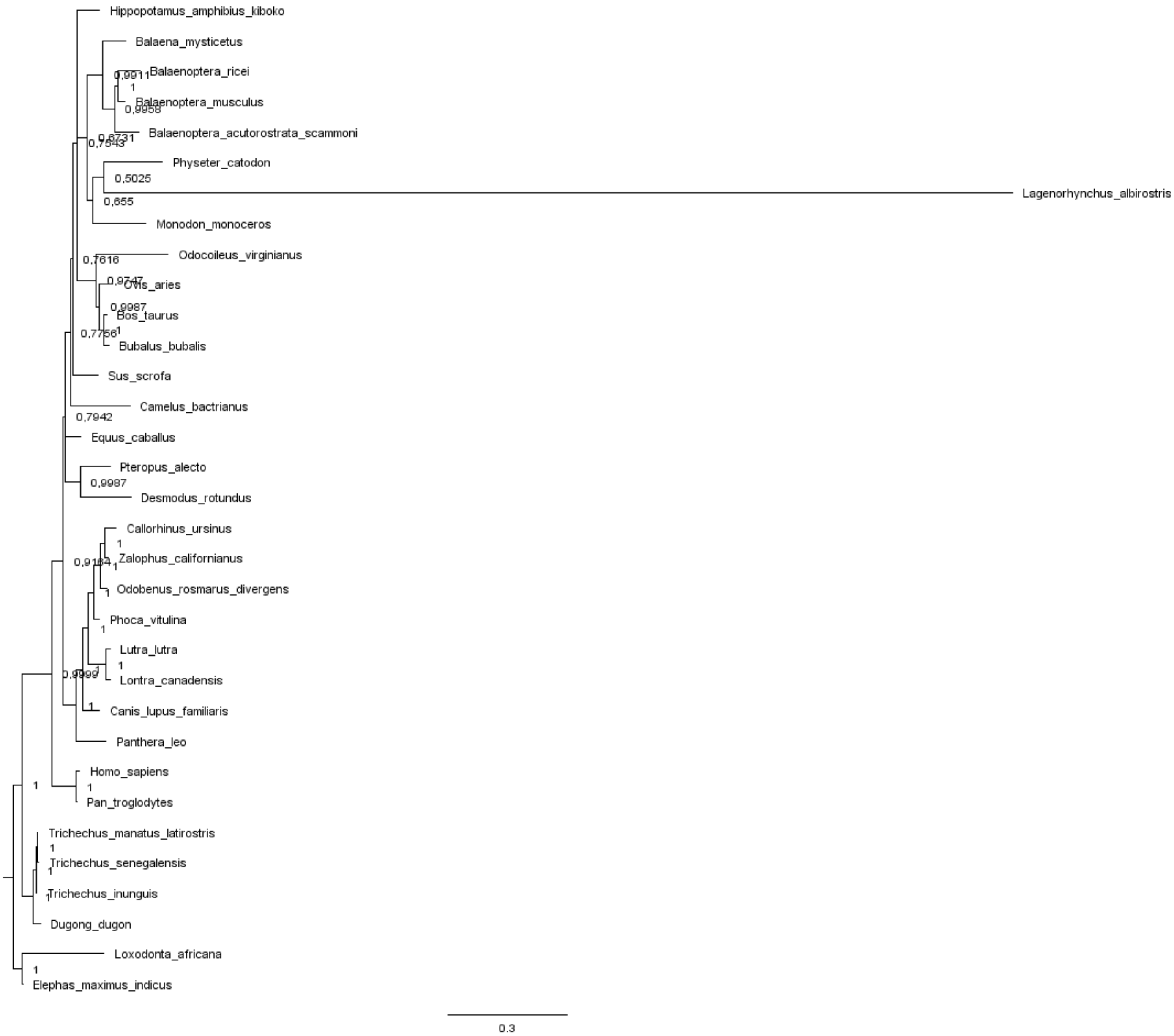

Figure S25. Bayesian phylogenetic tree reconstructed with MrBayes v. 3.2.7a using PartitionFinder v. 2.1.1 evolutionary models of nucleotide alignment of TRPM5 gene without sequences with evidence of inactivation. Support values for the branches are given by posterior probabilities in the nodes. Branch lengths are in scale.

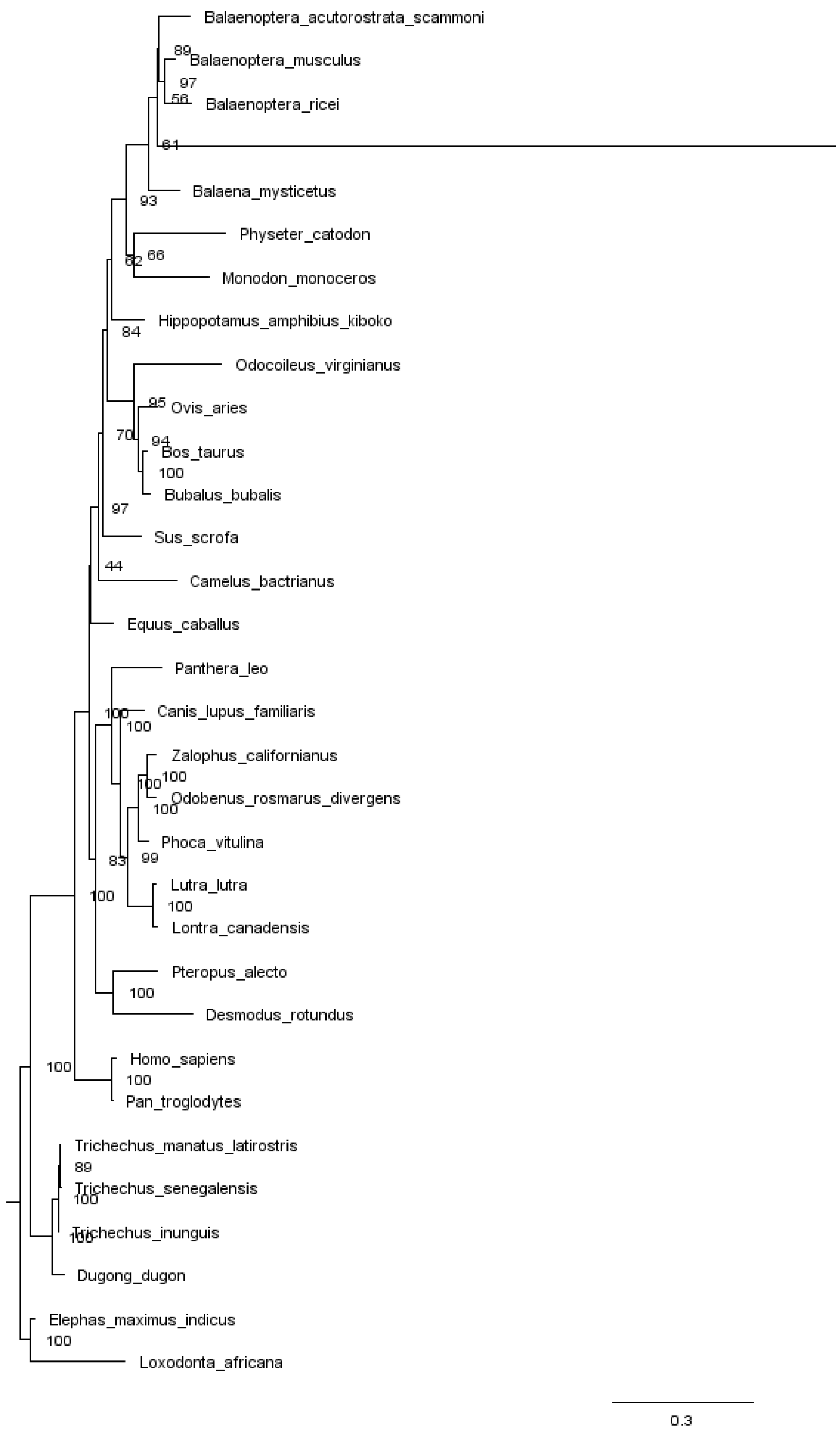

Figure S26. Maximum likelihood phylogenetic tree reconstructed with IQ-TREE 2 using the amino acid alignment of TRPM5 gene without sequences with evidence of inactivation. Branch confidence is determined by bootstrap values in the nodes and branch lengths are in scale.

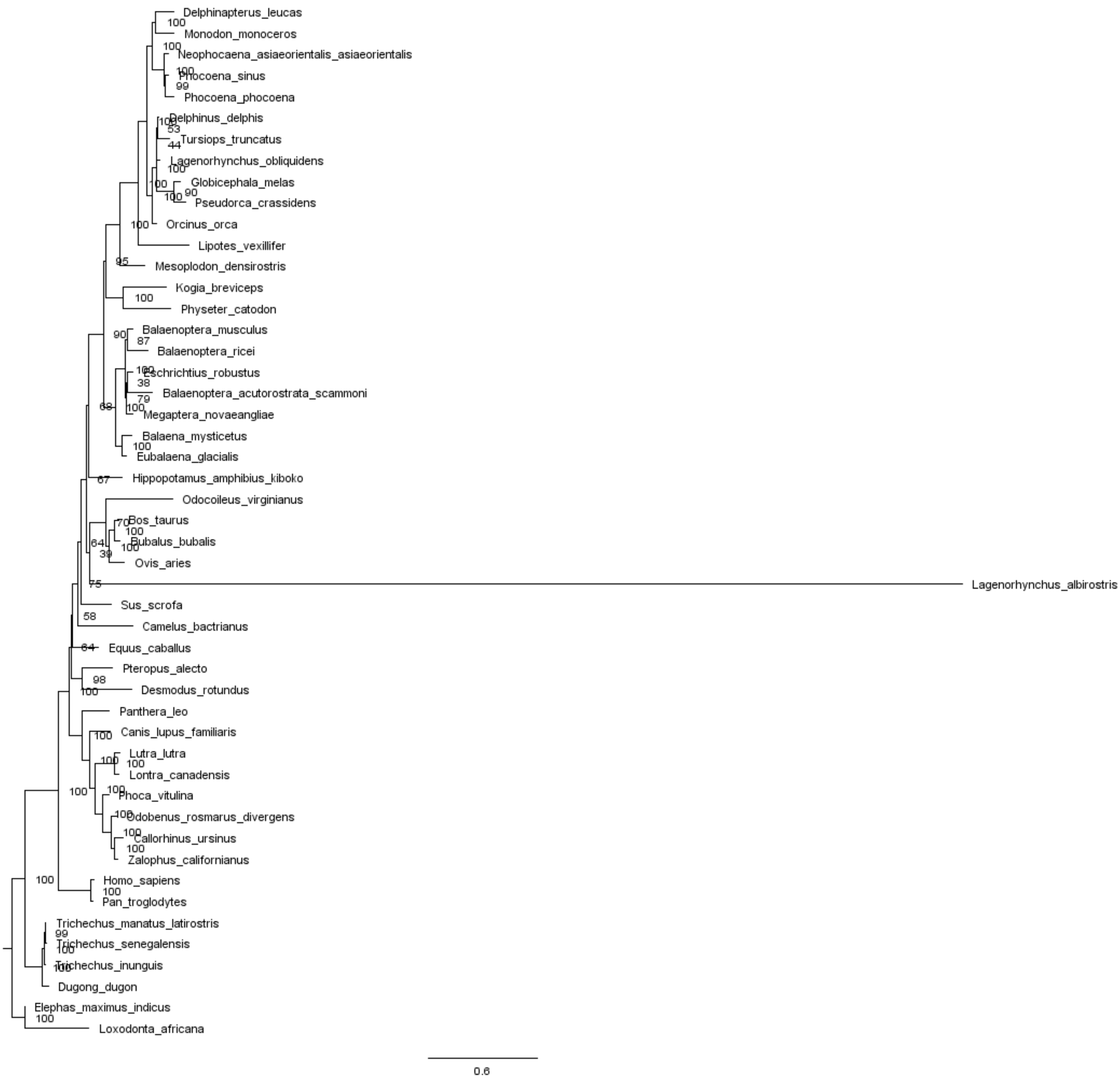

Figure S27. Maximum likelihood phylogenetic tree reconstructed with IQ-TREE 2 using the nucleotide alignment of TRPM5 gene with the full dataset. Branch confidence is determined by bootstrap values in the nodes and branch lengths are in scale.

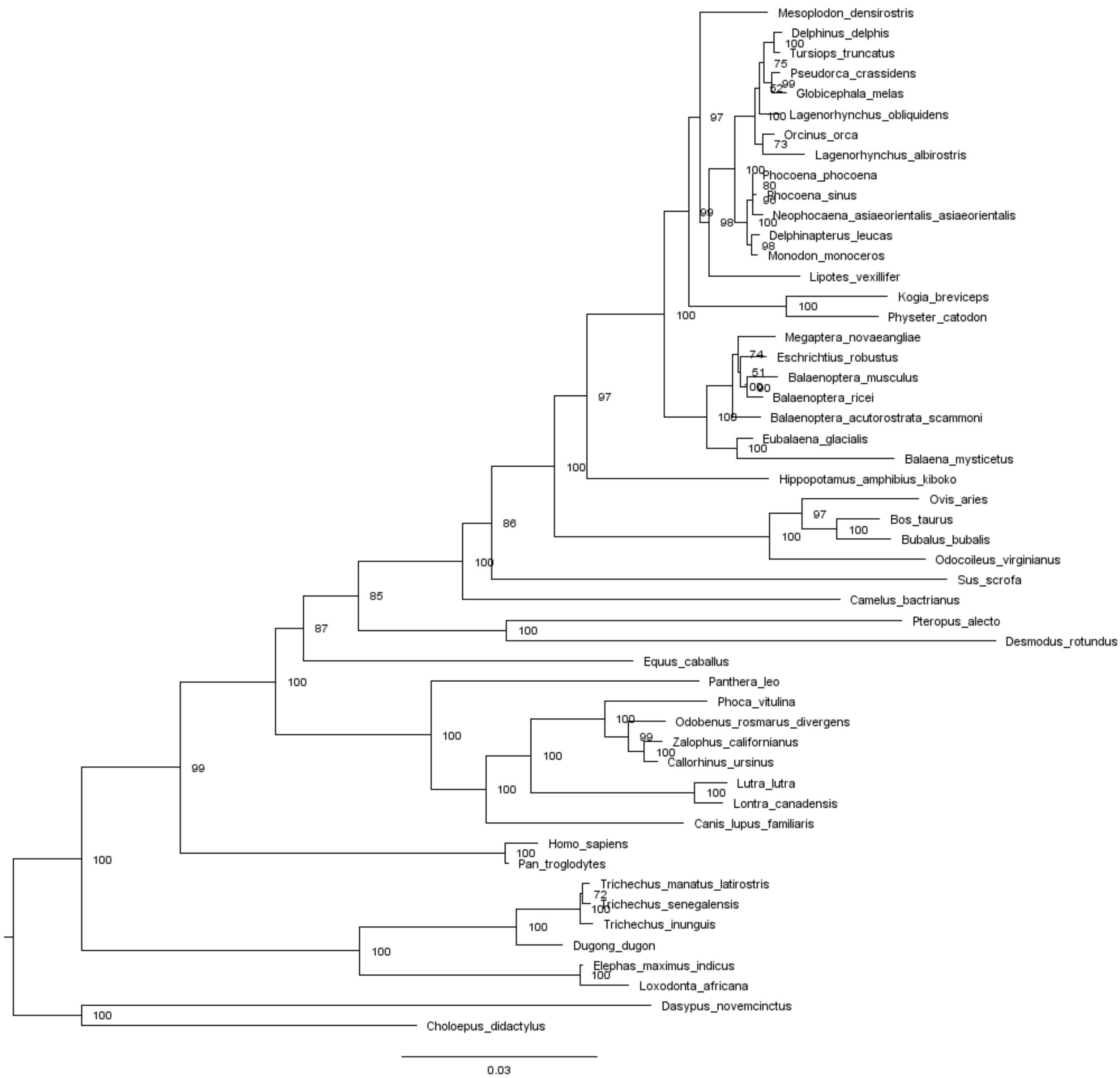

Figure S28. Maximum likelihood phylogenetic tree reconstructed with IQ-TREE 2 using the nucleotide alignment of TRPM8 gene. Branch confidence is determined by bootstrap values in the nodes and branch lengths are in scale.

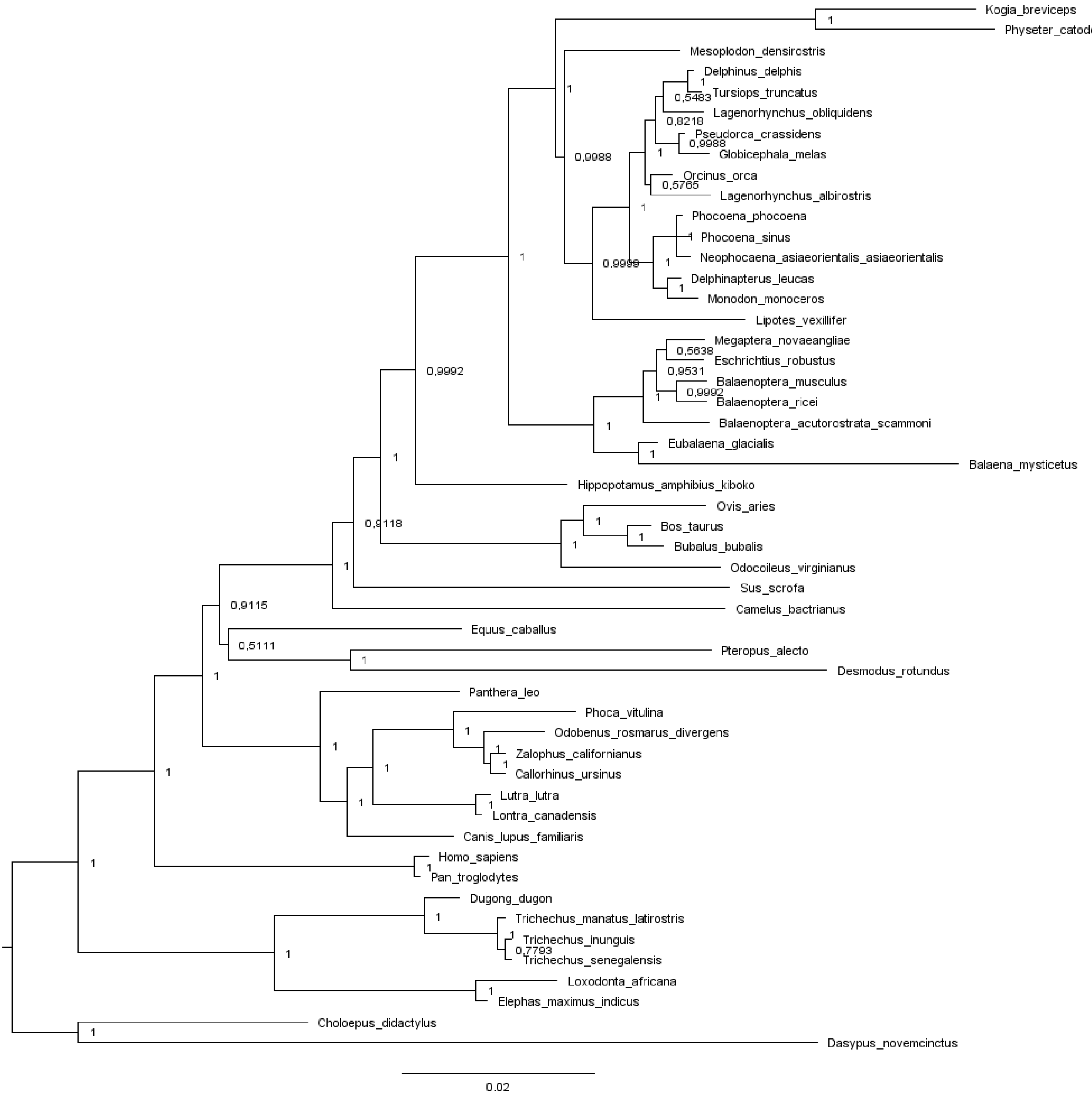

Figure S29. Bayesian phylogenetic tree reconstructed with MrBayes v. 3.2.7a using PartitionFinder v. 2.1.1 evolutionary models of nucleotide alignment of TRPM8 gene. Support values for the branches are given by posterior probabilities in the nodes. Branch lengths are in scale.

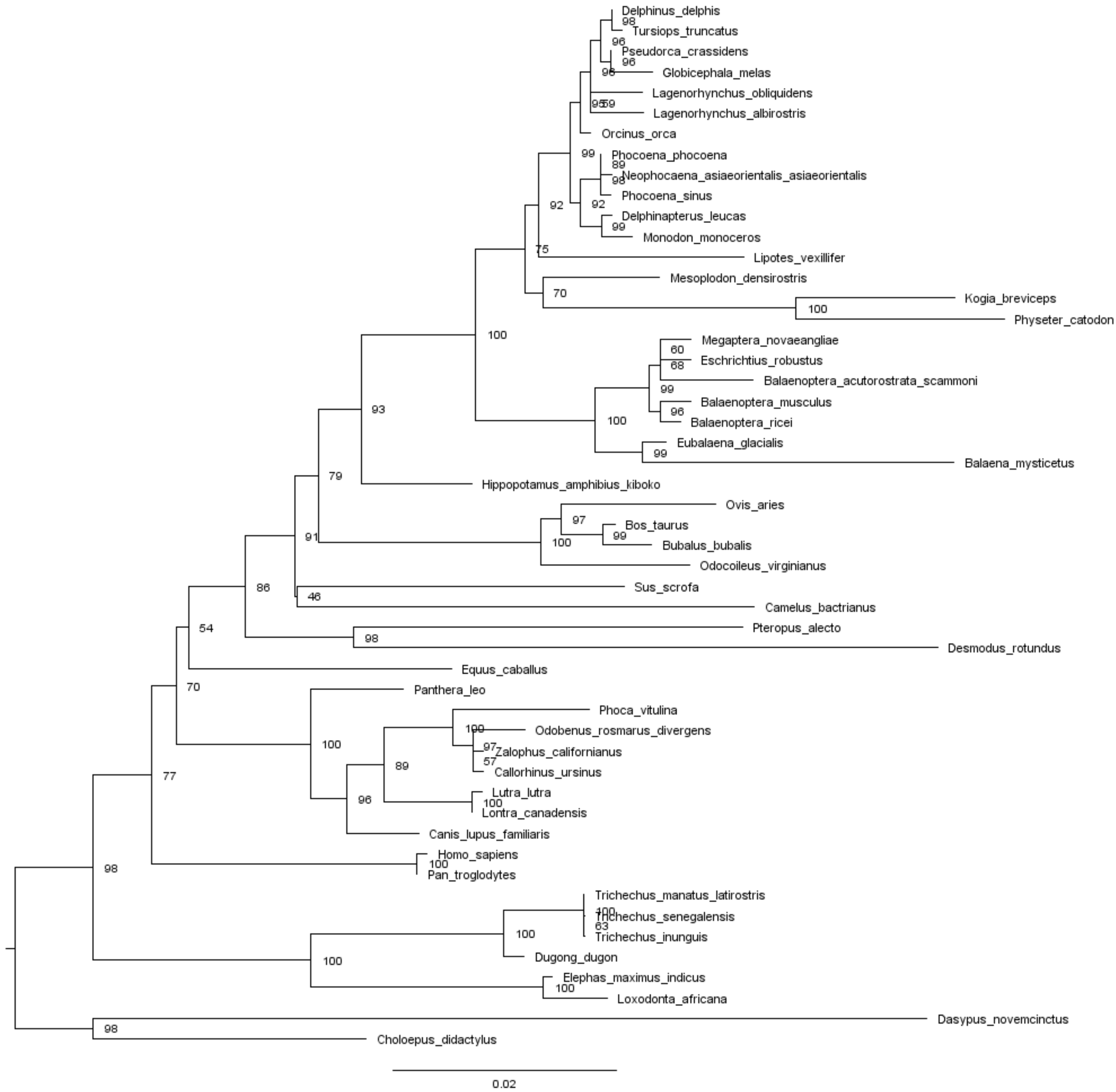

Figure S30. Maximum likelihood phylogenetic tree reconstructed with IQ-TREE 2 using the amino acid alignment of TRPM8 gene. Branch confidence is determined by bootstrap values in the nodes and branch lengths are in scale.

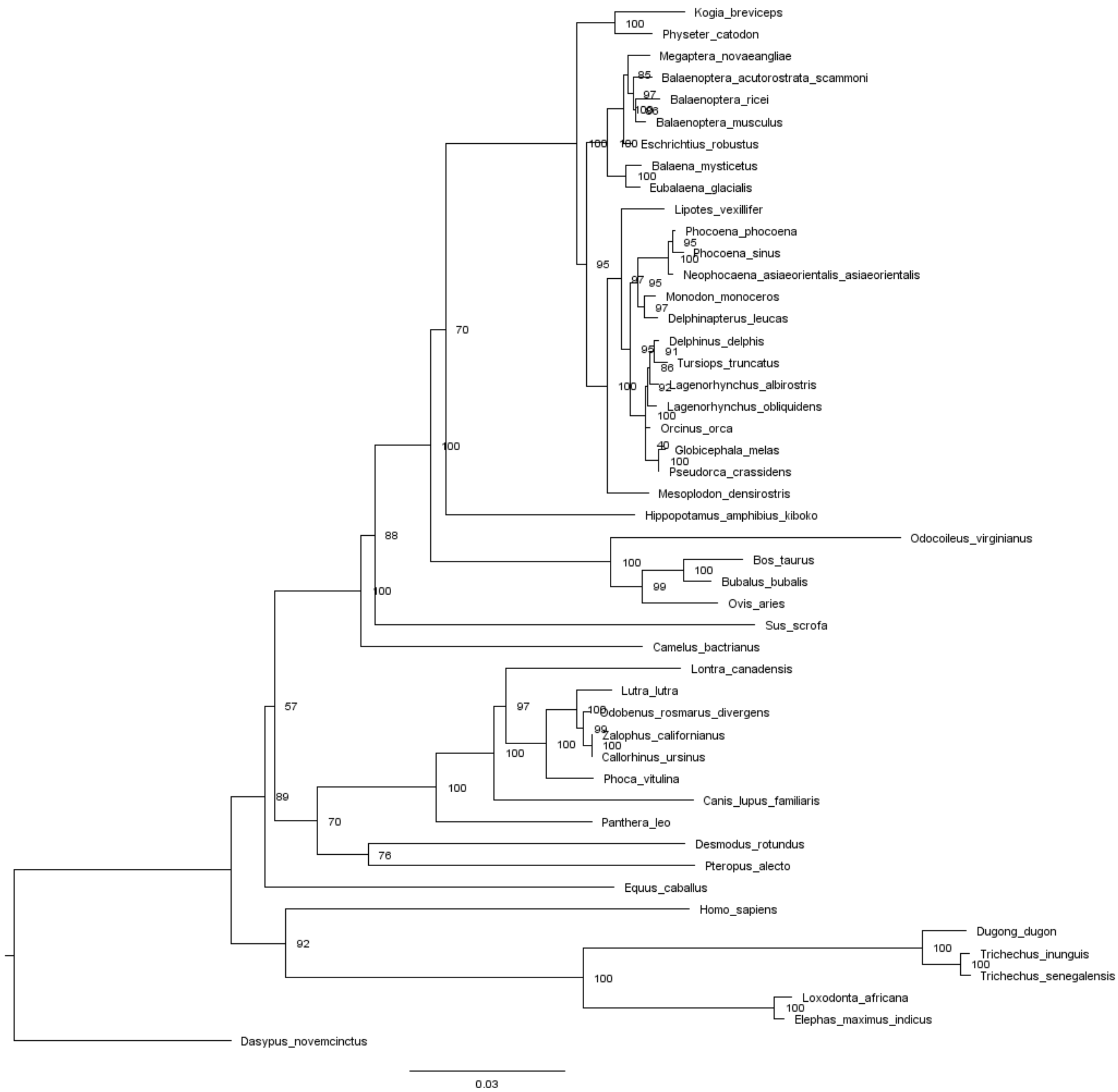

Figure S31. Maximum likelihood phylogenetic tree reconstructed with IQ-TREE 2 using the nucleotide alignment of TRPV1 gene. Branch confidence is determined by bootstrap values in the nodes and branch lengths are in scale.

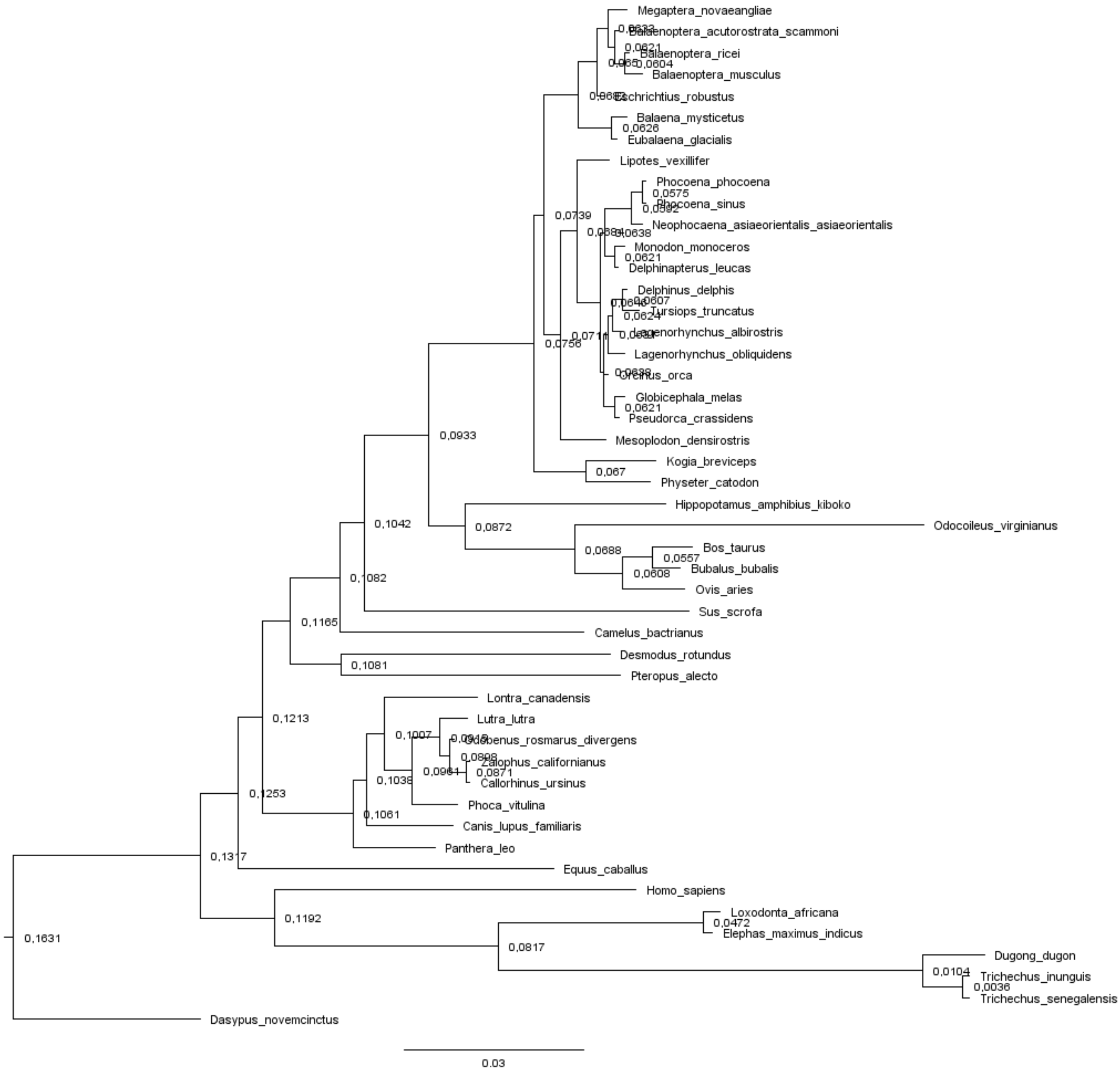

Figure S32. Bayesian phylogenetic tree reconstructed with MrBayes v. 3.2.7a using PartitionFinder v. 2.1.1 evolutionary models of nucleotide alignment of TRPV1 gene. Support values for the branches are given by posterior probabilities in the nodes. Branch lengths are in scale.

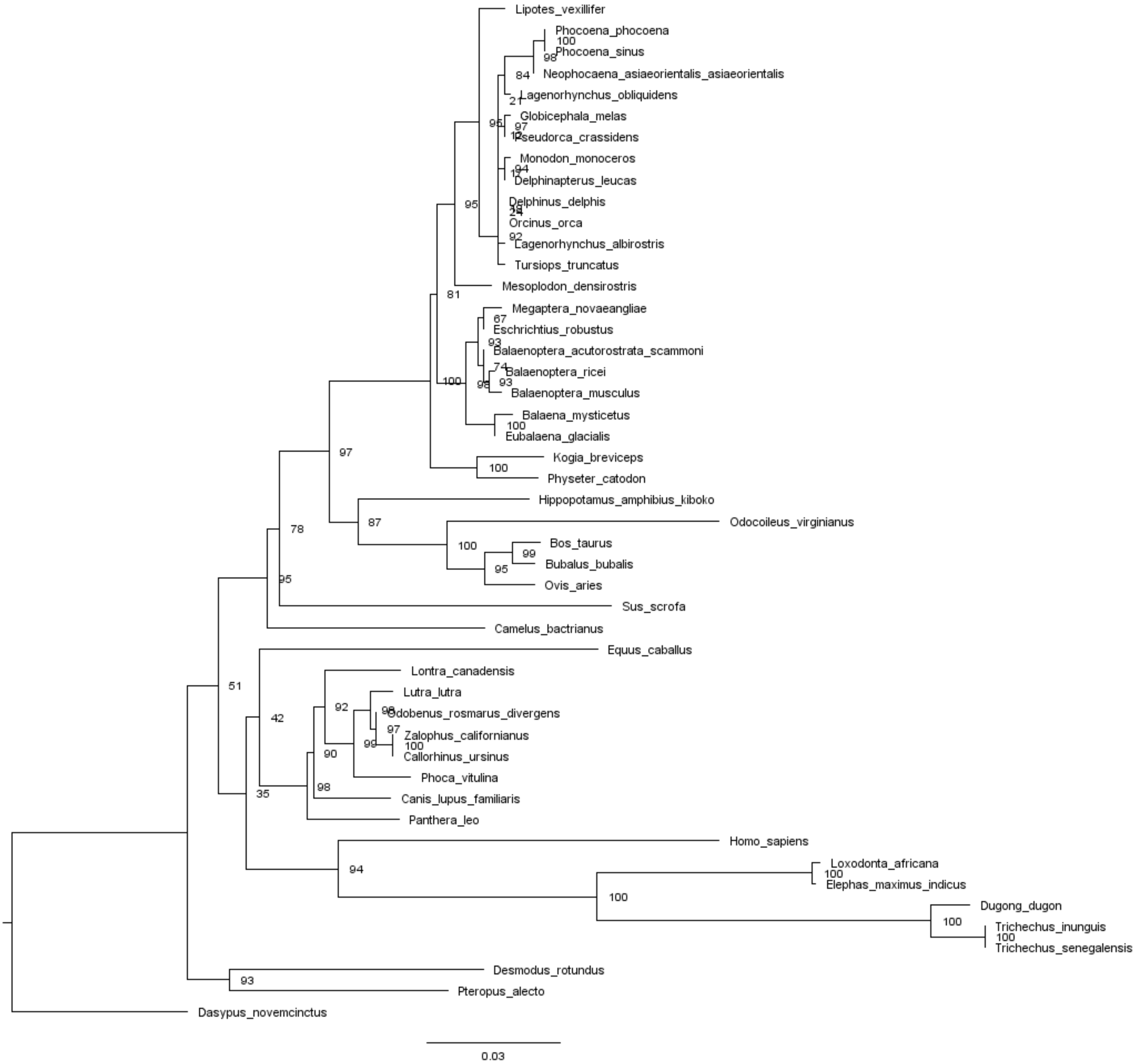

Figure S33. Maximum likelihood phylogenetic tree reconstructed with IQ-TREE 2 using the amino acid alignment of TRPV1 gene. Branch confidence is determined by bootstrap values in the nodes and branch lengths are in scale.

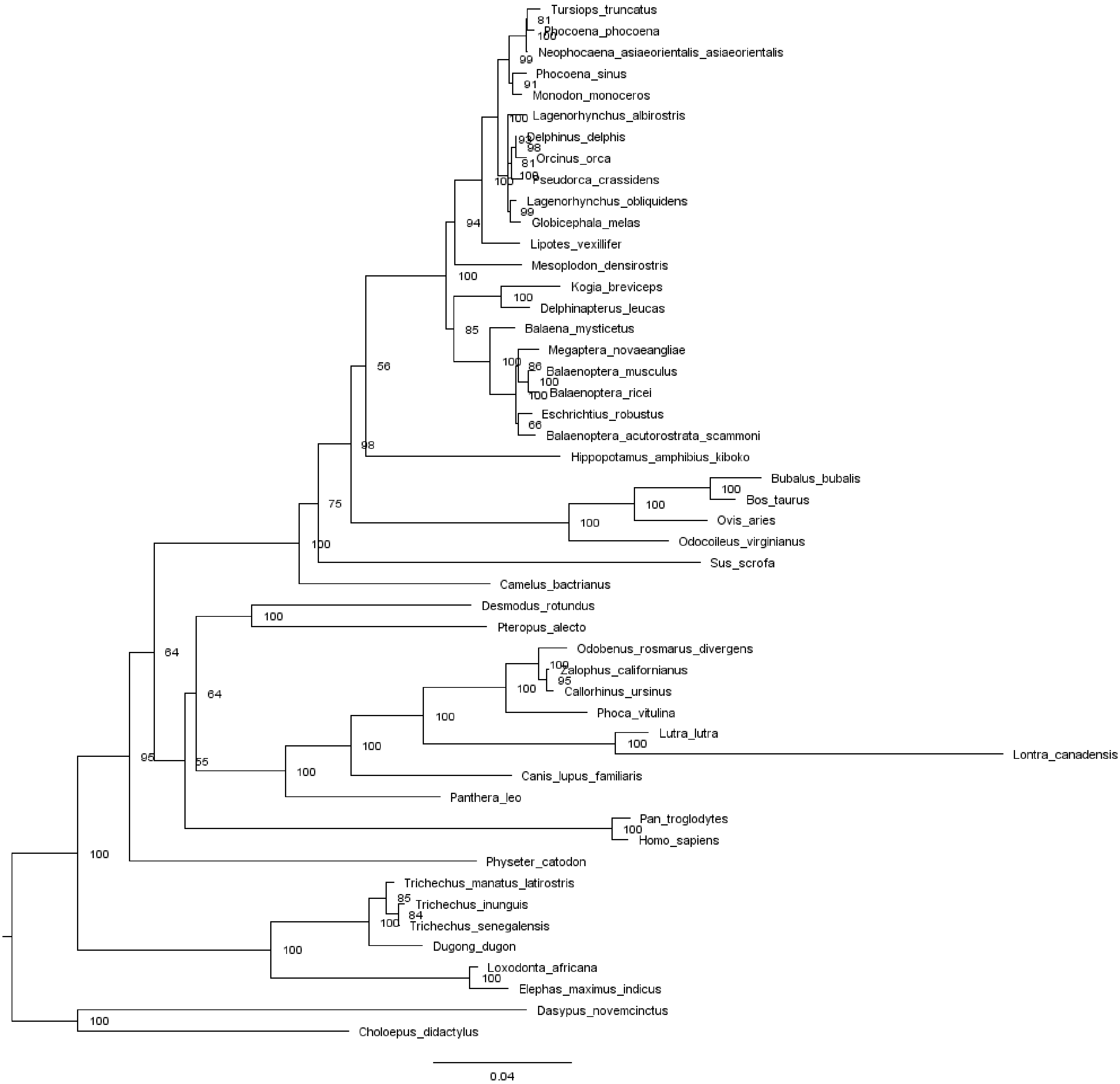

Figure S34. Maximum likelihood phylogenetic tree reconstructed with IQ-TREE 2 using the nucleotide alignment of TRPV2 gene. Branch confidence is determined by bootstrap values in the nodes and branch lengths are in scale.

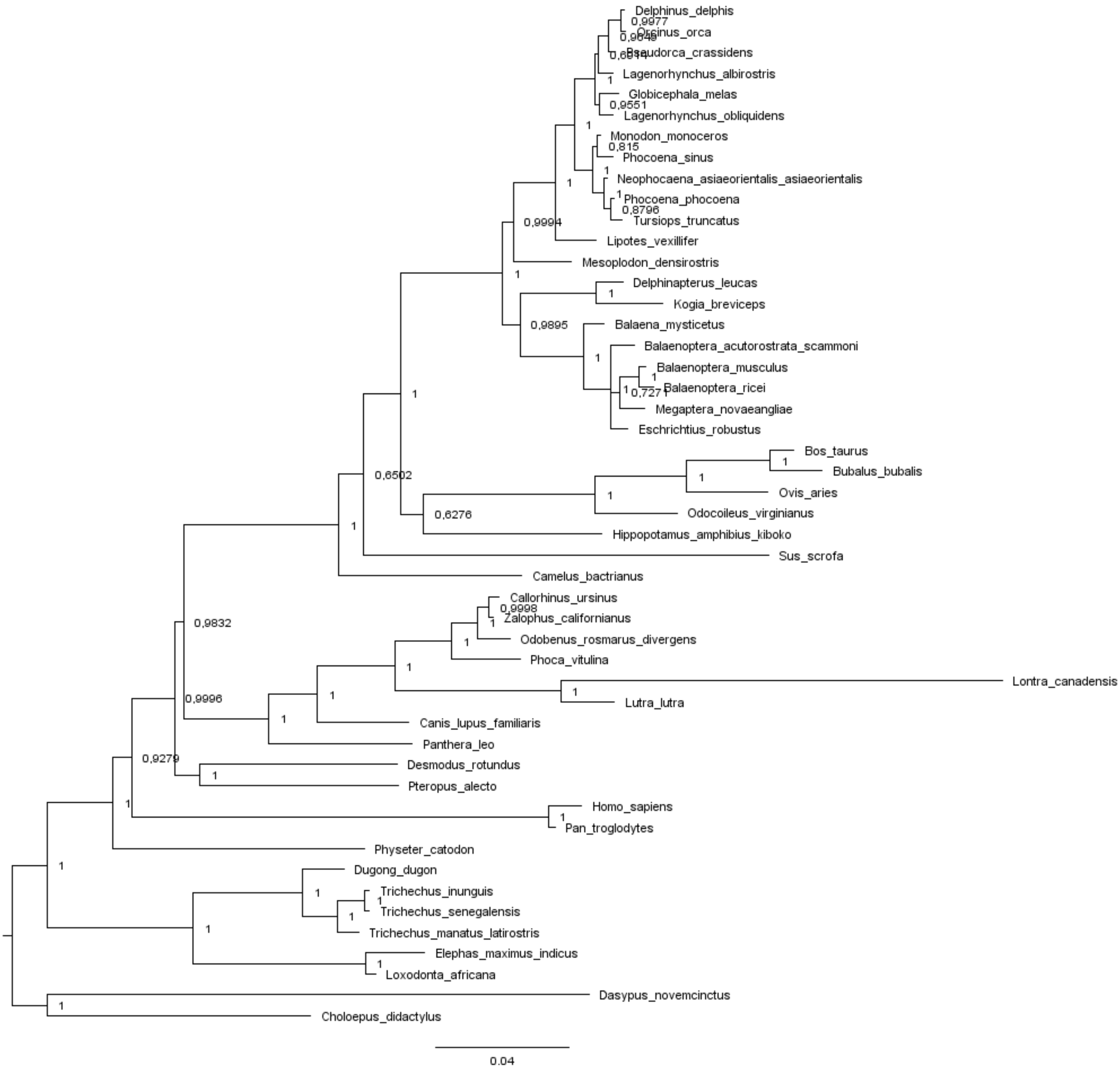

Figure S35. Bayesian phylogenetic tree reconstructed with MrBayes v. 3.2.7a using PartitionFinder v. 2.1.1 evolutionary models of nucleotide alignment of TRPV2 gene. Support values for the branches are given by posterior probabilities in the nodes. Branch lengths are in scale.

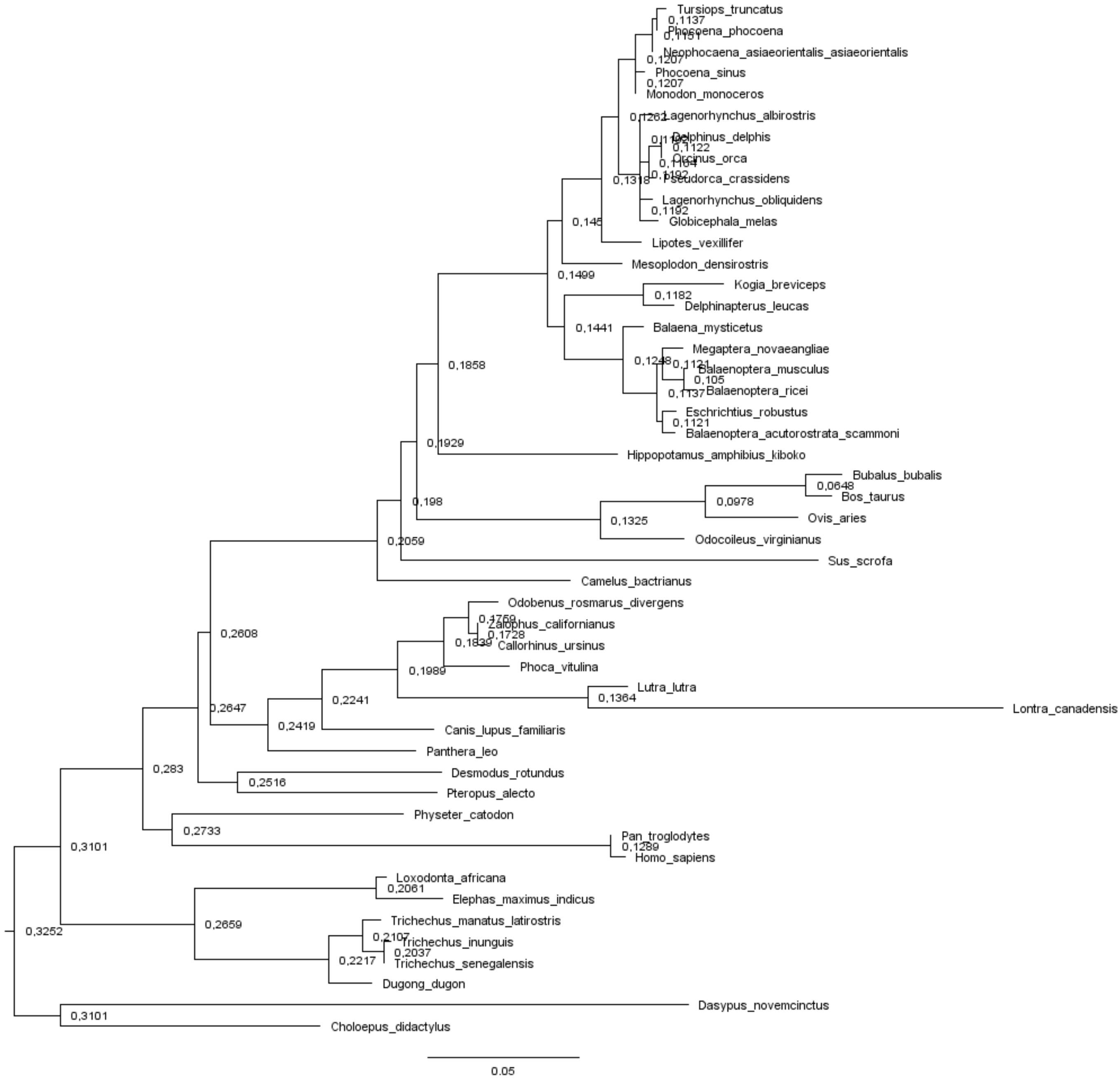

Figure S36. Maximum likelihood phylogenetic tree reconstructed with IQ-TREE 2 using the amino acid alignment of TRPV2 gene. Branch confidence is determined by bootstrap values in the nodes and branch lengths are in scale.

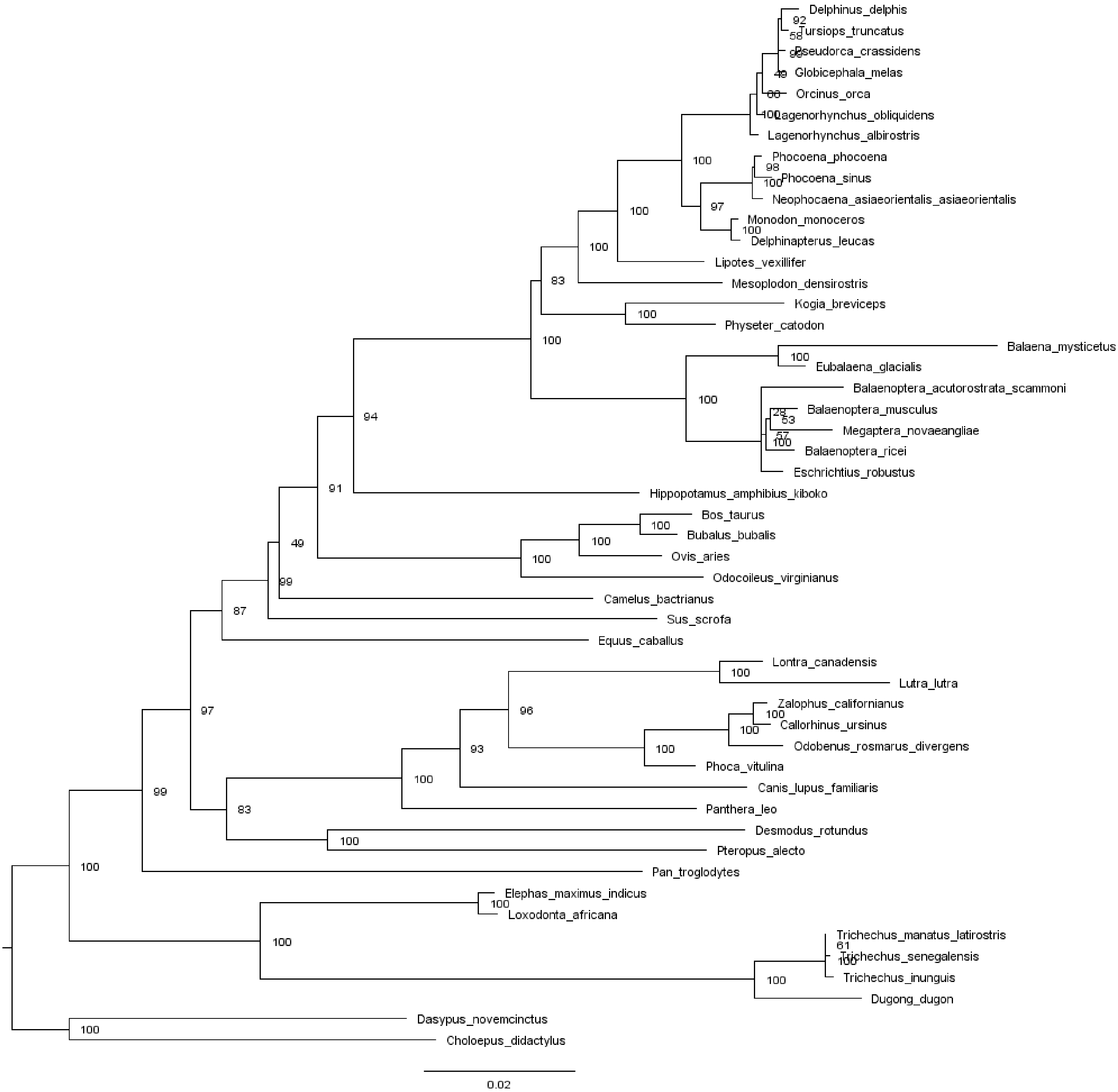

Figure S37. Maximum likelihood phylogenetic tree reconstructed with IQ-TREE 2 using the nucleotide alignment of TRPV3 gene. Branch confidence is determined by bootstrap values in the nodes and branch lengths are in scale.

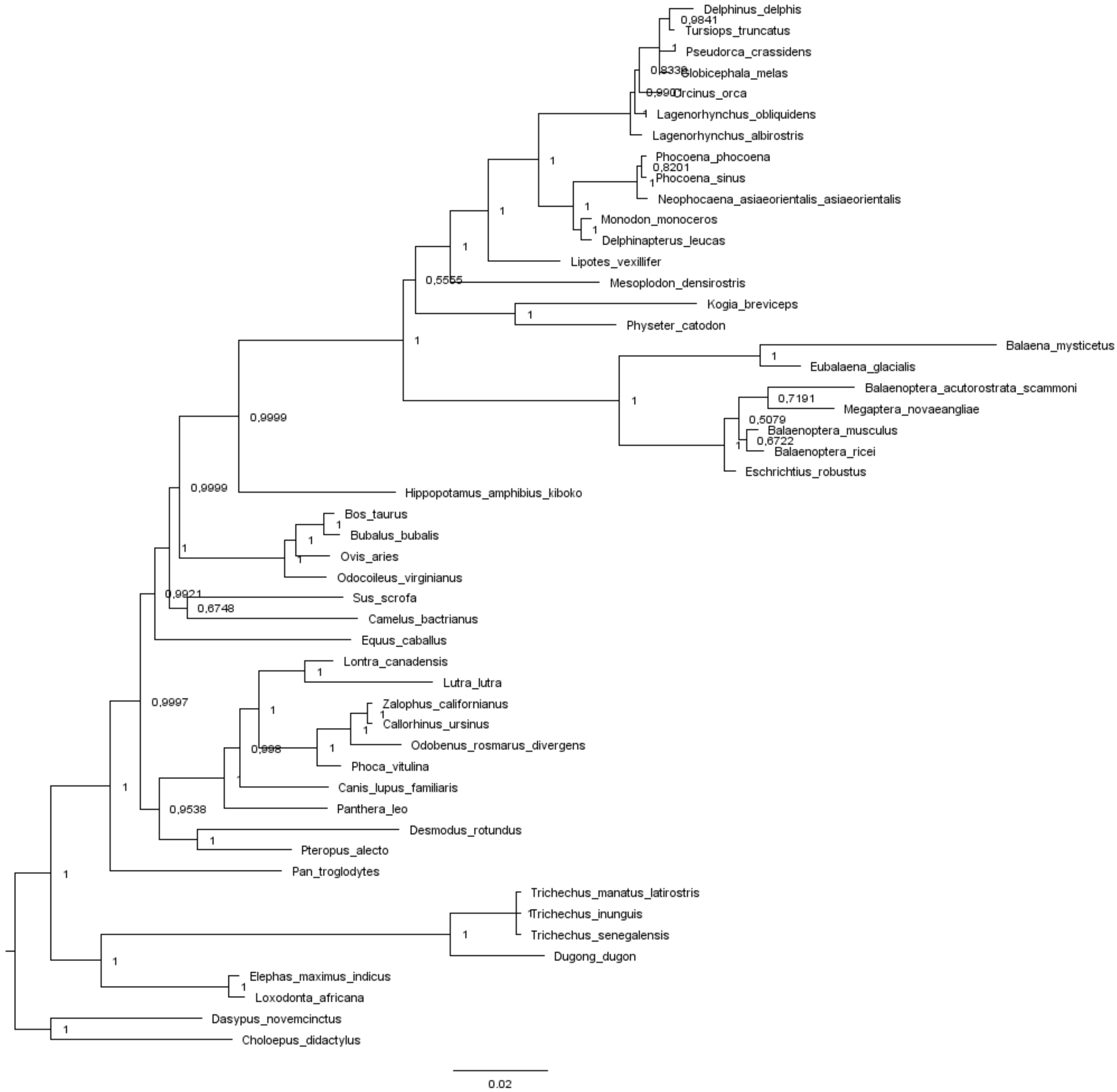

Figure S38. Bayesian phylogenetic tree reconstructed with MrBayes v. 3.2.7a using PartitionFinder v. 2.1.1 evolutionary models of nucleotide alignment of TRPV3 gene. Support values for the branches are given by posterior probabilities in the nodes. Branch lengths are in scale.

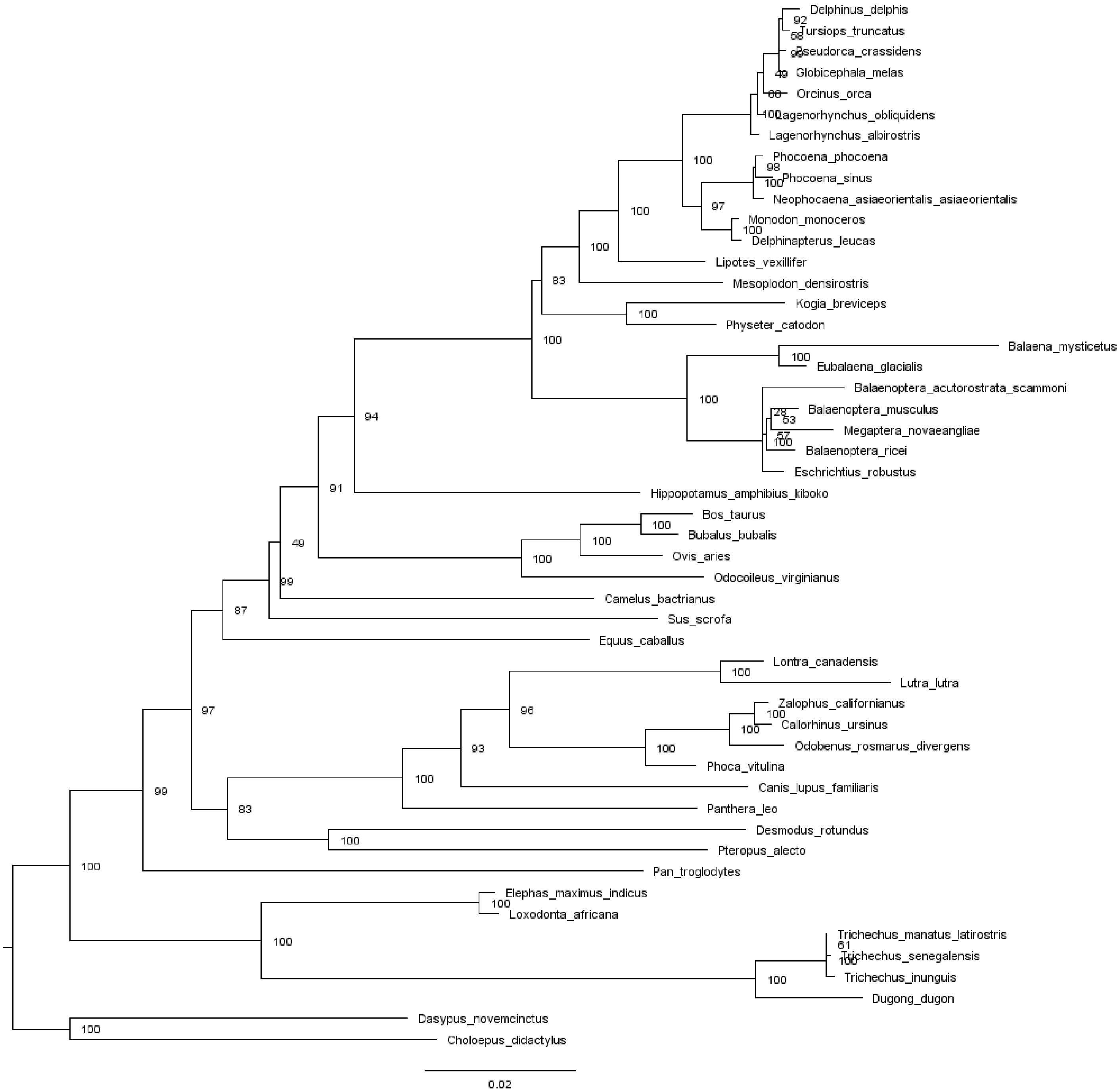

Figure S39. Maximum likelihood phylogenetic tree reconstructed with IQ-TREE 2 using the amino acid alignment of TRPV3 gene. Branch confidence is determined by bootstrap values in the nodes and branch lengths are in scale.

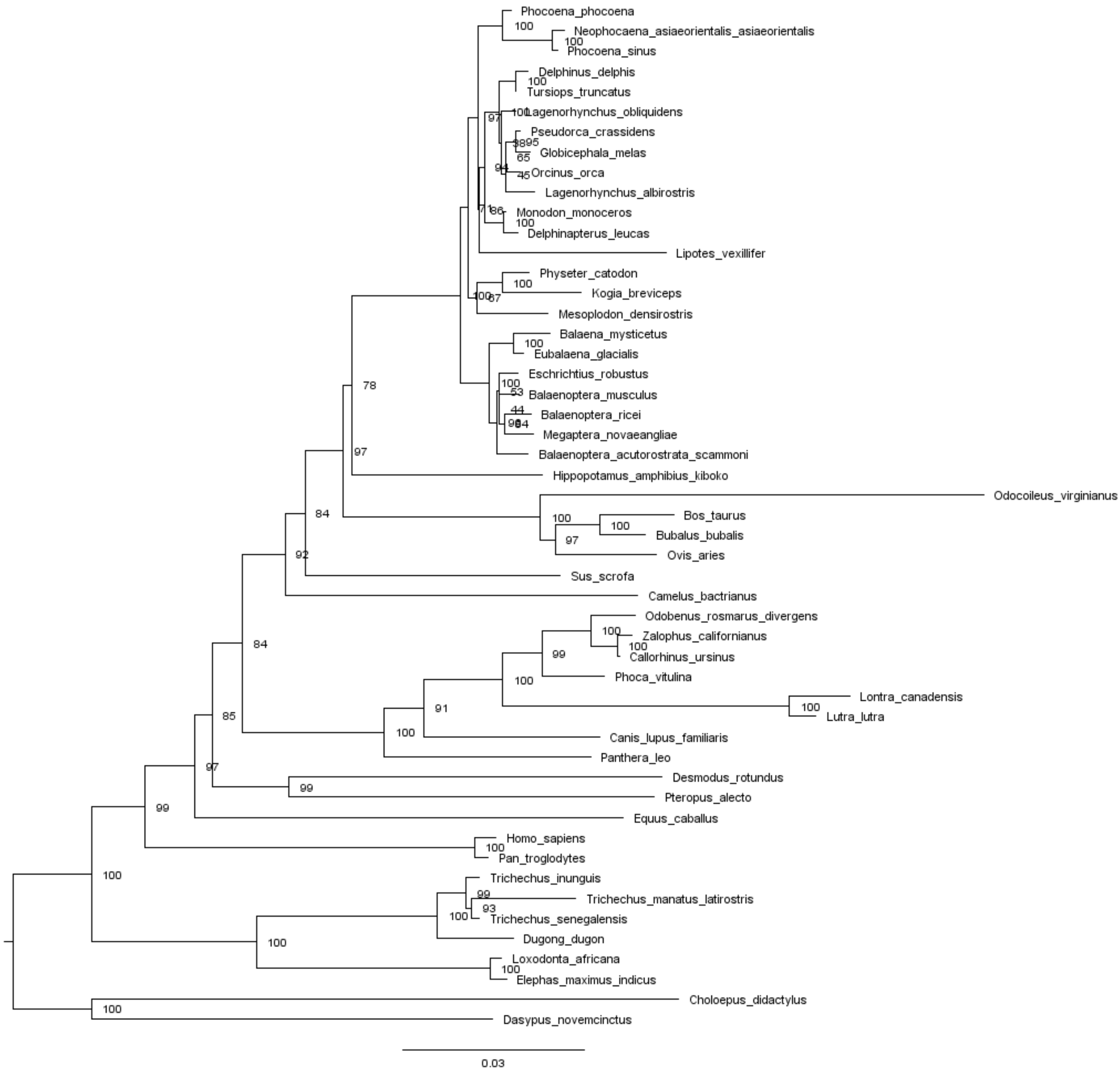

Figure S40. Maximum likelihood phylogenetic tree reconstructed with IQ-TREE 2 using the nucleotide alignment of TRPV4 gene. Branch confidence is determined by bootstrap values in the nodes and branch lengths are in scale.

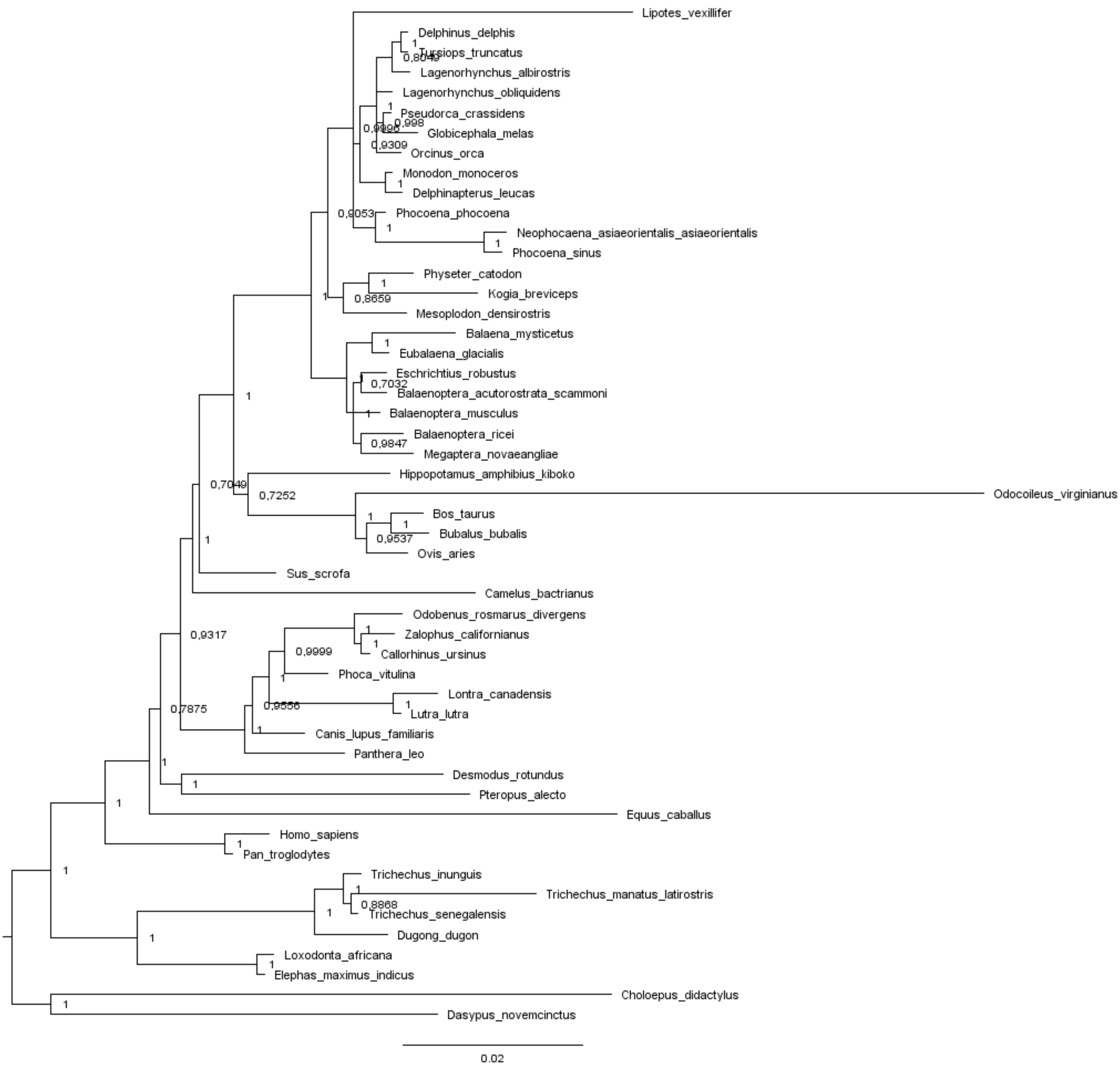

Figure S41. Bayesian phylogenetic tree reconstructed with MrBayes v. 3.2.7a using PartitionFinder v. 2.1.1 evolutionary models of nucleotide alignment of TRPV4 gene. Support values for the branches are given by posterior probabilities in the nodes. Branch lengths are in scale.

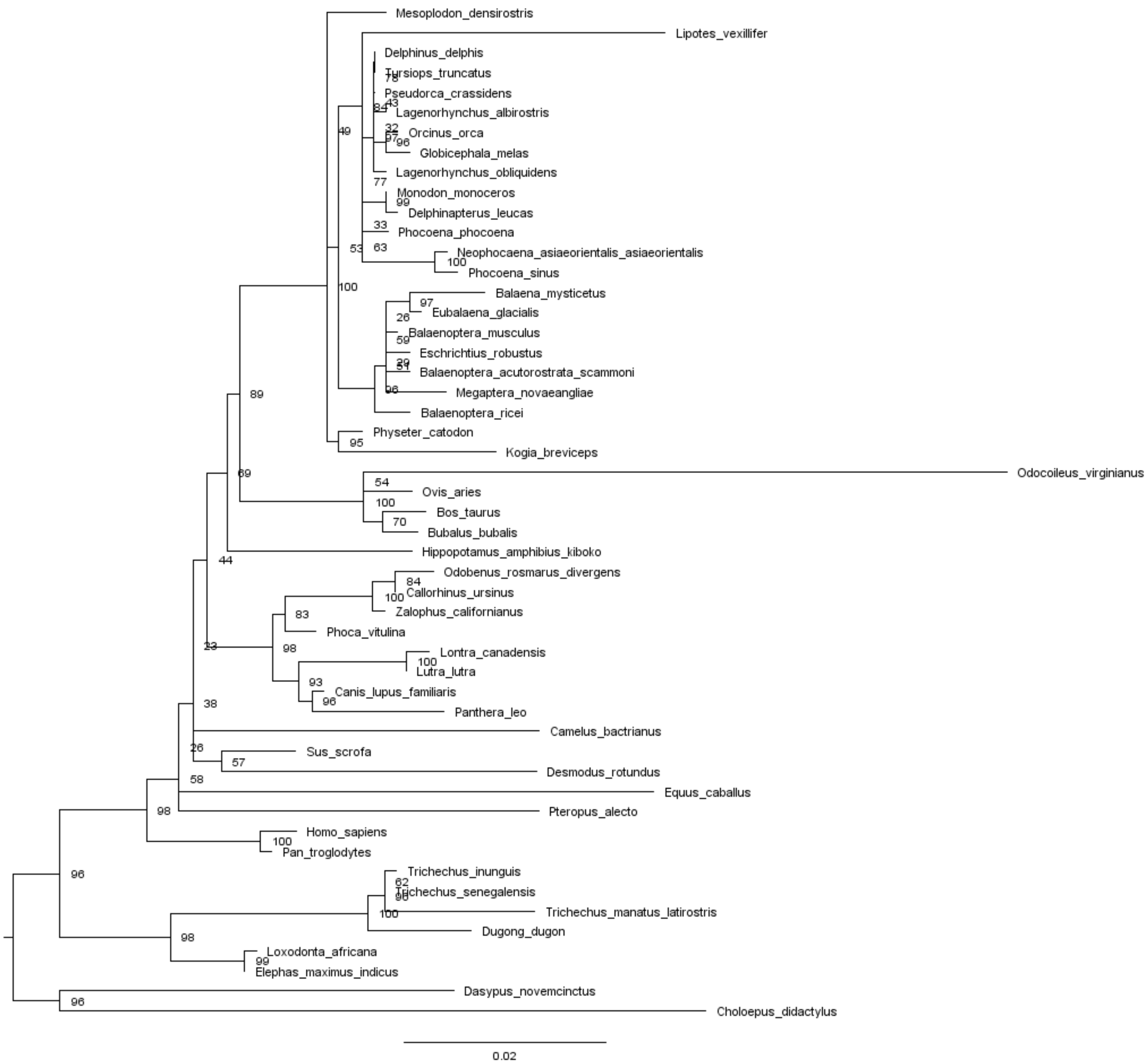

Figure S42. Maximum likelihood phylogenetic tree reconstructed with IQ-TREE 2 using the amino acid alignment of TRPV4 gene. Branch confidence is determined by bootstrap values in the nodes and branch lengths are in scale.

Supplementary tables legends:

Table S1. Table listing the NCBI accession numbers, publication year, and assembly level of the genome assemblies, as well as taxonomic order and family, and common names for the target cetacean and sirenian species and the main aquatic and terrestrial outgroup mammal species used.

Table S2. Table listing the NCBI accession numbers of gene transcripts for each species included in the dataset. An asterisk (*) indicates sequences retrieved via BLASTn; a dash (–) denotes missing sequences.

Table S3. BLAST summary showing species, gene, alignment scores, query coverage, E-value, identity percentage, accession number, query ID, match/mismatch scores, and gap penalties.

Table S4. Likelihood statistics (*lnL*) for nucleotide and protein maximum likelihood trees inferred for each gene, including the Bayesian Information Criterion (BIC), number of parameters, number of sites, and the best-fit sequence evolution model identified by ModelFinder.

Table S5. Scheme likelihood statistics (lnL) for nucleotide bayesian trees inferred for each gene, including the Scheme Bayesian Information Criterion (BIC), number of parameters, number of sites, number of subsets and the best-fit sequence evolution model identified by PartitionFinder.

Table S6. Results of branch model analyses from CodeML for the second set of selection hypotheses to investigate potential phylogenetic bias, grouping Cetacea and Hippopotamidae (Whippomorpha) and Sirenia and Proboscidea (Tethytheria). LRT refers to the likelihood ratio test; *p*-value indicates the adjusted *p*-value. The first value in the "ω values" column always corresponds to the background branches. Subsequent values represent the foreground branches for each model: 1ω, null model (one ratio); 2ω, a single ω for Whippomorpha and Tethytheria (convergence); 3ω, separate ω values for Whippomorpha and Tethytheria; 5ω, crown and stem lineages of Whippomorpha and Tethytheria; and 7ω, separate ω values for stem Whippomorpha, crown Cetacea and Hippopotamidae, stem Tetytheria, crown Sirenia and crown Proboscidae. Bolded *p*-values indicate statistically significant models for the tested lineages.

Table S7. PCOC results per gene, including the number of convergent sites, overall proportion of indels (Indel_prop) and proportion of indels among convergent lineages(Indel_prop ConvLeaves), and posterior probabilities from the PCOC, PC, and OC models. Bolded sites indicate convergent positions also identified by the branch-site model in CodeML, with the same amino acid substitutions.

Table S8. Total folding free energy change (ΔΔG) estimated by FoldX for genes under positive selection, comparing protein stability across representative species of Cetacea, Sirenia, and their terrestrial relatives. Protein stability was classified as either "Stabilizing" or "Destabilizing". Columns such as "Entropy_sidec", "Entropy_mainc", "disulfide", "partial_covalent", and "Energy_Ionisation" contained only zero values across all genes and species and were excluded for clarity.

Table S9. Root-mean-square deviation (RMSD, Å) values from pairwise structural alignments of TRP proteins between terrestrial and aquatic mammals. The number of pruned atom pairs used for superposition is shown for each comparison.
