# Supplementary material for: Sensing Underwater: Diversifying Selection, Convergent Evolution and Inactivation in Sensory Receptors’ Genes of Aquatic Mammals
Source: J Mol Evol. 2026 Jul 1;94(4):629–54. doi: 10.1007/s00239-026-10327-2 (PMC13433407; doi:10.1007/s00239-026-10327-2)
Supplement: Supplementary file 2 [file 239_2026_10327_MOESM2_ESM.docx]

**Sensing underwater: diversifying selection, convergent evolution and inactivation in sensory receptors’ genes of aquatic mammals for life underwater**

**Journal of Molecular Evolution**

Ana Luiza Lein-Borba^1*^, Giovanna Selleghin-Veiga^1^, Beatriz Daros^1^, Letícia Magpali, Mariana F. Nery^1*^

^1^University of Campinas (Department of Genetics, Evolution, Microbiology and Immunology), Campinas, SP, Brazil

^2^Dalhousie University (Department of Biology), Halifax, Nova Scotia, Canada

^*^Corresponding author

E-mail addresses: analuizaclein@gmail.com (ALLB), mariananery@gmail.com (MFN)

**Fig S43** Gene neighborhood of four candidate pseudogenes in *Balaenoptera musculus*, *Orcinus orca* and *Bos taurus*. For each gene (center), four upstream and four downstream genes are shown, enabling comparison of synteny, conservation of genomic context, and lineage-specific losses or rearrangements. Pseudogenes and non-coding RNAs are indicated as annotated in NCBI. (a) PKD1L3; (b) PKD2L1; (c) TRPA1; (d) TRPM5

**Figure S44**. Ribbon diagrams depicting a single subunit of TRPA1 protein structures predicted by AlphaFold3. **a**. *Balaenoptera musculus* TRPA1 protein (light blue). **b**. *Bos taurus* TRPA1 protein (beige). **c**. Graphic of Predicted Alignment Error (PAE) for the *B. musculus* model generated using AlphaFold3. **d**. Graphic of PAE for the *B. taurus* model generated using AlphaFold3. The reference bar on the bottom shows the color coded Expected Position Error.

**Figure S45**. Ribbon diagrams depicting a single subunit of TRPM2 protein structures predicted by AlphaFold3. **a**. *Tursiops truncatus* TRPM2 protein (light blue). **b**. *Bos taurus* TRPM2 protein (beige). **c**. Graphic of Predicted Alignment Error (PAE) for the *T. truncatus* model generated using AlphaFold3. **d**. Graphic of PAE for the *B. taurus* model generated using AlphaFold3. The reference bar on the bottom shows the color coded Expected Position Error.

**Figure S46**. Ribbon diagrams depicting a single subunit of TRPM3 protein structures predicted by AlphaFold3. **a**. *Orcinus orca* TRPM3 protein (light blue). **b**. *Bubalus bubalis* TRPM3 protein (beige). **c**. Graphic of Predicted Alignment Error (PAE) for the *O. orca* model generated using AlphaFold3. **d**. Graphic of PAE for the *B. bubalis* model generated using AlphaFold3. The reference bar on the bottom shows the color coded Expected Position Error.

**Figure S47**. Ribbon diagrams depicting a single subunit of TRPM4 protein structures predicted by AlphaFold3. **a**. *Trichechus manatus* TRPM4 protein (light blue). **b**. *Loxodonta africana* TRPM4 protein (beige). **c**. Graphic of Predicted Alignment Error (PAE) for the *T. manatus* model generated using AlphaFold3. **d**. Graphic of PAE for the *L. africana* model generated using AlphaFold3. The reference bar on the bottom shows the color coded Expected Position Error.

**Figure S48**. Ribbon diagrams depicting a single subunit of TRPM5 protein structures predicted by AlphaFold3. **a**. *Balaenoptera musculus* TRPM5 protein (light blue). **b**. *Bos taurus* TRPM5 protein (beige). **c**. Graphic of Predicted Alignment Error (PAE) for the *B. musculus* model generated using AlphaFold3. **d**. Graphic of PAE for the *B. taurus* model generated using AlphaFold3. The reference bar on the bottom shows the color coded Expected Position Error.

**Figure S49**. Ribbon diagrams depicting a single subunit of TRPM8 protein structures predicted by AlphaFold3. **a**. *Tursiops truncatus* TRPm8 protein (light blue). **b**. *Bos taurus* TRPM8 protein (beige). **c**. Graphic of Predicted Alignment Error (PAE) for the *T. truncatus* model generated using AlphaFold3. **d**. Graphic of PAE for the *B. taurus* model generated using AlphaFold3. The reference bar on the bottom shows the color coded Expected Position Error.

**Figure S50**. Ribbon diagrams depicting a single subunit of TRPV2 protein structures predicted by AlphaFold3. **a**. *Trichechus manatus* TRPV2 protein (light blue). **b**. *Loxodonta africana* TRPV2 protein (beige). **c**. Graphic of Predicted Alignment Error (PAE) for the *T. manatus* model generated using AlphaFold3. **d**. Graphic of PAE for the *L. africana* model generated using AlphaFold3. The reference bar on the bottom shows the color coded Expected Position Error.

**Figure S51**. Ribbon diagrams depicting a single subunit of TRPV3 protein structures predicted by AlphaFold3. **a**. *Trichechus manatus* TRPV3 protein (light blue). **b**. *Elephas maximus* TRPV3 protein (beige). **c**. Graphic of Predicted Alignment Error (PAE) for the *T. manatus* model generated using AlphaFold3. **d**. Graphic of PAE for the *E. maximus* model generated using AlphaFold3. The reference bar on the bottom shows the color coded Expected Position Error.

**Figure S52.** Graphics of Predicted Alignment Error (PAE) for TRPM4 (a, b), TRPM8 (c, d), TRPV2 (e, f) and TRPV3 (g, h) protein models generated using AlphaFold3. **a**. Graphic of PAE for *Balaenoptera musculus* TRPM4 protein. **b.** Graphic of PAE for *Bos taurus* TRPM4 protein. **c**. Graphic of PAE for *B. musculus* TRPM8 protein. **d.** Graphic of PAE for *B. taurus* TRPM8 protein. **e**. Graphic of PAE for *Orcinus orca* TRPV2 protein. **f.** Graphic of PAE for *B. taurus* TRPV2 protein. **g**. Graphic of PAE for *B. musculus* TRPV3 protein. **h.** Graphic of PAE for *B. taurus* TRPV3 protein. **i.** Reference bar showing the color coded Expected Position Error.

**Figure S53.** Structural alignment of the AlphaFold3-predicted TRPA1 protein from *B. musculus* (**a**) and *B. taurus* (**b**) with chain A of the corresponding human Cryo-EM structure. RMSD values displayed below each alignment.

**Figure S54.** Structural alignment of the AlphaFold3-predicted TRPM2 protein from *T. truncatus* (**a**) and *B. taurus* (**b**) with chain A of the corresponding human Cryo-EM structure. RMSD values displayed below each alignment.

**Figure S55.** Structural alignment of the AlphaFold3-predicted TRPM3 protein from *O. orca* (**a**) and *B. bubalis* (**b**) with chain A of the corresponding human Cryo-EM structure. RMSD values displayed below each alignment.

**Figure S56.** Structural alignment of the AlphaFold3-predicted TRPM4 protein from *B. musculus* (**a**), *B. bubalis* (**b**), *T. manatus* (**c**) and *L. africana* (**d**) with chain A of the corresponding human Cryo-EM structure. RMSD values displayed below each alignment.

**Figure S57.** Structural alignment of the AlphaFold3-predicted TRPM8 protein from *B. musculus* (**a**), *T. truncatus* (**b**) and *B. taurus* (**c**) with chain A of the corresponding human Cryo-EM structure. RMSD values displayed below each alignment.

**Figure S58.** Structural alignment of the AlphaFold3-predicted TRPV2 protein from *O. orca* (**a**), *B. taurus* (**b**), *T. manatus* (**c**) and *L. africana* (**d**) with chain A of the corresponding human ankyrin repeat domain resolved by X-ray crystallography. RMSD values displayed below each alignment.

**Figure S59.** Structural alignment of the AlphaFold3-predicted TRPV3 protein from *B. musculus* (**a**), *B. taurus* (**b**), *T. manatus* (**c**) and *E. maximus* (**d**) with chain A of the corresponding human Cryo-EM structure. RMSD values displayed below each alignment.
