# Supplementary material for: Sensing Underwater: Diversifying Selection, Convergent Evolution and Inactivation in Sensory Receptors’ Genes of Aquatic Mammals
Source: J Mol Evol. 2026 Jul 1;94(4):629–54. doi: 10.1007/s00239-026-10327-2 (PMC13433407; doi:10.1007/s00239-026-10327-2)
Supplement: Supplementary file 3 [file 239_2026_10327_MOESM3_ESM.docx]

**Sensing underwater: diversifying selection, convergent evolution and inactivation in sensory receptors’ genes of aquatic mammals for life underwater**

**Journal of Molecular Evolution**

Ana Luiza Lein-Borba^1*^, Giovanna Selleghin-Veiga^1^, Beatriz Daros^1^, Letícia Magpali, Mariana F. Nery^1*^

^1^University of Campinas (Department of Genetics, Evolution, Microbiology and Immunology), Campinas, SP, Brazil

^2^Dalhousie University (Department of Biology), Halifax, Nova Scotia, Canada

^*^Corresponding author

E-mail addresses: analuizaclein@gmail.com (ALLB), mariananery@gmail.com (MFN)

**Table S1.** Table listing the NCBI accession numbers, publication year, and assembly level of the genome assemblies, as well as taxonomic order and family, and common names for the target cetacean and sirenian species and the main aquatic and terrestrial outgroup mammal species used.

| Order | Family | Species | Common name | NCBI RefSeq genome assembly | Year | Level |
| --- | --- | --- | --- | --- | --- | --- |
| Artiodactyla | Ziphiidae  Kogiidae  Phocoenidae  Lipotidae  Delphinidae  Monodontidae  Physeteridae  Eschrichtiidae  Balaenidae  Balaenopteridae    Hippopotamidae  Bovidae  Cervidae  Suidae  Camelidae | *Mesoplodon densirostris*  *Kogia breviceps*  *Phocoena phocoena*  *Neophocaena asiaeorientalis asiaeorientalis*  *Phocoena sinus*  *Lipotes vexillifer*  *Delphinus delphis*  *Lagenorhynchus albirostris*  *Lagenorhynchus obliquidens*  *Globicephala melas*  *Pseudorca crassidens*  *Orcinus orca*  *Tursiops truncatus*  *Delphinapterus leucas*  *Monodon monoceros*  *Physeter catodon*  *Eschrichtius robustus*  *Eubalaena glacialis*  *Balaena mysticetus*  *Megaptera novaeangliae*  *Balaenoptera acutorostrata scammoni*  *Balaenoptera musculus*  *Balaenoptera ricei*  *Hippopotamus amphibius kiboko*  *Bubalus bubalis*  *Bos taurus*  *Ovis aries*  *Odocoileus virginianus*  *Sus scrofa*  *Camelus bactrianus* | Blainville's beaked whale  Pygmy sperm whale  Harbor porpoise  Yangtze finless porpoise    Vaquita  Yangtze river dolphin  Saddleback dolphin  White-beaked dolphin  Pacific white-sided dolphin  Long-finned pilot whale  False killer whale  Killer whale  Common bottlenose dolphin  Beluga whale  Narwhal  Sperm whale  Grey whale  North atlantic right whale  Bowhead whale  Humpback whale  Minke whale  Blue whale  Rice's whale  Hippopotamus  Water buffalo  Cattle  Sheep  White-tailed deer  Pig  Bactrian camel | GCF_025265405.1  GCF_026419965.1  GCF_963924675.1  GCF_003031525.2  GCF_008692025.1  GCF_000442215.2  GCF_949987515.1  GCF_949774975.1  GCF_003676395.1  GCF_963455315.1  GCF_039906515.1  GCF_937001465.1  GCF_011762595.1  GCF_002288925.2  GCF_005190385.1  GCF_002837175.3  GCF_028021215.1  GCF_028564815.1  -  GCA_041834305.1  GCF_949987535.1  GCF_009873245.2  GCF_028023285.1  GCF_030028045.1  GCF_019923935.1  GCF_002263795.3  GCF_016772045.2  GCF_002102435.1  GCF_000003025.6  GCF_000767855.1 | 2022  2022  2024  2018  2019  2013  2023  2023  2018  2023  2024  2022  2020  2019  2019  2023  2023  2023  2015  2024  2023  2020  2023  2023  2021  2023  2023  2017  2017  2014 | chromosome  chromosome  chromosome  scaffold  chromosome  scaffold  chromosome  chromosome  scaffold  chromosome  chromosome  chromosome  chromosome  scaffold  scaffold  chromosome  chromosome  chromosome  chromosome  chromosome  chromosome  chromosome  chromosome  chromosome  complete  chromosome  chromosome  scaffold  chromosome  scaffold |
| Perissodactyla | Equidae | *Equus caballus* | Horse | GCF_002863925.1 | 2018 | chromosome |
| Carnivora | Odobenidae  Otariidae  Phocidae  Mustelidae  Canidae  Felidae | *Odobenus rosmarus divergens*  *Zalophus californianus*  *Callorhinus ursinus*  *Phoca vitulina*  *Lutra lutra*  *Lontra canadensis*  *Canis lupus familiaris*  *Panthera leo* | Pacific walrus  California sea lion  Northern fur seal  Harbor seal  Eurasian river otter  Northern american river otter  Dog  Lion | GCF_000321225.1  GCF_009762305.2  GCF_003265705.1  GCF_004348235.1  GCF_902655055.1  GCF_010015895.1  GCF_011100685.1  GCF_018350215.1 | 2013  2020  2018  2019  2020  2020  2020  2021 | scaffold  chromosome  scaffold  scaffold  chromosome  scaffold  chromosome  chromosome |
| Chiroptera | Phyllostomidae | *Desmodus rotundus* | Common vampire bat | GCF_022682495.1 | 2022 | chromosome |
|  | Pteropodidae | *Pteropus alecto* | Black flying fox | GCF_000325575.1 | 2013 | scaffold |
| Primates | Hominidae | *Pan troglodytes* | Chimpanzee | GCF_028858775.2 | 2024 | complete |
|  |  | *Homo sapiens* | Human | GCF_000001405.40 | 2022 | chromosome |
| Sirenia | Trichechidae  Dugongidae | *Trichechus manatus latirostris*  *Trichechus senegalensis*  *Trichechus inunguis*  *Dugong dugon* | Florida manatee  African manatee  Amazon manatee  Dugong | GCF_000243295.1  -  GCA_046562895.1  GCA_030035585.1 | 2012  2024  2025  2023 | scaffold  chromosome  chromosome  chromosome |
| Proboscidea | Elephantidae | *Elephas maximus indicus* | Asian elephant | GCF_024166365.1 | 2022 | chromosome |
|  |  | *Loxodonta africana* | African savanna elephant | GCF_000001905.1 | 2019 | chromosome |
| Cingulata | Dasypodidae | *Dasypus novemcinctus* | Nine-banded armadillo | GCF_030445035.1 | 2023 | chromosome |
| Pilosa | Megalonychidae | *Choloepus didactylus* | Southern two-toed sloth | GCF_015220235.1 | 2020 | chromosome |

**Table S2**: PDB accession codes of human TRP channel Cryo-EM structures in apo and/or closed conformational states used in this study. An asterisk (*) indicates proteins for which no full-length structure was available; in these cases, only the ankyrin repeat domain resolved by X-ray crystallography was used. No human Cryo-EM structures in apo and/or closed conformational states were available in the PDB for PKD1L3, PKD2L1, TRPM5, TRPV1, and TRPV4 at the time of analysis.

| Gene | PDB accession code | Source |
| --- | --- | --- |
| TRPA1 | pdb_00003j9p | [Paulsen et al. 2015](https://paperpile.com/c/gtwP8o/wKLw+ELOj+fIwT+wU9c+iUJe+fy7Q) |
| TRPM2 | pdb_00006puo | [Huang et al. 2019](https://paperpile.com/c/gtwP8o/wKLw+ELOj+fIwT+wU9c+iUJe+fy7Q) |
| TRPM3 | pdb_00009j84 | To be published |
| TRPM4 | pdb_00009mta | [Teixeira-Duarte et al. 2026](https://paperpile.com/c/gtwP8o/wKLw+ELOj+fIwT+wU9c+iUJe+fy7Q) |
| TRPM8 | pdb_00009p8y | [Choi et al. 2026](https://paperpile.com/c/gtwP8o/wKLw+ELOj+fIwT+wU9c+iUJe+fy7Q) |
| TRPV2* | pdb_00002f37 | [McCleverty et al. 2006](https://paperpile.com/c/gtwP8o/wKLw+ELOj+fIwT+wU9c+iUJe+fy7Q) |
| TRPV3 | pdb_00006mho | [Zubcevic et al. 2018](https://paperpile.com/c/gtwP8o/wKLw+ELOj+fIwT+wU9c+iUJe+fy7Q) |

**Table S3**. Table listing the NCBI accession numbers of gene transcripts for each species included in the dataset. An asterisk (*) indicates sequences retrieved via BLASTn; a dash (–) denotes missing sequences.

| Genes/Transcript IDs | | | | | | | | | | | |  |
| --- | --- | --- | --- | --- | --- | --- | --- | --- | --- | --- | --- | --- |
| Species | PKD1L3 | PKD2L1 | TRPA1 | TRPM2 | TRPM3 | TRPM4 | TRPM5 | TRPM8 | TRPV1 | TRPV2 | TRPV3 | TRPV4 |
| *Mesoplodon densirostris* | XM_060083176.1 | XM_060095713.1 |  | XM_060100384.1 | XM_060100866.1 | XM_060084291.1 | XM_060103884.1 | XM_060106035.1 | XM_060081399.1 | XM_060082889.1 | XM_060081235.1 | XM_060118653.1 |
| *Kogia breviceps* | XM_059043810.1 | XM_059053576.1 |  | XM_059064828.1 | XM_059072697.1 | XM_059046099.1 | XM_059069199.1 | XM_059052901.1 | XM_059046682.1 | XM_059048428.1 | XM_059046705.2 | XM_059039443.2 |
| *Delphinus delphis* | XM_059999300.1 | XM_060033464.1 |  | XM_060009759.1 | XM_060015342.1 | XM_059999523.1 | XM_060019734.1 | XM_060015472.1 | XM_059996881.1 | XM_059998713.1 | XM_059998697.1 | XM_060029402.1 |
| *Phocoena phocoena* | XM_065898393.1 | XM_065893895.1 |  | XM_065877054.1 | XM_065879759.1 | XM_065898270.1 | XM_065882487.1 | XM_065880784.1 | XM_065896454.1 | XM_065896695.1 | XM_065897659.1 | XM_065890577.1 |
| *Neophocaena asiaeorientalis asiaeorientalis* | XM_024735707.1 | XM_037001241.1 |  | XM_024758368.1 | XM_024738984.1 | XM_024768186.1 | XM_024749214.1 | XM_024752768.1 | XM_024736548.1 | XM_024753153.1 | XM_024737284.1 | XM_024741485.1 |
| *Lipotes vexillifer* | XM_007463000.1 | XM_007470453.1 |  | XM_007452492.1 | XM_007446058.1 | XM_007463442.1 | XM_007466009.1 | XM_007463909.1 | XM_007454184.1 | XM_007462658.1 | XM_007454186.1 | XM_007448216.1 |
| *Lagenorhynchus albirostris* | XM_060129812.1 | XM_060125766.1 |  | XM_060151069.1 | XM_060153439.1 | XM_060129680.1 | XM_060158207.1 | XM_060151220.1 | XM_060134868.1 | XM_060135889.1 | XM_060135830.1 | XM_060170545.1 |
| *Lagenorhynchus obliquidens* | XM_027119414.1 | XM_027112317.1 |  | XM_027079926.1 | XM_027105962.1 | XM_027080167.1 | XM_027090865.1 | XM_027117662.1 | XM_027130681.1 | XM_027131121.1 | XM_027130687.1 | XM_027124505.1 |
| *Globicephala melas* | XM_060288303.1 | XM_060286006.1 |  | XM_030835578.2 | * | XM_030873586.2 | XM_030834014.1 | XM_030866449.1 | XM_030861763.1 | XM_033422822.2 | XM_030861772.2 | XM_060310302.1 |
| *Pseudorca crassidens* | XM_067718410.1 | XM_067709703.1 |  | XM_067739645.1 | XM_067744178.1 | XM_067719981.1 | XM_067750731.1 | XM_067740157.1 | XM_067717398.1 | XM_067715173.1 | XM_067716852.1 | XM_067700519.1 |
| *Orcinus orca* | XM_033434308.2 | XM_049697126.1 |  | XM_033418456.2 | XM_049710919.1 | XM_033430495.2 | XM_049713416.1 | XM_049712392.1 | XM_049702097.1 | XM_004321400.3 | XM_012531979.2 | XM_049698391.1 |
| *Tursiops truncatus* | * | XM_033842393.1 |  | XM_033856214.1 | XM_033859311.1 | XM_033846118.1 | XM_033861746.1 | XM_033860370.1 | XM_019926882.2 | XM_032615965.1 | XM_033846867.1 | XM_033837066.1 |
| *Mesoplodon densirostris* | XM_060083176.1 | XM_060095713.1 |  | XM_060100384.1 | XM_060100866.1 | XM_060084291.1 | XM_060103884.1 | XM_060106035.1 | XM_060081399.1 | XM_060082889.1 | XM_060081235.1 | XM_060118653.1 |
| *Kogia breviceps* | XM_059043810.1 | XM_059053576.1 |  | XM_059064828.1 | XM_059072697.1 | XM_059046099.1 | XM_059069199.1 | XM_059052901.1 | XM_059046682.1 | XM_059048428.1 | XM_059046705.2 | XM_059039443.2 |
| *Delphinus delphis* | XM_059999300.1 | XM_060033464.1 |  | XM_060009759.1 | XM_060015342.1 | XM_059999523.1 | XM_060019734.1 | XM_060015472.1 | XM_059996881.1 | XM_059998713.1 | XM_059998697.1 | XM_060029402.1 |
| *Phocoena phocoena* | XM_065898393.1 | XM_065893895.1 |  | XM_065877054.1 | XM_065879759.1 | XM_065898270.1 | XM_065882487.1 | XM_065880784.1 | XM_065896454.1 | XM_065896695.1 | XM_065897659.1 | XM_065890577.1 |
| *Neophocaena asiaeorientalis asiaeorientalis* | XM_024735707.1 | XM_037001241.1 |  | XM_024758368.1 | XM_024738984.1 | XM_024768186.1 | XM_024749214.1 | XM_024752768.1 | XM_024736548.1 | XM_024753153.1 | XM_024737284.1 | XM_024741485.1 |
| *Lipotes vexillifer* | XM_007463000.1 | XM_007470453.1 |  | XM_007452492.1 | XM_007446058.1 | XM_007463442.1 | XM_007466009.1 | XM_007463909.1 | XM_007454184.1 | XM_007462658.1 | XM_007454186.1 | XM_007448216.1 |
| *Phocoena sinus* | XM_032614824.1 | XM_032609178.1 |  | XM_032629736.1 | XM_032635138.1 | XM_032612165.1 | XM_032641234.1 | XM_032637415.1 | XM_032616759.1 | XM_029211938.1 | XM_032617589.1 | XM_032602894.1 |
| *Monodon monoceros* | XM_029205635.1 | XM_029244813.1 |  | XM_029214814.1 | XM_024126887.2 | XM_029205661.1 | XM_029209427.1 | XM_029225301.1 | XM_029211930.1 | XM_022560868.2 | XM_029212768.1 | XM_029228322.1 |
| *Delphinapterus leucas* | XM_022583300.2 | XM_022569054.1 |  | XM_030760855.1 | XM_032891718.1 | XM_022585110.1 | XM_030764355.1 | XM_022568021.1 | XM_022595823.2 | XM_055090232.1 | XM_030763282.1 | XM_022600339.1 |
| *Physeter catodon* | XM_055078918.1 | XM_055081434.1 |  | XM_028492728.2 | XM_024126887.2 | * | XM_024133463.1 | XM_024124651.2 | XM_055090133.1 | XM_051824442.1 | XM_055090936.1 | XM_028480246.1 |
| *Megaptera novaeangliae* | * | * | * | * | * | * | * | * | * | * | * | * |
| *Eschrichtius robustus* | XM_068527093.1 | XM_068547904.1 | XM_068525479.1 | XM_068545804.1 | XM_068553750.1 | XM_068528323.1 | * | XM_068543723.1 | XM_068530615.1 | XM_068530220.1 | XM_068530361.1 | XM_068562728.1 |
| *Eubalaena glacialis* | XM_061172421.1 | XM_061194291.1 | XM_061171718.1 | XM_061192824.1 | XM_061200350.1 | XM_061173130.1 | XM_061202276.1 | XM_061206073.1 | XM_061175510.1 | XM_061176284.1 | XM_061175086.1 | XM_061170476.1 |
| *Balaenoptera acutorostrata scammoni* | XM_057534484.1 | XM_057530591.1 | XM_007185124.2 | XM_057544828.1 | XM_007182257.1 | XM_007168276.2 | XM_057552458.1 | XM_007184754.1 | XM_007177448.2 | XM_057536619.1 | XM_057536091.1 | XM_057526750.1 |
| *Balaenoptera musculus* | XM_036832336.1 | XM_036829299.1 | XM_036830084.1 | XM_036851801.1 | XM_036855282.1 | XM_036834281.1 | XM_036861047.1 | XM_036858039.1 | XM_036838429.1 | XM_036836849.1 | XM_036835602.1 | XM_036874149.1 |
| *Balaenoptera ricei* | XM_059903766.1 | XM_059900872.1 | XM_059901727.1 | XM_059921696.1 | XM_059924893.1 | XM_059903698.1 | XM_059929977.1 | XM_059927505.1 | XM_059907394.1 | XM_059906648.1 | XM_059906642.1 | XM_059895730.1 |
| *Balaena mysticetus* | - | - | - | - | - | - | - | - | - | - | - | - |
| *Hippopotamus amphibius kiboko* | XM_057712899.1 | XM_057736946.1 | XM_057736316.1 | XM_057697832.1 | XM_057720878.1 | XM_057712838.1 | XM_057727186.1 | XM_057745186.1 | XM_057715640.1 | XM_057716490.1 | XM_057715104.1 | XM_057746505.1 |
| *Bos taurus* | XM_059877311.1 | XM_015460714.3 | XM_015474483.2 | XM_024996726.1 | NM_001076236.1 | XM_059877596.1 | XM_024987522.1 | XM_005204960.5 | XM_015458647.2 | NM_001024493.1 | XM_015458625.3 | XM_005217903.5 |
| *Odocoileus virginianus* | XM_020882562.1 | XM_020870617.1 | XM_020907330.1 | XM_020883579.1 | XM_020902000.1 | XM_020885673.1 | XM_020883241.1 | XM_020893850.1 | XM_020881189.1 | XM_020892230.1 | XM_020913266.1 | XM_020906945.1 |
| *Ovis aries* | XM_060397841.1 | XM_015103379.4 | XM_027973040.2 | XM_027961066.3 | XM_042243350.1 | XM_004015802.5 | XM_042237931.2 | XM_015091832.4 | XM_027975474.3 | XM_015098668.3 | XM_042255524.1 | XM_042234702.2 |
| *Bubalus bubalis* | XM_045163453.1 | XM_044935330.2 | XM_006047233.4 | XM_044947600.2 | XM_045165065.1 | XM_025269485.3 | XM_025286915.3 | XM_006053728.4 | XM_006079707.3 | XM_006079460.4 | XM_006079709.3 | XM_044930050.1 |
| *Sus scrofa* | - | XM_001927121.4 | XM_021089237.1 | XM_021071509.1 | XM_021074417.1 | XM_021094710.1 | XM_021082617.1 | XM_021074741.1 | XM_013981216.2 | XM_013980349.2 | XM_013981215.2 | XM_021071776.1 |
| *Camelus bactrianus* | XM_045521134.1 | XM_045507384.1 | XM_010967140.2 | XM_045503965.1 | XM_045515112.1 | XM_045510682.1 | XM_045517778.1 | XM_010948162.2 | XM_010950234.2 | XM_010974112.2 | XM_010950229.2 | XM_045523105.1 |
| *Equus caballus* | XM_023637242.1 | XM_023625349.1 | XM_001493464.3 | XM_023630285.1 | XM_005604961.3 | XM_023650090.1 | XM_023654710.1 | XM_001499486.6 | XM_014727972.2 | XM_023653560.1 | XM_023653271.1 | XM_023648063.1 |
| *Odobenus rosmarus divergens* | XM_012565704.1 | XM_004401914.1 | XM_004408712.1 | XM_012565956.1 | XM_012561398.1 | XM_004415075.1 | XM_012563540.1 | XM_004396072.2 | XM_025851492.1 | XM_004415013.1 | XM_004412123.1 | XM_004416179.2 |
| *Zalophus californianus* | XM_035725260.1 | XM_027594253.1 | XM_027623985.2 | XM_027585316.2 | XM_027615317.2 | XM_027619002.2 | XM_027579642.2 | XM_027590139.2 | XM_027567722.2 | XM_027625504.2 | XM_035724549.1 | XM_027577422.2 |
| *Phoca vitulina* | XM_032424042.1 | XM_032432575.1 | XM_032422025.1 | XM_032392558.1 | XM_032427586.1 | XM_032388413.1 | XM_032414836.1 | XM_032395253.1 | XM_032399102.1 | XM_032432032.1 | XM_032399089.1 | XM_032398004.1 |
| *Callorhinus ursinus* | XM_025888859.1 | XM_025880852.1 | XM_025870526.1 | XM_025863567.1 | - | XM_025849877.1 | XM_025878614.1 | XM_025886642.1 | XM_028095455.1 | XM_025863945.1 | XM_025851399.1 | XM_025889376.1 |
| *Lutra lutra* | XM_047711834.1 | XM_047703553.1 | XM_047728437.1 | XM_047718589.1 | XM_047699836.1 | XM_047709967.1 | XM_047747394.1 | XM_047722946.1 | XM_004412121.2 | XM_047707362.1 | XM_047706694.1 | XM_047698134.1 |
| *Lontra canadensis* | XM_032853712.1 | XM_032882353.1 | XM_032869254.1 | XM_032865207.1 | - | XM_032839288.1 | XM_032879921.1 | XM_032857975.1 | XM_032848995.1 | XM_032841213.1 | XM_032848971.1 | XM_032847403.1 |
| *Canis lupus familiaris* | XM_038667171.1 | XM_038440280.1 | XM_038441123.1 | XM_038443642.1 | XM_038655209.1 | XM_038656349.1 | XM_038424702.1 | NM_001110769.2 | XM_038674983.1 | XM_038665706.1 | XM_038677763.1 | NM_001127315.1 |
| *Panthera leo* | XM_042917916.1 | XM_042908212.1 | XM_042924314.1 | XM_042903262.1 | - | XM_042920337.1 | XM_042905944.1 | XM_042950598.1 | XM_042915845.1 | XM_042915201.1 | XM_042916783.1 | XM_042910253.1 |
| *Desmodus rotundus* | XM_053914869.1 | XM_053923570.1 | XM_024572316.3 | XM_053917638.1 | XM_053923777.1 | XM_053915213.1 | XM_053924586.1 | XM_024563974.3 | XM_045199317.2 | XM_024564975.3 | XM_053911158.1 | XM_024566782.3 |
| *Pteropus alecto* | XM_015587729.2 | XM_006925883.2 | XM_015598608.1 | XM_015597627.1 | XM_006918420.2 | XM_015592166.2 | XM_025046280.1 | XM_006909887.2 | XM_006922196.1 | XM_015586664.2 | XM_006922194.1 | XM_015597310.2 |
| *Pan troglodytes* | XM_511095.6 | XM_063782069.1 | XM_519806.7 | XM_024352442.3 | XM_054658878.2 | XM_054673955.2 | XM_054661885.1 | XM_054679580.2 | XM_016931216.4 | XM_063798942.1 | XM_016931205.4 | XM_016924153.4 |
| *Homo sapiens* | XM_024450254.2 | NM_016112.3 | XM_011517625.3 | XM_011529736.3 | NM_001366143.2 | NM_017636.4 | XM_047426858.1 | NM_024080.5 | NM_080704.4 | XM_006721541.5 | NM_001321336.2 | XM_011538631.3 |
| *Trichechus manatus latirostris* | XM_023741926.1 | XM_004370140.2 | XM_012554434.2 | XM_023739067.1 | XM_004375847.1 | XM_004381824.1 | XM_004389435.1 | XM_004390029.1 | XM_004376247.2 | XM_023731062.1 | XM_004376248.2 | XM_012556273.1 |
| *Trichechus senegalensis* | * | * | * | * | * | * | * | * | * | * | * | * |
| *Trichechus inunguis* | * | * | * | * | * | * | * | * | * | * | * | * |
| *Dugong dugon* | * | * | * | * | * | * | * | * | * | * | * | * |
| *Elephas maximus indicus* | XM_049864772.1 | XM_049855035.1 | XM_049853928.1 | XM_049875007.1 | XM_049895512.1 | XM_049901747.1 | XM_049892845.1 | XM_049889034.1 | XM_049859982.1 | XM_049860452.1 | XM_049859879.1 | XM_049865816.1 |
| *Loxodonta africana* | XM_023556506.1 | XM_010589066.2 | XM_003408220.2 | XM_023559172.1 | XM_010587530.2 | XM_023544062.1 | XM_023541586.1 | XM_023557703.1 | XM_023556293.1 | XM_023539610.1 | XM_064271571.1 | XM_064272339.1 |
| *Dasypus novemcinctus* | XM_058279523.1 | XM_023588974.2 | XM_004449641.3 | XM_058296336.1 | XM_012523810.2 | XM_058280560.1 | XM_023588503.1 | XM_023592589.1 | XM_004476268.2 | XM_058283419.1 | XM_058283761.1 | XM_012519636.3 |
| *Choloepus didactylus* | - | XM_006157380.1 | XM_037803420.1 | XM_037830578.1 | XM_037798450.1 | XM_037818990.1 | - | XM_037849528.1 | XM_037810627.1 | XM_037809375.1 | XM_037809975.1 | XM_037817213.1 |

**Table S4**. BLAST summary showing species, gene, alignment scores, query coverage, E-value, identity percentage, accession number, query ID, match/mismatch scores, and gap penalties.

| Species | Gene | Max Score | Total Score | Query Cover | E value | % identity | Accession | Query | Match/  Mismatch Scores | Gap Costs |
| --- | --- | --- | --- | --- | --- | --- | --- | --- | --- | --- |
| *Globicephala melas* | TRPM3 | 2682 | 9793 | 100% | 0.0 | 99.93% | NC_083319.1 | *P. crassidens* | 1, -2 | Linear |
| *Tursiops truncatus* | PKD1L3 | 545 | 6601 | 99% | 4,00E-152 | 100.00% | NC_047052.1 | *D. delphis* | 1, -2 | Linear |
| *Physeter catodon* | TRPM4 | 662 | 1887 | 34% | 0.0 | 94.28% | NW_021145299.1 | *T. truncatus* | 1, -2 | Linear |
|  |  | 566 | 3798 | 64% | 3,00E-158 | 97.30% | NC_041230.1 | *T. truncatus* | 1, -2 | Linear |
| *Megaptera novaeangliae* | PKD1L3 | 551 | 7447 | 99% | 1,00E-153 | 98.71% | CM085713.1 | *B. musculus* | 1, -2 | Linear |
|  | PKD2L1 | 453 | 4255 | 99% | 2,00E-124 | 98.44% | CM085710.1 | *B. musculus* | 1, -2 | Linear |
|  | TRPA1 | 968 | 7046 | 100% | 0.0 | 98.04% | CM085711.1 | *B. musculus* | 1, -2 | Linear |
|  | TRPM2 | 603 | 8185 | 99% | 3,00E-169 | 98.26% | CM085698.1 | *B. musculus* | 1, -2 | Linear |
|  | TRPM3 | 2632 | 9462 | 100% | 0.0 | 99.31% | CM085701.1 | *B. musculus* | 1, -2 | Linear |
|  | TRPM4 | 761 | 6516 | 99% | 0.0 | 98.17% | CM085713.1 | *B. musculus* | 1, -2 | Linear |
|  | TRPM5 | 850 | 5540 | 96% | 0.0 | 95.81% | CM085702.1 | *B. musculus* | 1, -2 | Linear |
|  | TRPM8 | 521 | 6052 | 99% | 6,00E-145 | 98.64% | CM085700.1 | *B. musculus* | 1, -2 | Linear |
|  | TRPV1 | 649 | 4479 | 100% | 0.0 | 98.37% | CM085714.1 | *B. musculus* | 1, -2 | Linear |
|  | TRPV2 | 579 | 4179 | 100% | 3,00E-162 | 98.48% | CM085714.1 | *B. musculus* | 1, -2 | Linear |
| *Eschrichtius robustus* | TRPM5 | 870 | 5556 | 96% | 0.0 | 96.75% | NC_090834.1 | *B. musculus* | 1, -2 | Linear |
| *Trichechus inunguis* | PKD1L3 | 444 | 6333 | 80% | 3,00E-121 | 91.85% | CM102163.1 | *E. maximus* | 1, -2 | Linear |
|  | PKD2L1 | 1491 | 1747 | 58% | 0.0 | 90.23% | CM102149.1 | *E. maximus* | 1, -2 | Linear |
|  |  | 403 | 3424 | 97% | 2,00E-109 | 94.94% | CM102160.1 |  |  |  |
|  | TRPA1 | 331 | 3634 | 70% | 2,00E-87 | 96.06% | CM102158.1 | *E. maximus* | 1, -2 | Linear |
|  | TRPM2 | 444 | 4934 | 70% | 2,00E-121 | 84.89% | CM102146.1 | *L. africana* | 1, -2 | 5, 2 |
|  | TRPM3 | 2416 | 8608 | 100% | 0.0 | 96.45% | CM102155.1 | *E. maximus* | 1, -2 | Linear |
|  | TRPM4 | 392 | 3969 | 86% | 7,00E-106 | 83.26% | CM102157.1 | *L. africana* | 1, -2 | Linear |
|  | TRPM5 | 656 | 6549 | 100% | 0.0 | 99.45% | CM102152.1 | *T. manatus* | 1, -2 | Linear |
|  | TRPM8 | 468 | 4635 | 89% | 1,00E-128 | 94.98% | CM102151.1 | *L. africana* | 1, -2 | Linear |
| *Dugong dugon* | PKD1L3 | 449 | 5878 | 74% | 6,00E-123 | 92.14% | CM057478.1 | *E. maximus* | 1, -2 | Linear |
|  | PKD2L1 | 1408 | 1408 | 49% | 0.0 | 89.21% | CM057465.1 | *E. maximus* | 1, -2 | Linear |
|  |  | 392 | 3395 | 97% | 5,00E-106 | 94.16% | CM057475.1 |  |  |  |
|  | TRPA1 | 331 | 3840 | 74% | 2,00E-87 | 96.06% | CM057474.1 | *E. maximus* | 1, -2 | Linear |
|  | TRPM2 | 455 | 5434 | 79% | 1,00E-124 | 85.11% | CM057460.1 | *L. africana* | 1, -2 | 5, 2 |
|  | TRPM3 | 2405 | 8597 | 99% | 0.0 | 96.31% | CM057471.1 | *E. maximus* | 1, -2 | Linear |
|  | TRPM4 | 392 | 3395 | 76% | 7,00E-106 | 82.96% | CM057466.1 | *L. africana* | 1, -2 | Linear |
|  | TRPM5 | 616 | 6159 | 100% | 4,00E-173 | 97.25% | CM057467.1 | *T. manatus* | 1, -2 | Linear |

**Table S5**. Likelihood statistics (*lnL*) for nucleotide and protein maximum likelihood trees inferred for each gene, including the Bayesian Information Criterion (BIC), number of parameters, number of sites, and the best-fit sequence evolution model identified by ModelFinder.

| Nucleotide maximum likelihood trees | | | | | |
| --- | --- | --- | --- | --- | --- |
| Gene | *lnL* | BIC | Number of parameters | Number of sites | Best Model |
| PKD1L3 | -44213.7175 | 88860.5808 | 49 | 6903 | TIM3e+G4 |
| PKD2L1 | -15237.8360 | 30941.3045 | 59 | 2676 | K3P+R3 |
| TRPA1 | -26678.1919 | 53921.5755 | 68 | 4071 | TIM3e+R4 |
| TRPM2 | -36454.0973 | 73728.5047 | 97 | 4707 | TIM3+F+R3 |
| TRPM3 | -23613.0864 | 48036.4017 | 95 | 5058 | TIM3+F+R3 |
| TRPM4 | -27057.4505 | 54922.1739 | 98 | 3780 | TPM2u+F+I+G4 |
| TRPM5 | -24460.0333 | 49395.1071 | 58 | 3606 | TPM3u+F+R3 |
| TRPM8 | -23230.8840 | 47371.1591 | 110 | 3894 | SYM+R4 |
| TRPV1 | -15583.2213 | 31965.9134 | 102 | 2703 | TN+F+R3 |
| TRPV2 | -20665.125939 | 42130.3635 | 101 | 2535 | TPM3u+F+G4 |
| TRPV3 | -17829.935461 | 36495.9068 | 104 | 3099 | TIM3e+R3 |
| TRPV4 | -16148.9322 | 33140.5021 | 107 | 2631 | TIM3+F+R3 |
| Protein maximum likelihood trees | | | | | |
| PKD1L3 | -28253.0174 | 57009.2062 | 65 | 2301 | JTT+F+G4 |
| PKD2L1 | -8087.4145 | 16562.0566 | 57 | 892 | HIVb+R3 |
| TRPA1 | -13969.5426 | 28407.9322 | 65 | 1357 | JTT+R4 |
| TRPM2 | -19216.3581 | 39087.5955 | 89 | 1569 | JTT+I+G4 |
| TRPM3 | -7860.4464 | 16367.3128 | 87 | 1686 | JTT+R2 |
| TRPM4 | -14103.6738 | 29006.9006 | 112 | 1260 | JTT+F+I+G4 |
| TRPM5 | -13466.4978 | 27422.3258 | 69 | 1202 | JTT+F+G4 |
| TRPM8 | -9000.1904 | 18738.7445 | 103 | 1298 | JTT+R3 |
| TRPV1 | -7046.9290 | 14734.0949 | 95 | 845 | JTT+I+G4 |
| TRPV2 | -10774.733031 | 22210.9944 | 97 | 919 | JTT+I+G4 |
| TRPV3 | -8489.385661 | 17658.2043 | 98 | 1033 | JTT+G4 |
| TRPV4 | -5583.0513 | 11836.9767 | 99 | 877 | JTT+R3 |

**Table S6**. Scheme likelihood statistics (lnL) for nucleotide bayesian trees inferred for each gene, including the Scheme Bayesian Information Criterion (BIC), number of parameters, number of sites, number of subsets and the best-fit sequence evolution model identified by PartitionFinder.

| Gene | Scheme *lnL* | Scheme BIC | Number of parameters | Number of sites | Number of subsets | Sites | Best Model |
| --- | --- | --- | --- | --- | --- | --- | --- |
| PKD1L3 | -44194.3671875 | 88866.0787894 | 54 | 6903 | 1 | - | GTR+G |
| PKD2L1 | -14886.8466796875 | 30744.4190052 | 123 | 2676 | 2 | 1784  892 | GTR+G  TVM+G |
| TRPA1 | -26262.6943359375 | 53630.837317 | 133 | 4071 | 2 | 2714  1357 | TVM+I+G  SYM+G |
| TRPM2 | -39594.689453125 | 80892.0089775 | 198 | 6903 | 2 | 3618  1809 | SYM+I+G  GTR+G |
| TRPM3 | -25581.0380859375 | 52773.2315427 | 188 | 5271 | 2 | 3514  1757 | GTR+I+G  TVM+G |
| TRPM4 | -28969.3779296875 | 59627.2432202 | 201 | 4449 | 2 | 2966  1483 | GTR+I+G  GTR+G |
| TRPM5 | -40587.08203125 | 82445.9253305 | 143 | 7284 | 2 | 4856  2428 | GTR+G  TVM+G |
| TRPM8 | -22347.4296875 | 46447.5041184 | 212 | 3894 | 2 | 2596  1298 | TVM+I+G  HKY+G |
| TRPV1 | -16110.9208984375 | 33786.461071 | 198 | 2703 | 2 | 1802  901 | K81+I+G  TVM+G |
| TRPV2 | -20190.423828125 | 42004.8368305 | 205 | 2757 | 2 | 1838  919 | SYM+I+G  TVM+G |
| TRPV3 | -17233.3740234375 | 36162.9421808 | 211 | 3099 | 2 | 2066  1033 | TVM+I+G  TVM+G |
| TRPV4 | -16442.7626953125 | 34582.6664834 | 214 | 2781 | 2 | 1854  927 | SYM+I+G  GTR+G |

**Table S7**. Results of branch model analyses from CodeML for the second set of selection hypotheses, grouping Cetacea and Hippopotamidae (Whippomorpha) and Sirenia and Proboscidea (Tethytheria). LRT refers to the likelihood ratio test; *p*-value indicates the adjusted *p*-value. The first value in the “ω values” column always corresponds to the background branches. Subsequent values represent the foreground branches for each model: 1ω, null model (one ratio); 2ω, a single ω for Whippomorpha and Tethytheria (convergence); 3ω, separate ω values for Whippomorpha and Tethytheria; 5ω, crown and stem lineages of Whippomorpha and Tethytheria; and 7ω, separate ω values for stem Whippomorpha, crown Cetacea and Hippopotamidae, stem Tetytheria, crown Sirenia and crown Proboscidae. Bolded *p*-values indicate statistically significant models for the tested lineages.

| Gene | Model | *lnL* | LRT | *p* values | ω values |
| --- | --- | --- | --- | --- | --- |
| PKD1L3 | 1ω  2ω  3ω  4ω | -42719.62  -42719.27  -42713.01  -42710.21 | 0.71  12.51  5.60 | 0.40  **0.00**  **0.02** | 0.586  0.581 0.620  0.583 0.388 0.720  0.583 0.392 0.894 0.586 |
| PKD2L1 | 1ω  2ω  3ω  4ω | -14697.55  -14696.48  -14690.03  -14689.99 | 2.14  12.90  0.09 | **0.14**  **0.00**  0.77 | 0.257  0.263 0.215  0.264 0.090 0.293  0.264 0.090 0.306 0.277 |
| TRPA1 | 1ω  2ω  3ω  5ω  7ω | -22185.64  -22185.63  -22185.54  -22183.96  -22183.61 | 0.03  0.18  3.35  4.04 | 0.86  0.67  0.34  0.54 | 0.292  0.256 0.260  0.256 0.271 0.253  0.256 0.455 0.261 0.316 0.226  0.256 0.454 0.284 0.229 0.316 0.231 0.221 |
| TRPM2 | 1ω  2ω  3ω  5ω  7ω | -35359.08  -35359.08  -35358.84  -35348.63  -35342.09 | 0.00  0.47  20.42  13.08 | 0.97  0.49  **0.00**  **0.00** | 0.176  0.176 0.175  0.176 0.171 0.186  0.178 0.045 0.175 0.096 0.249  0.178 0.045 0.158 0.253 0.100 0.170 0.344 |
| TRPM3 | 1ω  2ω  3ω  5ω  7ω | -22665.11  -22645.98  -22643.22  -22643.06  -22639.97 | 38.25  5.52  0.32  6.18 | **0.00**  **0.02**  0.85  **0.05** | 0.054  0.041 0.077  0.041 0.085 0.055  0.041 0.073 0.085 0.048 0.059  0.041 0.077 0.087 0.061 0.048 0.081 0.029 |
| TRPM4 | 1ω  2ω  3ω  5ω  7ω | -26455.59  -26409.18  -26406.53  -26399.6 0  -26388.58 | 92.82  5.30  13.87  22.04 | **0.00**  **0.02**  **0.00**  **0.00** | 0.243  0.192 0.372  0.192 0.416 0.323  0.193 0.349 0.417 0.190 0.388  0.193 0.371 0.480 0.217 0.191 0.294 0.514 |
| TRPM5 | 1ω  2ω  3ω  5ω  7ω | -23527.67  -23509.55  -23496.84  -23494.76  -23486.7 0 | 36.23  25.42  4.16  16.11 | **0.00**  **0.00**  0.12  **0.00** | 0.182  0.161 0.256  0.165 0.331 0.149  0.162 0.269 0.469 0.136 0.269  0.161 0.484 0.406 0.166 2.309 0.216 0.120 |
| TRPM8 | 1ω  2ω  3ω  5ω  7ω | -22202.47  -22174.24  -22159.82  -22158.35  -22144.27 | 56.47  28.84  2.94  28.16 | **0.00**  **0.00**  0.23  **0.00** | 0.099  0.081 0.156  0.081 0.200 0.089  0.081 0.115 0.202 0.063 0.100  0.081 0.118 0.236 0.048 0.063 0.090 0.112 |
| TRPV1 | 1ω  2ω  3ω  5ω  7ω | -15280.75  -15278.54  -15277.81  -15274.56  -15272.73 | 4.42  1.47  6.51  3.66 | **0.04**  0.23  **0.04**  0.16 | 0.146  0.138 0.170  0.138 0.158 0.194  0.138 0.000 0.162 0.244 0.167  0.138 0.000 0.150 0.223 0.238 0.116 0.203 |
| TRPV2 | 1ω  2ω  3ω  5ω  7ω | -20886.26  -20883.81  -20883.44  -20880.31  -20878.34 | 4.89  0.73  6.26  3.95 | 1.00  0.39  **0.04**  0.14 | 0.226  0.213 0.252  0.213 0.244 0.275  0.214 0.646 0.243 0.154 0.322  0.214 0.850 0.255 0.172 0.152 0.379 0.280 |
| TRPV3 | 1ω  2ω  3ω  5ω  7ω | -17349.85  -17224.31  -17204.40  -17194.39  -17184.98 | 251.08  39.82  20.03  18.81 | **0.00**  **0.00**  **0.00**  **0.00** | 0.161  0.091 0.367  0.091 0.480 0.187  0.091 0.413 0.481 0.036 0.246  0.092 0.494 0.544 0.195 0.039 0.293 0.154 |
| TRPV4 | 1ω  2ω  3ω  5ω  7ω | -15304.86  -15297.82  -15293.82  -15292.14  -15286.59 | 14.08  8.00  3.36  11.10 | **0.00**  **0.00**  0.19  **0.00** | 0.057  0.050 0.076  0.050 0.091 0.052  0.050 0.000 0.092 0.038 0.055  0.050 0.000 0.099 0.059 0.040 0.089 0.024 |

**Table S8**. Results of branch model analyses from CodeML for the second set of selection hypotheses to investigate potential phylogenetic bias, grouping Cetacea and Hippopotamidae (Whippomorpha) and Sirenia and Proboscidea (Tethytheria) and Terrestrial Tethytheria and Artiodactyla (convergence) .

| Gene/Foreground branch | LRT | *p* values | Positively selected sites  (BEB > 0.95) |
| --- | --- | --- | --- |
| Tethytheria |  |  |  |
| PKD1L3 | 153.90 | 0.000 | 1319 |
| TRPA1 | 11.23 | 0.001 | - |
| TRPV2 | 9.49 | 0.002 | - |
| Terrestrial Tethytheria and Artiodactyla  (convergence) |  |  |  |
| TRPA1 | 8.14 | 0.004 | - |

**Table S9**. PCOC results per gene, including the number of convergent sites, overall proportion of indels (Indel_prop) and proportion of indels among convergent lineages(Indel_prop ConvLeaves), and posterior probabilities from the PCOC, PC, and OC models. Bolded sites indicate convergent positions also identified by the branch-site model in CodeML, with the same amino acid substitutions.

| Gene | Convergent sites | Indel_prop | Indel_prop  (ConvLeaves) | PCOC_V1 | PC_V1 | OC_V1 |
| --- | --- | --- | --- | --- | --- | --- |
| TRPM3 | 1457 | 0.068 | 0.038 | 0.841 | 0.500 | 0.532 |
| TRPM4 | 320 | 0.000 | 0.000 | 0.835 | 0.500 | 0.508 |
|  | 325 | 0.000 | 0.000 | 0.842 | 0.500 | 0.533 |
|  | 339 | 0.000 | 0.000 | 0.861 | 0.500 | 0.561 |
|  | 660 | 0.000 | 0.000 | 0.830 | 0.500 | 0.559 |
|  | 1048 | 0.021 | 0.000 | 0.909 | 0.500 | 0.420 |
|  | 1147 | 0.085 | 0.000 | 0.940 | 0.500 | 0.495 |
| TRPM5 | 124 | 0.000 | 0.000 | 0.931 | 0.500 | 0.529 |
|  | 592 | 0.000 | 0.000 | 0.815 | 0.500 | 0.528 |
| TRPM8 | 1031 | 0.000 | 0.000 | 0.848 | 0.500 | 0.483 |
|  | 1223 | 0.000 | 0.000 | 0.910 | 0.500 | 0.519 |
| TRPV1 | 334 | 0.000 | 0.000 | 0.837 | 0.500 | 0.465 |
|  | 481 | 0.021 | 0.000 | 0.835 | 0.500 | 0.466 |
| TRPV2 | 718 | 0.061 | 0.115 | 0.883 | 0.500 | 0.547 |
| TRPV3 | **175** | 0.020 | 0.000 | 0.872 | 0.500 | 0.504 |
|  | **393** | 0.000 | 0.000 | 0.963 | 0.500 | 0.554 |
|  | **436** | 0.000 | 0.000 | 0.843 | 0.500 | 0.544 |
|  | **438** | 0.000 | 0.000 | 0.863 | 0.500 | 0.509 |
|  | 534 | 0.000 | 0.000 | 0.844 | 0.500 | 0.544 |

**Table S10.** Root-mean-square deviation (RMSD, Å) values from pairwise structural alignments of TRP proteins between terrestrial and aquatic mammals. The number of pruned atom pairs used for superposition is shown for each comparison.

| Gene/Protein alignment | Pruned atom pairs | RMSD (Å) |
| --- | --- | --- |
| TRPA1 *Bos taurus* with *Balaenoptera musculus* | 845 | 0.973 |
| *Homo sapiens with B. taurus* | 359 | 1.143 |
| *H. sapiens with B. musculus* | 381 | 1.146 |
| TRPM2 *B. taurus* with *Tursiops truncatus* | 1312 | 0.766 |
| *H. sapiens with B. taurus* | 758 | 1.217 |
| *H. sapiens with T. truncatus* | 877 | 1.289 |
| TRPM3 *Bubalus bubalis* with *Orcinus orca* | 1108 | 0.869 |
| *H. sapiens with B. bubalis* | 515 | 1.191 |
| *H. sapiens with B. orca* | 498 | 1.211 |
| TRPM4 *B. bubalis* with *B. musculus* | 931 | 0.944 |
| *Loxodonta africana* with *T. manatus* | 443 | 0.779 |
| *H. sapiens with B. bubalis* | 835 | 1.013 |
| *H. sapiens with B. musculus* | 911 | 1.076 |
| *H. sapiens with L. africana* | 409 | 0.942 |
| *H. sapiens with T. manatus* | 735 | 0.916 |
| TRPM5 *B. taurus* with *B. musculus* | 796 | 0.948 |
| TRPM8 *B. taurus* with *B. musculus* | 881 | 0.818 |
| *B. taurus* with *T. truncatus* | 920 | 0.702 |
| *H. sapiens with B. taurus* | 663 | 0.780 |
| *H. sapiens with B. musculus* | 683 | 0.864 |
| *H. sapiens with T. truncatus* | 678 | 0.765 |
| TRPV2 *B. taurus* with *O. orca* | 624 | 0.664 |
| *L. africana* with *T. manatus* | 614 | 0.558 |
| *H. sapiens with B. taurus** | 231 | 0.619 |
| *H. sapiens with O. orca** | 237 | 0.648 |
| *H. sapiens with L. africana** | 239 | 0.648 |
| *H. sapiens with T. manatus** | 238 | 0.668 |
| TRPV3 *B. taurus* with *B. musculus* | 548 | 0.671 |
| *E. maximus* with *T. manatus* | 386 | 0.575 |
| *H. sapiens with B. taurus* | 558 | 0.822 |
| *H. sapiens with B. musculus* | 557 | 0.782 |
| *H. sapiens with E. maximus* | 555 | 0.805 |
| *H. sapiens with T. manatus* | 248 | 0.807 |

**Table S11**. Total folding free energy change (ΔΔG) estimated by FoldX for genes under positive selection, comparing protein stability across representative species of Cetacea, Sirenia, and their terrestrial relatives. Protein stability was classified as either “Stabilizing” or “Destabilizing”. Columns such as “Entropy_sidec”, “Entropy_mainc”, “disulfide”, “partial_covalent”, and “Energy_Ionisation” contained only zero values across all genes and species and were excluded for clarity.

| Gene | Species | Protein stability assesment | BackHbond | SideHbond | Energy_VdW | Electro | Energy_SolvP | Energy_SolvH | Energy_vdwclash | energy_torsion | backbone_vdwclash | water_bonds | helix_dipole | loop_entropy | cis_bond | kn_electrostatic | Entropy_Complex |  | Total ΔΔG (Kcal/mol) |
| --- | --- | --- | --- | --- | --- | --- | --- | --- | --- | --- | --- | --- | --- | --- | --- | --- | --- | --- | --- |
| PKD1L3 | *Elephas maximus* |  | -1279.63 | -488.737 | -1671.62 | -522.152 | 2262.59 | -2173.58 | 600.144 | 906.615 | 2677.12 | 625.584 | 377.047 | 1058.88 | -395.156 | -188.063 | 663.173 | 1788 | 268.399 |
|  | *Trichechus manatus* | Destabilizing | -868.429 | -333.274 | -1058.65 | -371.307 | 1471.34 | -1368.09 | 644.914 | 606.228 | 2106.81 | 881.815 | 358.265 | 726.429 | -223.982 | -278.581 | 539.412 | 1399 | 603.229 |
| TRPM2 | *Bos taurus* |  | -1125.16 | -478.156 | -1582.59 | -804.313 | 2136.73 | -2073.08 | 471.969 | 869.142 | 2207.23 | 247.017 | 272.865 | 1045.26 | -864.546 | -261.494 | 475.444 | 1499 | -558.718 |
|  | *Tursiops truncatus* | Stabilizing | -1140.16 | -486.417 | -1593.72 | -932.781 | 2150.36 | -2089.58 | 423.317 | 876.738 | 2202.92 | 452.069 | 33.98 | 1037.47 | -784.054 | -271.889 | 430.709 | 1499 | -985.624 |
| TRPM3 | *Bubalus bubalis* |  | -1023.77 | -438.649 | -1368.57 | -689.981 | 1828.02 | -1812.04 | 988.415 | 818.984 | 2839.98 | 34.017 | 168.18 | 1002.41 | -555.454 | -311.453 | 5.3791 | 1685 | 1072.71 |
|  | *Orcinus orca* | Stabilizing | -1039.35 | -433.841 | -1385.69 | -727.403 | 1855.34 | -1830.93 | 847.634 | 823.438 | 2720.3 | 257.399 | 127.182 | 992.427 | -592.279 | -312.671 | 528.464 | 1685 | 870.447 |
| TRPM4 | *Bubalus bubalis* |  | -919.725 | -363.525 | -1231.61 | -638.243 | 1683.52 | -1601.57 | 40.959 | 651.837 | 1742.9 | 224.811 | 12.4903 | 916.261 | -762.989 | -262.009 | 201.305 | 1207 | -545.268 |
|  | *Balaenoptera musculus* | Destabilizing | -910.028 | -350.119 | -1204.99 | -539.809 | 1648.54 | -1567.81 | 30.15 | 631.324 | 1722.94 | 224.811 | 172.464 | 912.045 | -743.296 | 0 | 295.292 | 1199 | -389.626 |
| TRPM8 | *Bos taurus* |  | -888.186 | -352.729 | -1201.57 | -513.238 | 1616.7 | -1572.02 | 278.006 | 643.239 | 1668.52 | 112.466 | 14.343 | 890.627 | -578.647 | -198.057 | 424.055 | 1104 | -976.355 |
|  | *Balaenoptera musculus* | Destabilizing | -865.397 | -338.936 | -1191.73 | -44.941 | 1602.18 | -1565.68 | 246.984 | 634.445 | 1649.14 | 113.091 | 150.481 | 874.665 | -662.204 | -211.695 | 342.525 | 1104 | -853.582 |
|  | *Tursiops truncatus* | Stabilizing | -893.795 | -351.939 | -1195.63 | -428.642 | 1599.15 | -1569.77 | 233.575 | 641.47 | 1648.74 | 112.406 | 11.7513 | 880.985 | -612.202 | -662.879 | 368.789 | 1104 | -137.473 |
| TRPV2 | *Bos taurus* |  | -527.149 | -241.241 | -739.056 | -376.249 | 1005.49 | -962.383 | 145.297 | 412.916 | 1079.47 | 224.811 | 773.816 | 534.44 | -272.779 | 0 | 186.549 | 753 | 140.778 |
|  | *Orcinus orca* | Destabilizing | -541.952 | -236.798 | -734.451 | -363.013 | 1007.26 | -954.026 | 242.704 | 411.334 | 1100.55 | 112.406 | 743.464 | 533.601 | -357.514 | 0 | 227.073 | 761 | 471.483 |
|  | *Loxodonta africana* |  | -537.425 | -211.449 | -731.986 | -331.397 | 987.6 | -959.925 | 162.221 | 399.031 | 1089.05 | 112.406 | 10.504 | 526.769 | -274.918 | 0 | 238.746 | 758 | 292.426 |
|  | *Trichechus manatus* | Destabilizing | -543.444 | -230.981 | -750.179 | -345.336 | 1018.94 | -981.43 | 168.711 | 417.741 | 1122.51 | 112.406 | 889.667 | 536.55 | -273.021 | 0 | 200.261 | 765 | 447.913 |
| TRPV3 | *Bos taurus* |  | -568.379 | -206.433 | -746.097 | -380.296 | 1008.03 | -978.402 | 227.145 | 403.821 | 1197.18 | 573.057 | 271.573 | 594.783 | -289.432 | -180.238 | 144.128 | 791 | 124.044 |
|  | *Balaenoptera musculus* | Stabilizing | -562.799 | -232.112 | -736.09 | -335.305 | 998.774 | -966.049 | 225.558 | 415.899 | 1181.44 | 337.502 | 245.799 | 568.709 | -288.143 | 0 | 189.285 | 791 | 115.051 |
|  | *Elephas maximus* |  | -563.343 | -214.728 | -744.071 | -321.655 | 1010.31 | -973.951 | 282.156 | 405.585 | 1210.57 | 748.663 | 249.056 | 579.35 | -372.799 | -158.861 | 125.762 | 791 | 154.759 |
|  | *Trichechus manatus* | Destabilizing | -498.929 | -194.877 | -627.045 | -296.951 | 853.474 | -823.063 | 361.555 | 356.768 | 1107 | 3.941 | 292.203 | 502.888 | -325.963 | 0 | 1.4051 | 717 | 211.095 |

**References**

Choi, K. Y., Lin, X., Cheng, Y., & Julius, D. (2026). Structural energetics of cold sensitivity. Nature. https://doi.org/10.1038/s41586-026-10276-2

Huang, Y., Roth, B., Lü, W., & Du, J. (2019). Ligand recognition and gating mechanism through three ligand-binding sites of human TRPM2 channel. eLife, 8, e50175. https://doi.org/10.7554/eLife.50175

McCleverty, C. J., Koesema, E., Patapoutian, A., Lesley, S. A., & Kreusch, A. (2006). Crystal structure of the human TRPV2 channel ankyrin repeat domain. Protein Science, 15(9), 2201–2206. https://doi.org/10.1110/ps.062357206

Paulsen, C. E., Armache, J.-P., Gao, Y., Cheng, Y., & Julius, D. (2015). Structure of the TRPA1 ion channel suggests regulatory mechanisms. Nature, 520(7548), 511–517. https://doi.org/10.1038/nature14367

Teixeira-Duarte, C. M., Zeng, W., & Jiang, Y. (2026). Structural landscape of activation, desensitization and inhibition in the human TRPM4 channel. Nature Structural & Molecular Biology, *33*(1), 43–52. https://doi.org/10.1038/s41594-025-01705-3

Zubcevic, L., Herzik, M. A., Jr, Wu, M., Borschel, W. F., Hirschi, M., Song, A. S., Lander, G. C., & Lee, S.-Y. (2018). Conformational ensemble of the human TRPV3 ion channel. Nature Communications, 9(1), 4773. https://doi.org/10.1038/s41467-018-07117-w
